# Experimental and Theoretical Study of the OH-Initiated Degradation of Piperazine under Simulated Atmospheric Conditions

**DOI:** 10.1021/acs.jpca.0c10223

**Published:** 2020-12-30

**Authors:** Wen Tan, Liang Zhu, Tomas Mikoviny, Claus J. Nielsen, Armin Wisthaler, Barbara D’Anna, Simen Antonsen, Yngve Stenstrøm, Naomi J. Farren, Jacqueline F. Hamilton, Graham A. Boustead, Alexander D. Brennan, Trevor Ingham, Dwayne E. Heard

**Affiliations:** † Section for Environmental Sciences, Department of Chemistry, University of Oslo, P.O. Box 1033, Blindern, NO-0315 Oslo, Norway; ‡ Aix Marseille Univ, CNRS, LCE, UMR 7376, 13331 Marseille, France; § Faculty of Chemistry, Biotechnology and Food Science, Norwegian University of Life Sciences, P.O. Box 5003, N-1432 Ås, Norway; ∥ Wolfson Atmospheric Chemistry Laboratories, Department of Chemistry, University of York, York YO10 5DD, U. K.; ⊥ School of Chemistry, University of Leeds, Leeds LS2 9JT, U. K.

## Abstract

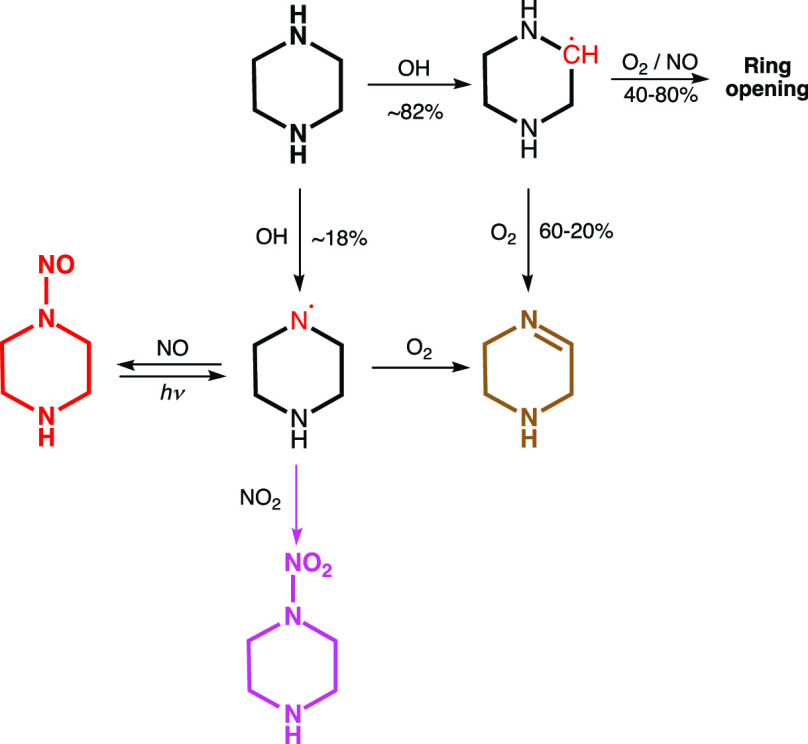

The OH-initiated photo-oxidation of piperazine and 1-nitropiperazine as well as the photolysis of 1-nitrosopiperazine were investigated in a large atmospheric simulation chamber. The rate coefficient for the reaction of piperazine with OH radicals was determined by the relative rate method to be *k*_OH-piperazine_ = (2.8 ± 0.6) × 10^–10^ cm^3^ molecule^–1^ s^–1^ at 307 ± 2 K and 1014 ± 2 hPa. Product studies showed the piperazine + OH reaction to proceed both via C–H and N–H abstraction, resulting in the formation of 1,2,3,6-tetrahydropyrazine as the major product and in 1-nitropiperazine and 1-nitrosopiperazine as minor products. The branching in the piperazinyl radical reactions with NO, NO_2_, and O_2_ was obtained from 1-nitrosopiperazine photolysis experiments and employed analyses of the 1-nitropiperazine and 1-nitrosopiperazine temporal profiles observed during piperazine photo-oxidation. The derived initial branching between N–H and C–H abstraction by OH radicals, *k*_N–H_/(*k*_N–H_ + *k*_C–H_), was 0.18 ± 0.04. All experiments were accompanied by substantial aerosol formation that was initiated by the reaction of piperazine with nitric acid. Both primary and secondary photo-oxidation products including 1-nitropiperazine and 1,4-dinitropiperazine were detected in the aerosol particles formed. Corroborating atmospheric photo-oxidation schemes for piperazine and 1-nitropiperazine were derived from M06-2X/aug-cc-pVTZ quantum chemistry calculations and master equation modeling of the pivotal reaction steps. The atmospheric chemistry of piperazine is evaluated, and a validated chemical mechanism for implementation in dispersion models is presented.

## 1 Introduction

Piperazine (1,4-diazacyclohexane, PZ) is among the amines considered for use in large-scale Carbon Capture (CC) to reduce CO_2_ emissions from industrial point sources.^1^ A 40 wt % amine solution with PZ and 2-amino-2-methyl-1-propanol in a 1:2 M ratio was recently suggested as the new benchmark solvent for CO_2_ capture technology.^2^

Measurements at the Technology Centre Mongstad (TCM; Norway) have established that at times it can be difficult to avoid ppm-level emissions of amines and their process degradation products to the environment during operation of a large-scale capture plant^3^—the concern being that carcinogenic nitrosamines and nitramines are either directly emitted or formed in the subsequent atmospheric photo-oxidation of the fugitive amines.^4^ The Norwegian Institute for Public Health recommends that the total amount of nitrosamines and nitramines in the atmosphere should be below 0.3 ng m^–3^ in air and below 40 ng dm^3^ in drinking water for a risk level of 10^–5^.^4^ Such low detection levels are currently virtually impossible to monitor with today’s technology, and it is consequently imperative to acquire quantitative information on the degradation pathways for the relevant amines under atmospheric conditions and to implement this information in reliable chemical models for dispersion calculations.

The major removal processes of gaseous PZ in the atmosphere are uptake in aqueous particles and gas phase reaction with OH radicals during daytime and NO_3_ radicals during nighttime. The OH radical reaction with PZ was recently reported to be very fast, ∼2.3 × 10^–10^ cm^3^ molecule^–1^ s^–1^ at 298 K and to favor C–H abstraction: *k*_N–H_/(*k*_N–H_ + *k*_C–H_) = 0.09 ± 0.06.^5^

The PZ nitrosamine (1-nitrosopiperazine, PZNO) and nitramine (1-nitropiperazine, PZNO_2_) are both carcinogenic;^4^ they result from the following sequence of atmospheric gas-phase reactions^6^
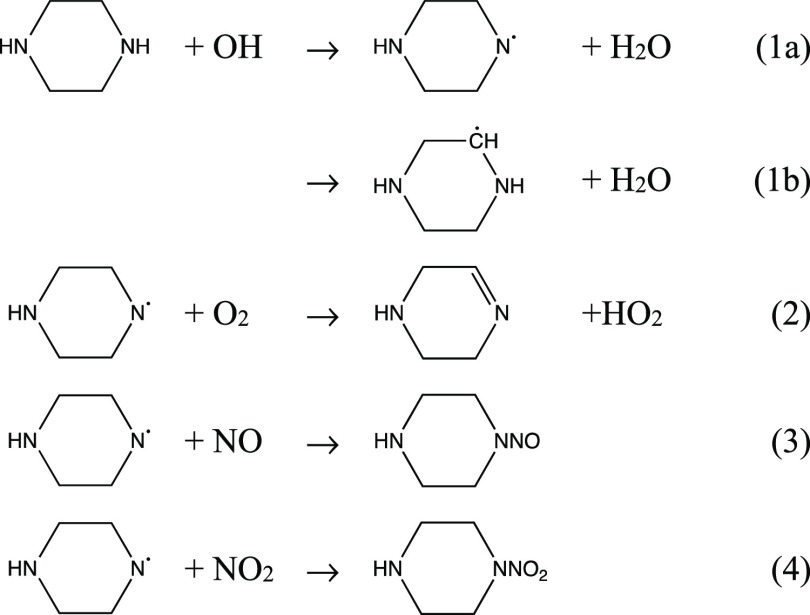
1a

Although the O_2_ reaction with aminyl radicals, R_1_R_2_Ṅ, is reported to be around 6 orders of magnitude slower than the corresponding NO and NO_2_ reactions,^7^ it is still dominating under most atmospheric conditions, and PZNO and PZNO_2_ are thus only expected as minor products in the natural atmospheric photo-oxidation of PZ. Both compounds were observed, but not quantified, in previous PZ photo-oxidation experiments in the ∼200 m^3^ European Photoreactor (EUPHORE),^8^ and in the more recent experiments employing a ∼18 m^3^ indoor smog chamber.^9^

The open literature includes two theoretical studies on the kinetics of the hydrogen abstraction from PZ by OH radicals, in which the branching between the N–H and C–H abstraction reactions 1a and 1b were predicted to be 0.07^10^ and 0.01,^11^ respectively, at 298 K. The latter theoretical study also includes an investigation of the atmospheric degradation following the C–H abstraction. A theoretical report of the Cl-atom-initiated oxidation of PZ suggests that this reaction proceeds with 99.8% N–H abstraction at 298 K;^12^ the study also includes a mapping of the potential energy surfaces for the piperazinyl radical reactions with NO and O_2_.

In the present communication, we report results from a series of PZ and PZNO_2_ photo-oxidation and PZNO photolysis experiments in the EUPHORE chamber, and quantum chemistry-based evaluations of the major routes in the OH initiated photo-oxidations of PZ and PZNO_2_ under atmospheric conditions. The new results pave the way for the first reliable environmental impact assessments of realizing large-scale CC-facilities based on PZ-containing solvents.

## 2 Methods

### 2.1 Experimental Methods and Chemicals

A series of experiments was carried out in chamber B of the EUPHORE facility in Valencia, Spain. The facility and analytical methods have recently been reported in detail^13^—special on-line instrumentation include a PTR-TOF 8000 instrument (IONICON Analytik GmbH, Innsbruck, Austria), a prototype CHARON inlet^14,15^ interfaced to a second PTR-TOF 8000, a compact time-of-flight Aerosol Mass Spectrometer (C-ToF-AMS, Aerodyne Research Inc., Billerica, MA, U.S.A.),^16^ and a FAGE (Fluorescence Assay by Gas Expansion) apparatus.^17^ Additional information specific to the present work is given in the Supporting Information.

Information on chemicals used and the synthesis of PZNO and PZNO_2_ is found in the Supporting Information.

### 2.2 Computational Methods

Optimized geometries of stationary points on the potential energy surfaces for the atmospheric degradation of PZ were obtained in M06-2X^18^ calculations employing the aug-cc-pVTZ^19,20^ basis set. Pre- and postreaction complexes were located by following the intrinsic reaction coordinate^21−24^ from the saddle points. Electronic energies of selected stationary points were improved by explicitly correlated coupled cluster calculations with scaled triples contributions, denoted CCSD(T*)-F12a.^25,26^ Reaction enthalpies and proton affinities were calculated using the G4 model chemistry.^27^ Dipole moments and isotropic polarizabilities, serving as input to prediction of ion-molecule reaction rate coefficients,^28^ were obtained in M062X/aug-cc-pVTZ and B3LYP/aug-cc-pVTZ calculations; see Table S1 in the Supporting Information. The M06-2X, B3LYP, and G4 calculations were performed in Gaussian 09;^29^ the CCSD(T*)-F12a calculations were carried out employing Molpro 2012.1.^30,31^

Master equation calculations were carried out using the program MESMER 3.0^32^ (Master Equation Solver for Multi-Energy-Well Reactions) to simulate the reactions under atmospheric conditions. The required input parameters for molecules, intermediate species, and products were obtained from the ab initio calculations.

## 3 Results

### 3.1 Computational Results

The kinetics of the initial step in the PZ + OH reaction is complicated by PZ existing in three low-energy chair conformations (*eq*-*eq*, *eq*-*ax*, and *ax*-*ax*) with relative enthalpies of 0, 2.44, and 6.92 kJ mol^–1^, respectively (values from G4 calculations). Consequently, the conformational equilibrium will consist of around 55% *eq*-*eq*, 42% *eq*-*ax*, and 3% *ax*-*ax* at 298 K. This issue was not considered in the previous theoretical studies of the reaction, and a detailed theoretical account of the kinetics and of the branching between C–H and N–H abstraction in the initial step is far from trivial and considered outside the scope of the present work.

**Scheme 1:**
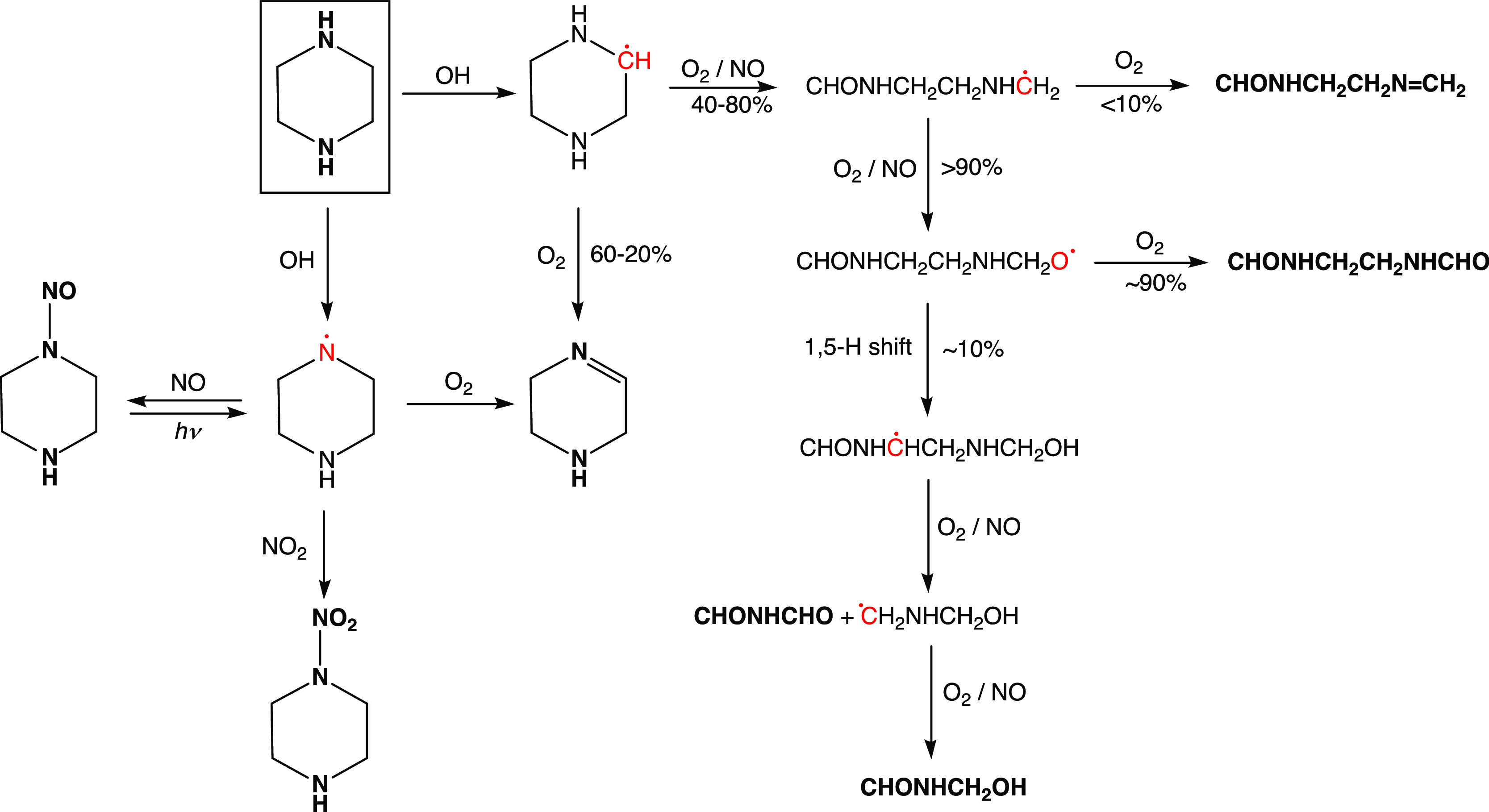
Quantum Chemistry Prediction of the Major Primary Products in the OH-Initiated Atmospheric Photo-Oxidation of Piperazine (PZ) Radical sites are indicated with red, thermally stable molecules are shown in boldface.

The theoretical prediction of the major routes in the atmospheric degradation of PZ is summarized in Scheme 1. The degradation routes largely concord with those established in previous dimethylamine^7,33,34^ and diethylamine^8,33^ photo-oxidation experiments. Details of the quantum chemistry study are collected in the Supporting Information, including illustrations of the pivotal potential energy surfaces, Figures S1–S5, and the associated Tables S2–S6 containing energies, Cartesian coordinates, and vibration-rotation data employed in master equation calculations.

The present mechanistic assessment differs notably from that recently offered based on G4 calculations.^11^ First, our study includes a mapping of the atmospheric PZ aminyl radical reactions under atmospheric conditions suggesting a slightly different, and simpler scheme than that first suggested and applied by Lindley et al.^7^ in their analysis of the (CH_3_)_2_Ṅ radical reactions with O_2_, NO and NO_2_. The difference being that the piperazinyl + NO_2_ reaction leading to the corresponding imine is blocked by a barrier of around 12 kJ mol^–1^ above the entrance energy of the reactants. Another result from the present theoretical study is that the barrier to reaction 2 is calculated to be ∼10 kJ mol^–1^ higher than in the corresponding (CH_3_)_2_Ṅ + O_2_ reaction, indicating that PZ has a higher potential to nitrosamine and nitramine formation than dimethylamine per aminyl radical.

Second, we find the cyclic alkoxy radical, that ultimately follows C–H abstraction, to be metastable resulting in spontaneous ring opening, and that the major fraction of the resulting CHONHCH_2_CH_2_NHĊH_2_ radical will end up as a diamide. The calculated branching between ring-opening and formation of the PZ imine, 1,2,3,6-tetrahydropyrazine (PZI), is very sensitive to the barrier height and cannot be accurately predicted from theoretical calculations. In summary, the present theoretical study predicts that under ambient conditions with NO > 2 ppb, the major products following C–H abstraction from PZ will be 60–20% PZI, 32–65% CHONHCH_2_CH_2_NHCHO, 4–8% CHONHCH_2_CH_2_N=CH_2_, and 4–7% CHONHCHO and CHONHCH_2_OH.

Third, we have also assessed the atmospheric fate of PZNO_2_—one of the carcinogenic PZ photo-oxidation products. The major photo-oxidation routes for PZNO_2_, outlined in Scheme S1 in the Supporting Information, parallel to those of PZ with one exception—the alkyl-radical formed upon ring-opening ejects NO_2_ resulting in the same amide/imine that was also predicted as a primary product in the PZ + OH reaction. Details of the quantum chemistry study of the OH radical-initiated atmospheric PZNO_2_ photo-oxidation are found the Supporting Information (including Figure S6 illustrating the potential energy surface to ring-opening and subsequent NO_2_-ejection, and the underlying quantum chemistry data in Table S7).

Previous photo-oxidation studies of PZ have demonstrated not only experimental challenges but also disagreement in the understanding of the underlying mechanism.^8,9^ The present theoretical study offers a detailed mechanistic insight and an accurate prediction of the product distribution, facilitating a comprehensive interpretation of the experimental photo-oxidation experiments which are described below.

### 3.2 Experimental Results

We first report results from kinetic studies of the PZ + OH reaction. We then present results from PZNO_2_ photo-oxidation experiments and from PZNO photolysis experiments facilitating interpretation of the pièce de résistance—the atmospheric PZ photo-oxidation. Finally, we present results from studies of the aerosol formed in the PZ photo-oxidation experiments.

#### 3.2.1 Piperazine + OH Reaction Kinetics

Two relative rate experiments were carried out in the EUPHORE chamber B in which isoprene, limonene, 1,3,5-trimethylbenzene, and pyrrole were used as reference compounds. Acetonitrile was added as an inert tracer to monitor the apparent dilution by purified air that is constantly added to compensate for leakage and continuous sampling by the air monitors (*k*_OH+CH_3_CN_ = 2.2 × 10^–14^ cm^3^ molecule^–1^ s^–1^ at 298 K).^35^ OH radicals were generated employing IPN as the precursor: CH_3_CH(ONO)CH_3_ + hν → CH_3_CH(Ȯ)CH_3_ + NO; CH_3_CH(Ȯ)CH_3_ + O_2_ → CH_3_C(O)CH_3_ + HO_2_; HO_2_ + NO → OH + NO_2_.

**Figure 1:**
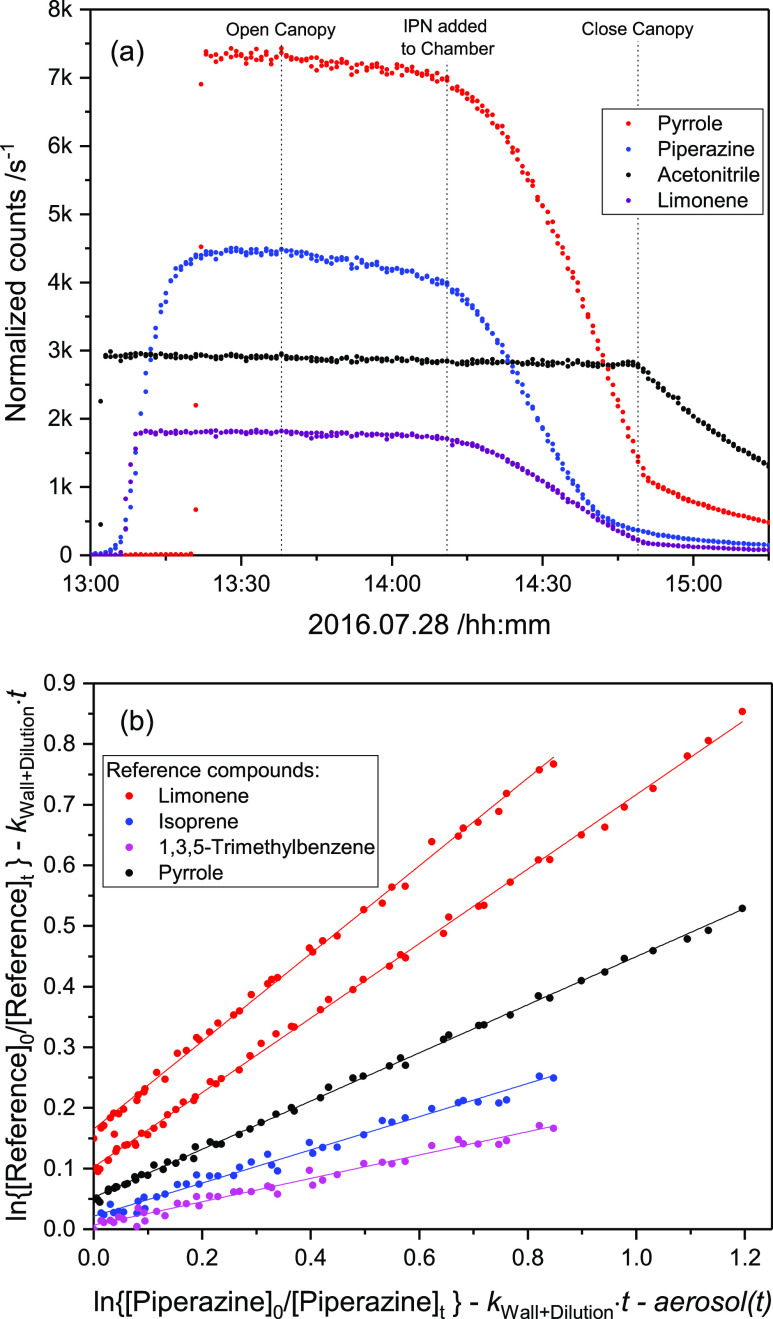
(a): Time evolution of the acetonitrile, pyrrole, PZ and limonene ion signals at *m*/*z* 42.034, 68.050, 87.092, and 137.133, respectively, during the second kinetic experiment on 2016.07.28. (b): Relative rate plot showing the decays of isoprene, limonene, pyrrole, and piperazine at 1014 hPa and 307 K in the presence OH radicals. For the sake of clarity, the data have been displaced along the abscissa. The data have been corrected for dilution because of chamber air replenishment, for wall loss and for loss to the aerosol; see Supporting Information.

Figure 1a displays the time evolution of compound-specific PTR–ToF–MS ion signals measured during the second experiment (the first experiment is documented in Figure S7, Supporting Information). The dilution rate because of air replenishment was 8.6 × 10^–6^ s^–1^ in the two experiments; PZ wall loss rates (derived from the reagent decay prior to adding IPN) ranged from 1 to 4 × 10^–5^ s^–1^. Initial mixing ratios were ∼100 ppb for the reference compounds and ∼200 ppb for PZ. Average OH densities in the EUPHORE chamber during the periods selected for analyses (9:10—9:30 and 14:10—14:35 UTC) were around 3 × 10^6^ cm^–3^; average pressure and temperature in the two experiments were 1014 ± 2 mbar and 307 ± 2 K. The temporal profile of PZ recorded by the PTR–ToF–MS matches well the one obtained by a home-built high-temperature PTR–MS, indicating an adequate instrument response time for “sticky” substances such as PZ (Figure S8 in the Supporting Information).

A least–squares fitting of the wall- and dilution loss-corrected data (Figure S9 in the Supporting Information) results in an average *k*_OH+PZ_ = (3.0 ± 0.6) × 10^–10^ cm^3^ molecule^–1^ s^–1^ at 307 ± 2 K and 1014 ± 2 hPa. Considerable amounts of PZ are, however, transferred from the gas to the particle phase during the periods selected for analysis. Figures S10, S11 (Supporting Information) show the time evolution of aerosol mass and the aerosol PZ content during the kinetic experiments; approximately 6.3 and 1.2% of PZ were lost to the aerosol particles during the two kinetic experiments. Correction for PZ loss to particles during the kinetic experiments was therefore implemented in the data analysis (see Supporting Information for details), resulting in an average *k*_OH+PZ_ = (2.8 ± 0.6) × 10^–10^ cm^3^ molecule^–1^ s^–1^ at 307 ± 2 K and 1014 ± 2 hPa, Figure 1b. The present result agrees well with those of Onel et al.,^5^ who reported *k*(T) = (2.37 ± 0.03) × 10^–10^ (T/298)^−(1.76±0.08)^ and *k*_OH+PZ_ = (2.25 ± 0.28) × 10^–10^ cm^3^ molecule^–1^ s^–1^ at 307 K from flash photolysis/resonance fluorescence experiments.

#### 3.2.2 1-Nitropiperazine Photo-Oxidation Studies

The atmospheric fate of PZNO_2_ was investigated in two photo-oxidation experiments under high NO and high NO_2_ starting conditions, respectively. This parent compound as well as its degradation products are very “sticky” and transfer relatively fast to the chamber walls. In addition, the PZNO_2_ photo-oxidation experiments were accompanied by strong particle formation with ∼50% of the initial PZNO_2_ mass being transferred to particles (see Figure S12 in the Supporting Information). This makes quantitative conclusions impossible.

**Figure 2:**
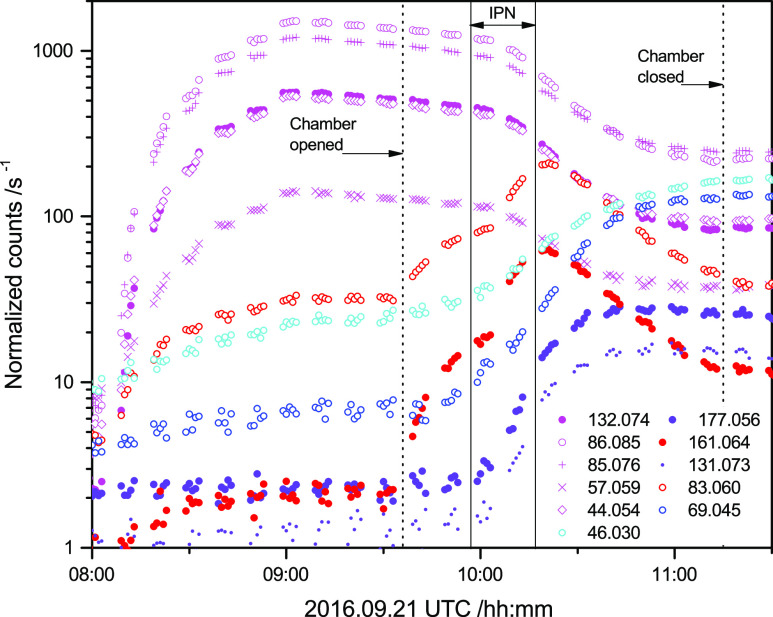
Time profiles of selected ion signals detected during the 1-nitropiperazine (PZNO_2_) photo-oxidation experiment on 2016.09.21. Drift tube electric field *E*/*N* = 105 Td.

Figure 2 shows time profiles of the selected mass peaks observed during the high-NO photo-oxidation experiment. It is worth noting that protonated PZNO_2_ fragments severely at the PTR–ToF–MS instrumental settings employed (*E*/*N* = 105 Td): 15% *m*/*z* 132.077 (protonated molecule), 38% *m*/*z* 86.084 (NO_2_ ejection), 30% *m*/*z* 85.076 (HONO ejection), 4% *m*/*z* 57.057 (C_3_H_7_N^+^, ring fragment), and 13% *m*/*z* 44.050 (C_2_H_6_N^+^, ring fragment). At *E*/*N* = 65 Td, the fragmentation is less pronounced: 44% *m*/*z* 132.077, 48% *m*/*z* 86.084, 8% *m*/*z* 85.077, <1% *m*/*z* 57.057, and <1% *m*/*z* 44.050. Consistent concentrations of PZNO_2_ were derived from both *E*/*N* settings. The mass peaks related to PZNO_2_ photo-oxidation are summarized in Table S8 in the Supporting Information.

Figure 2 also demonstrates that PZNO_2_ is quite reactive. Kinetic data for the CH_3_NHNO_2_^36^ and (CH_3_)_2_NNO_2_^36,37^ reaction with OH show an order of magnitude reduction in reactivity vis-à-vis the parent amines.^38^ Apparently, the deactivating reactivity effect of the electron withdrawing nitro group does not extend beyond the adjacent methylene groups in PZNO_2_.

The 1-nitroso-4-nitropiperazine ([PZ(NO)NO_2_]H^+^, *m*/*z* 161.067) signal appears the very moment the chamber canopy is opened, and it is highly significant that this is paralleled by the *m*/*z* 83.060 peak. Upon injection of IPN, the increase in the *m*/*z* 177.062 ion signal, which is unique to 1,4-dinitropiperazine [PZ(NO_2_)_2_], is particularly illustrative. In line with the extensive fragmentation of protonated PZNO_2_, most of the other ion signals observed during the two photo-oxidation experiments correspond to molecular fragments, Table S8. The *m*/*z* 46.029 (CH_4_NO^+^) and 69.045 (C_3_H_5_N_2_^+^) signals grow throughout the experiments. The former could originate from formamide, the latter from imidazole. There are no obvious gas phase photo-oxidation routes leading from PZNO_2_ to these compounds or to their isomers, and we tentatively attribute their formation to heterogeneous chemistry; see later.

It is somewhat surprising that the expected major product following C–H abstraction—the imine, 1-nitro-1,2,3,6-tetrahydropyrazine (PZINO_2_)—is not revealed by even a trace of the protonated molecule at *m*/*z* 130.061. Assuming a similar fragmentation of protonated PZINO_2_ as observed for protonated PZNO_2_, fragment ions are expected at *m*/*z* 84.068 (NO_2_ ejection), 83.060 (HONO ejection), 55.042 (CH_2_CH_2_N=CH^+^, ring fragment), and 42.034 (CH_2_CH_2_N^+^, ring fragment). There is no ion signal detected at *m*/*z* 84.068, but the *m*/*z* 83.060, 55.042, and 42.034 ion signals are all observed having the expected time profile, Figure 2. Although the experimental data are not unambiguously conclusive, we hypothesize that these mass peaks are more than just indicative of the imine being formed in the PZNO_2_ photo-oxidation.

#### 3.2.3 1-Nitrosopiperazine Photolysis Studies

Nitrosamines have a characteristic *n* → π* transition in the UV-A region and photolyze rapidly in natural sunlight; the quantum yield to photo-dissociation of (CH_3_)_2_NNO following S_0_ → S_1_(*n*π*) excitation at 363.5 nm was reported to be 1.03 ± 0.10,^39^ and theory shows that the excited S_1_ state is repulsive leading to swift dissociation following excitation.^40^ In the present case, the two primary products expected following PZNO photolysis are PZI and PZNO_2_, Scheme 1.

Three photolysis experiments were carried out in the EUPHORE chamber B. Cyclohexane was added to the chamber (∼2 ppm) for deriving the amount of OH radicals formed following PZNO photolysis: PZNO + hν → PZ^•^ + NO; PZ^•^ + O_2_ → PZI + HO_2_; HO_2_ + NO → OH + NO_2_. The derived OH radical mixing ratio varied between 1 and 4 × 10^5^ cm^–3^ (for details, see Figures S13–S15 and accompanying text in the Supporting Information).

**Figure 3:**
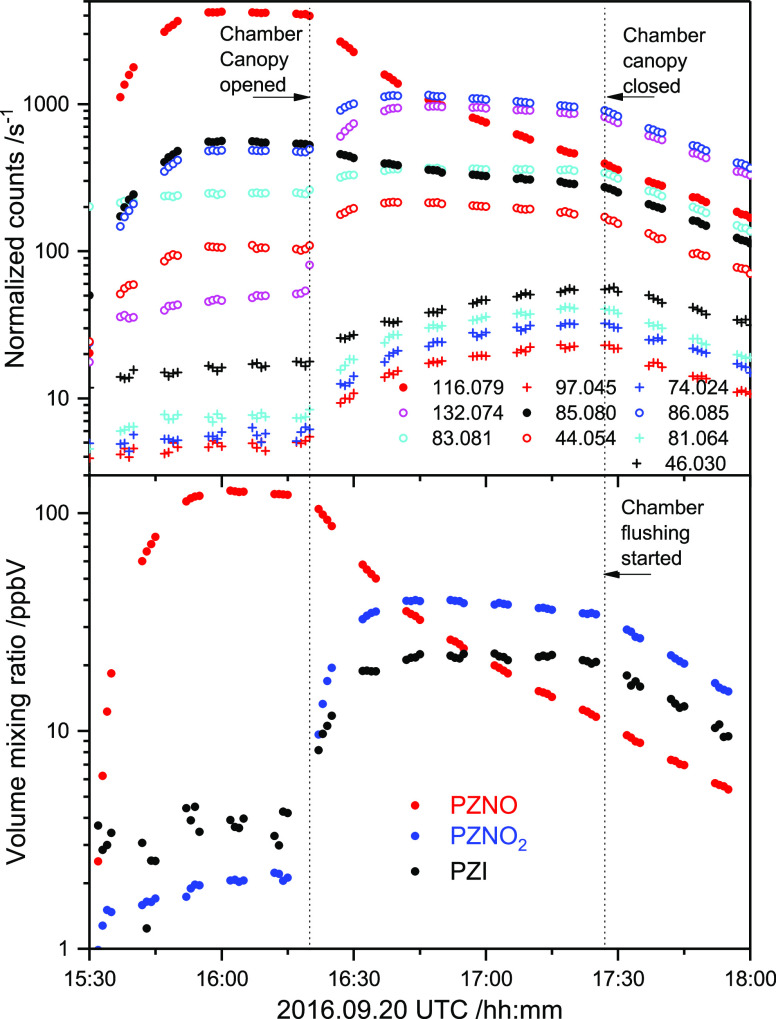
Top: time profiles of ion signals detected during the 1-nitrosopiperazine (PZNO) photolysis experiment on 2016.09.20. Only ion signals increasing by more than 1% of the *m*/*z* 116.079 [PZNO]H^+^ ion signal decrease are included. Drift tube electric field: *E*/*N* = 65 Td. Bottom: Derived volume mixing ratios (ppbV) of 1-nitrosopiperazine (PZNO), 1-nitropiperazine (PZNO_2_), and 1,2,3,6-tetrahydropyrazine (PZI) during the experiment.

Figure 3 illustrates the ion signal time profiles observed during the experiments. The mass peaks pertinent to the PZNO photolysis experiments are summarized in Table 1; a more complete list of ion signals observed in the experiments is found in Table S9 in the Supporting Information, which also includes data from our previous study in which we employed a PZNO sample of different origin.^8^ It can be seen from Figure 3 that the mass peaks growing in upon photolysis fall in three categories: (1) the *m*/*z* 116.082 and 85.076 that decrease in intensity when the chamber is opened to sunlight, (2) the *m*/*z* 132.077, 86.084, 83.060, and 44.050 having time profiles typical of primary photolysis products, and (3) the less intense *m*/*z* 97.040, 81.045, 74.024 and 46.029 with time profiles more resembling those of “secondary” products resulting from PZNO, PZNO_2_, and PZI reactions with OH radicals.

**Table 1:** Relevant Mass Peaks Detected by PTR–ToF–MS During 1-Nitrosopiperazine (PZNO) Photolysis Experiments

*m*/*z*	ion sum formula	interpretation
44.050	C_2_H_6_N^+^	fragment from [PZNO]H^+^, [PZNO_2_]H^+^ and [PZI]H^+^
83.060	C_4_H_7_N_2_^+^	H_2_ elimination from [PZI]H^+^
85.076	C_4_H_9_N_2_^+^	[PZI]H^+^, fragment from [PZNO]H^+^ and [PZNO_2_]H^+^
86.084	C_4_H_10_N_2_^+^	fragment from [PZNO]H^+^, [PZNO_2_]H^+^
116.082	C_4_H_10_N_3_O^+^	[PZNO]H^+^
132.077	C_4_H_10_N_3_O_2_^+^	[PZNO_2_]H^+^

An inspection of the ion signals observed in the time period before opening the chamber canopy (Figure 3) reveals that also [PZNO]H^+^ fragments at the instrumental settings employed (*E*/*N* = 65 Td): 78.5% *m*/*z* 116.082 (protonated molecule), 9.8% *m*/*z* 86.084 (NO ejection), 9.5% *m*/*z* 85.076 (HNO ejection), and 2.2% *m*/*z* 44.050 (C_2_H_6_N^+^ ring fragment). At *E*/*N* = 105 Td, the fragmentation is more severe: 62.8% *m*/*z* 116.082, 12.6% *m*/*z* 86.084, 19.8% *m*/*z* 85.076, and 4.8% *m*/*z* 44.050. Consistent concentration of PZNO was derived using both *E*/*N* settings.

Figure 3 further reveals that the expected ion signal of protonated PZI at *m*/*z* 85.076 (C_4_H_9_N_2_^+^), to which fragments of both protonated PZNO and PZNO_2_ contribute, apparently shows more resemblance to that of PZNO than to that of a primary product like PZI or PZNO_2_.

The fragmentation of protonated PZNO and PZNO_2_ complicates an unambiguous identification of PZI from the PTR–TOF–MS data: the ion signals at *m*/*z* 44.050, 85.076, and 86.084 all originate in both PZNO and PZNO_2_. Assuming that PZNO, PZNO_2_, and PZI are neither lost to the chamber walls nor to the aerosol phase in large amounts during the time of photolysis, it is possible to obtain a hypothetical [PZI]H^+^ ion signal using the PZNO and PZNO_2_ fragmentations previously determined. The *m*/*z* 86.084 is fully accounted for by PZNO and PZNO_2_, whereas the *m*/*z* 44.050 (C_2_H_6_N^+^) also includes the contribution from a ring scission fragment of [PZI]H^+^, and the *m*/*z* 83.060 (C_4_H_7_N_2_^+^) is explained by H_2_-loss from [PZI]H^+^.

Figure 3 also includes the derived volume mixing ratios of PZNO, PZNO_2_, and PZI. The gas-phase mass balance in the photolysis experiment shown is only around 60%, but more than half of the missing mass can be accounted for by OH reactions with PZNO, PZNO_2_, and PZI, and partitioning to wall surfaces and to particle formation; see later.

Two of the three photolysis experiments were modelled according to Scheme 1 taking the monitor values for NO, NO_2_, and *j*_NO_2__, and the derived OH-fields as input (the third experiment was carried out under conditions that did not allow quantification of the actinic flux in the chamber). Alike the nitro group, the nitroso group reduces the OH reactivity of (CH_3_)_2_NNO,^37,41^ by an order of magnitude vis-à-vis that of the parent amine.^38^ The OH rate coefficients for PZNO and PZNO_2_, and, for the sake of simplicity, also for PZI were therefore fixed in the model to 1/2 × *k*_OH+PZ_. The rate coefficient for PZNO wall loss was determined to be 4 × 10^–5^ s^–1^ from the sample decay prior to opening the chamber canopy; the same value was assumed to apply for PZNO_2_ and PZI. Attempts to determine the relative photolysis rate coefficient, *j*_rel_ = *j*_PZNO_/*j*_NO_2__, from the available data showed a correlation of 0.99 between *j*_rel_ and *k*_2_/*k*_4_. Consequently, *j*_rel_ was constrained to 0.34—the average value reported for other nitrosamines^8^—and only *k*_2_/*k*_4_ and *k*_3_/*k*_4_ were refined in a non-linear least-squared fitting of the experimental data. The derived parameters, *k*_2_/*k*_4_ = 1.7 ± 0.3 and *k*_3_/*k*_4_ = (1.57 ± 0.06) × 10^–7^ (2σ error limits), fall in the range reported from other nitrosamine photolysis studies,^8^ but they should not be compared directly as the chemistry models differ.

**Figure 4:**
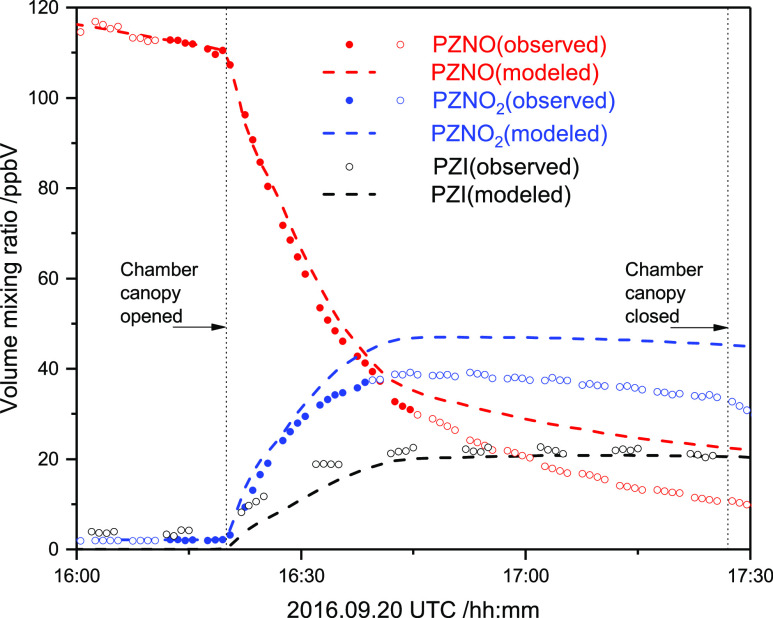
Observed and modelled 1-nitrosopiperazine photolysis under natural sunlight conditions. Observations included in fitting procedure are marked as solid bullets. Abbreviations: PZNO, 1-nitrosopiperazine; PZNO_2_, 1-nitropiperazine; PZI, 1,2,3,6-tetrahydropyrazine.

Figure 4 illustrates the quality of PZNO photolysis modeling under natural sunlight conditions during the afternoon of 2016.09.20 (the other experiment is documented in Figure S16 in the Supporting Information). The agreement between the experiment and model is reasonable considering the model constraints, the inherent uncertainties in the monitor values for NOx and the actinic flux, and the transfer to the aerosol phase, as illustrated in Figure S17 in the Supporting Information. Nearly 10% of the total PZNO/PZNO_2_/PZI mass is transferred to the aerosol during the experiment, and the model indicates that total loss of PZNO/PZNO_2_/PZI to the walls and to reaction with OH radicals amounts to ∼8% each. Finally, we note that there is also a pleasing agreement between the indirectly determined PZI mixing ratios in the photolysis experiments and the modelled PZI mixing ratio, lending confidence to the ion signal interpretation, as presented in Table 1.

#### 3.2.4 Piperazine Photo-Oxidation Studies

**Figure 5:**
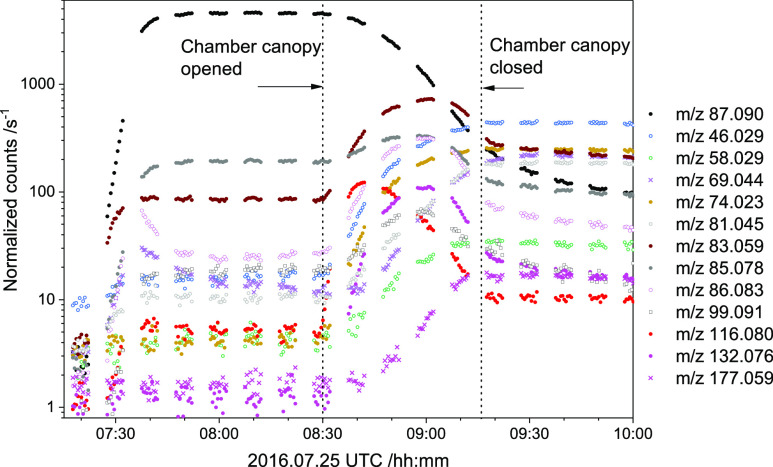
Time evolution of ion signals during the piperazine photo-oxidation experiment on 2016.07.25. With the exception of *m*/*z* 177.059 (1,4-dinitropiperazine), ion signals increasing by less than 2% of the piperazine *m*/*z* 87.090 signal decrease have been omitted for the sake of clarity. Drift tube electric field *E*/*N* = 105 Td.

Previous PZ photo-oxidation experiments carried out in the EUPHORE^8^ and the CSIRO^9^ chambers were severely affected by both wall adsorption/desorption and particle formation. The present series of PZ photo-oxidation experiments was carried out under warmer conditions reducing the wall effects (Table S10 in the Supporting Information summarizes the initial conditions in each of the EUPHORE experiments). Figure 5 exemplifies the observed time evolution of the major ion signals recorded during a photo-oxidation experiment—for the sake of clarity, only ion signals changing by more than 2% of the change in the piperazine signal *m*/*z* 87.092 are included in the Figure. The temporal variation in the NO and NO_2_ mixing ratios and in *j*_NO2_ are documented in Figure S18 in the Supporting Information. The mass peaks pertinent to the PZ photo-oxidation experiments are summarized in Table 2, which also quotes results from the CSIRO experiments^9^ (Tenax sampling, TD-GCMS); a list of ion signals observed in the new as well as in the previous experiments are collected in Table S11 in the Supporting Information; a cleaned PTR mass spectrum is presented in Figure S19. The availability of data obtained during different years employing different samples and different injection techniques facilitated differentiation between genuine and spurious mass peaks not related to the PZ photo-oxidation per se.

**Figure 6:**
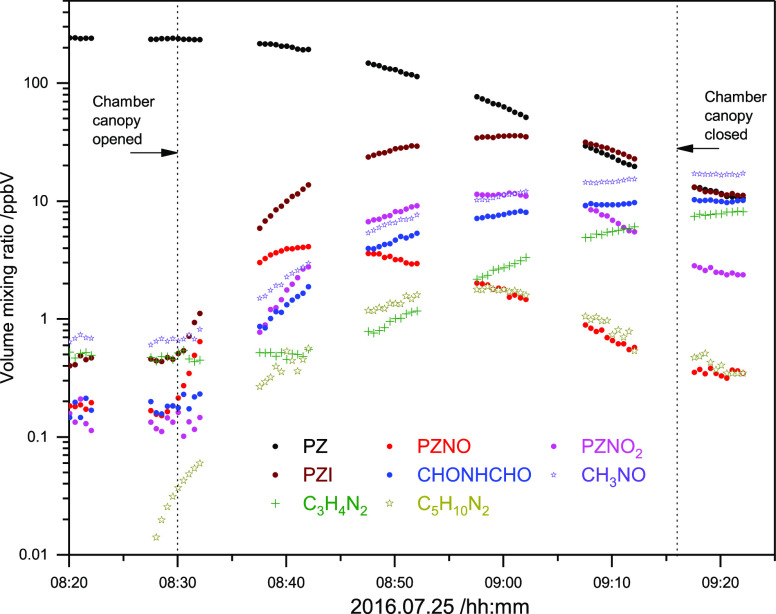
Derived volume mixing ratios (ppbV) of piperazine and observed photo-oxidation products during the experiment on 2016.07.25. Abbreviations: PZ, piperazine; PZNO_2_, 1-nitropiperazine, PZNO, 1-nitrosopiperazine; PZI, 1,2,3,6-tetrahydropyrazine; CH_3_NO, formamide and isomers; C_3_H_4_N_2_, imidazole and isomers; C_5_H_10_N_2_, unidentified condensation product.

**Table 2:** Major PTR–TOF–MS Ion Signals Observed During OH Initiated PZ Photo-Oxidation Experiments^a^

exact	ion sum		fragmentation^b^				
*m*/*z*	formula		PZ	PZI	PZNO	PZNO_2_	interpretation
44.050	C_2_H_6_N^+^		1	12	5	13	ring fragment, aziridine
46.029	CH_4_NO^+^	*^b^					NH_2_CHO and isomers from heterogeneous reactions, chamber artefact?
69.045	C_3_H_5_N_2_^+^						imidazole from heterogeneous reactions
74.024	C_2_H_4_NO_2_^+^	*					CHONHCHO, primary product
81.045	C_4_H_5_N_2_^+^	*	?	?			pyrazine, dehydrogenation fragment from [PZI]H^+^ and [PZ]H^+^; PZ impurity?
83.060	C_4_H_7_N_2_^+^	*	2	84			PZ and PZI dehydrogenation fragment
85.076	C_4_H_9_N_2_^+^		3	4	20	30	PZI. fragment of PZ, PZNO, and PZNO_2_
86.084	C_4_H_10_N_2_^+^				12	38	PZNO and PZNO_2_ fragment
87.092	C_4_H_11_N_2_^+^		94				PZ
99.055	C_4_H_7_N_2_O^+^	*					dihydropyrazinone isomers, oxidation product of PZI?
99.092	C_5_H_11_N_2_^+^	*					unidentified condensation product
115.087	C_5_H_11_N_2_O^+^	*					1-formylpiperazine (cond. prod.)
116.082	C_4_H_10_N_3_O^+^	*			63		PZNO
132.077	C_4_H_10_N_3_O_2_^+^	*				15	PZNO_2_
177.062	C_4_H_9_N_4_O_4_						PZ(NO_2_)_2_

a Only ion signals increasing by more than 2% of the *m*/*z* 87.092 ion signal decrease are included. Abbreviations: PZ, piperazine; PZI, 1,2,3,6-tetrahydropyrazine; PZNO, 1-nitrosopiperazine; PZNO_2_, 1-nitropiperazine.

b Fragmentation in % at *E*/*N* = 105 Td. Corresponding molecular formula found by TD-GCMS of Tenax samples, ref (9).

The ion signals can be grouped according to their time evolution: (1) signals that appear upon injection of PZ along with that of *m*/*z* 87.090—protonated PZ, (2) signals that grow and decrease again during the photo-oxidation experiment (reactive primary products), and (3) signals that grow steadily after opening the chamber canopy (secondary products and chamber artefacts).

The group (1) signals indicate that [PZ]H^+^ fragments at the instrumental conditions are employed in the present experiments—although not as severely as protonated PZI, PZNO_2_, and PZNO. Analyses of the time periods before photo-oxidation reveals 94% *m*/*z* 87.092 (protonated molecule), 3% *m*/*z* 85.076 (C_4_H_9_N_2_^+^, H_2_-loss), 2% *m*/*z* 83.060 (C_4_H_7_N_2_^+^, twofold H_2_-loss), and 1% *m*/*z* 44.050 (C_2_H_6_N^+^, ring fragment) employing a drift tube *E*/*N* = 65 Td. In addition, there is an initially correlated mass peak ∼0.2% at *m*/*z* 81.045 (C_4_H_7_N_2_^+^) attributed to protonated pyrazine that may be a sample impurity. Note, however, that *m*/*z* 81.044 increases in intensity throughout the PZ photo-oxidation experiments, and that it also grows in the PZNO_2_ and PZNO experiments.

The group (2) signals include *m*/*z* 132.077, 116.082, 99.092, 86.084, 85.076, and 83.060. The *m*/*z* 132.077 is unique to protonated PZNO_2_ and is accompanied by fragment ion signals at *m*/*z* 86.084, 85.076, 57.057, and 44.050; see Section 3.2.2. Likewise, *m*/*z* 116.0824 is unique to protonated PZNO and is accompanied by fragment ion signals at *m*/*z* 86.084, 85.076, and 44.050; see Section 3.2.3. The PZNO photolysis experiments established that the present experiments do not singularize a unique mass peak to protonated PZI (*m*/*z* 85.076), but that *m*/*z* 83.060 (H_2_ ejection from [PZI]H^+^) is characteristic of PZI. Unfortunately, both *m*/*z* 85.076 and 83.060 also have contributions from [PZ]H^+^ amounting to, respectively, 4 and 2% of the total PZ ion signals. Finally, the *m*/*z* 99.092 (C_5_H_11_N_2_^+^) ion signal originates from an unidentified condensation product.

The group (3) signals include *m*/*z* 177.062, 99.055, 81.045, 74.024, 69.045, and 46.029. The *m*/*z* 177.062, unique to PZ(NO_2_)_2_, shows that the primary products undergo further photo-oxidation during the short timespans of the experiments. The *m*/*z* 99.055 (C_4_H_7_N_2_O^+^) is tentatively ascribed to dihydropyrazinone—a possible photo-oxidation product of PZI. The *m*/*z* 81.045 (C_4_H_5_N_2_^+^, protonated pyrazine) signal is puzzling and must have several origins. It clearly correlates with the PZ ion signals before the chamber canopy is opened and with the *m*/*z* 83.060 PZI ion signal after. However, it increases in intensity until the chamber canopy is closed. The peak at *m*/*z* 74.023 is assigned to *N*-formylformamide (CHONHCHO), one of the predicted products following H-abstraction from one of the methylene groups in PZ; the yield was estimated on the basis of the calculated dipole moment and isotropic polarizability (Table S1) to be ∼4%, which agrees with the high-NOx predictions of Scheme 1. Alike the PZNO_2_ photo-oxidation experiments, ion signals at *m*/*z* 46.029 (CH_4_NO^+^) and 69.045 (C_3_H_5_N_2_^+^) grow throughout the PZ photo-oxidation experiments; the former is assigned to protonated formamide/formamidic acid (CHONH_2_/CHOH=NH); the latter is assigned to protonated imidazole.

Figure 6 shows the time evolution of PZ and the photo-oxidation products detected in the gas phase. PZ, PZNO, and PZNO_2_ calibration experiments established the yield of PZNO_2_ to be 6% after 10 min and 7% after 30 min of reaction in the experiment shown. The maximum amount of PZNO is found to be 9% of reacted PZ after 10 min dropping to 1% after 30 min because of photolysis and decreasing NO content during the experiment. Relying on the *m*/*z* 83.060 intensity and including the intensity-corrected *m*/*z* 85.076, the yield of imine was ∼30% after 10 min but only ∼20% after 30 min of reaction.

**Figure 7:**
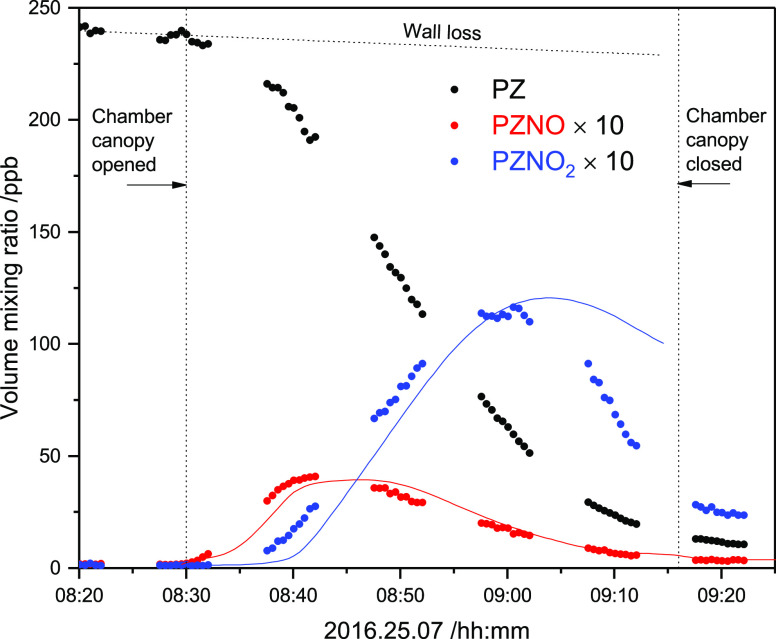
Observed and modeled PZNO_2_ formation in the PZ photo-oxidation experiment on 2016.07.25. The full curves represent the model results for *k*_N–H_/(*k*_N–H_ + *k*_C–H_) = 0.20.

There is a considerable aerosol formation taking place during the experiment, and three of the anticipated products (CHONHCH_2_CH_2_NHCHO, CHONHCH_2_CH_2_N=CH_2_, and CHONHCH_2_OH) that could not be detected in the gas phase with yields >2% were found in the aerosol, see Section 3.2.6. On the other hand, two of the observed gas-phase products (formamide and imidazole), for which there are no obvious gas phase formation routes, can be formed in simple rearrangement reactions of CHONHCH_2_OH, CHONHCH_2_CH_2_NHCHO, and CHONHCH_2_CH_2_N=CH_2_ in the aerosol (see Scheme S2).

#### 3.2.5 N–H/C–H Branching in the Piperazine + OH Reaction

Onel et al.^5^ studied the PZ + OH gas-phase reaction using the pulsed laser photolysis laser-induced fluorescence technique and reported *k*_N–H_/(*k*_N–H_ + *k*_C–H_) = 0.09 ± 0.06 from analysis of OH regeneration in the presence of O_2_/NO.

The present experiments offer an alternative way to obtain the N–H/C–H branching from analysis of the temporal profiles of PZ, PZNO, and PZNO_2_ employing the same chemistry model that was used for PZNO photolysis, Section 3.2.3, only adding a piperazinyl radical source from the reacting PZ. The model takes NO, NO_2_, and *j*_NO2_ from the chamber monitors as input. The OH field and the rate coefficient for wall loss are extracted from the temporal PZ profile, and the wall losses of PZNO and PZNO_2_ are assumed to be the same as that of PZ. There is a very good agreement between the temporal shape of the OH profiles measured directly by FAGE and those derived from the decay of PZ, although there is a significant difference between the absolute concentrations (for more information, see the Supporting Information).

Figure 7 illustrates the results from analysis of the PZ photo-oxidation experiment on 2016.07.25. The PZNO and PZNO_2_ profiles are reproduced reasonably well with *k*_N–H_/(*k*_N–H_ + *k*_C–H_) = 0.2. Six of the seven new PZ photo-oxidation experiments were carried out under conditions that allowed us to extract an average *k*_1a_/(*k*_1a_ + *k*_1b_) = 0.18 ± 0.04 (2σ statistical error) that, although notably larger, agrees with the result of Onel et al.^5^ within the combined error estimates.

**Figure 8:**
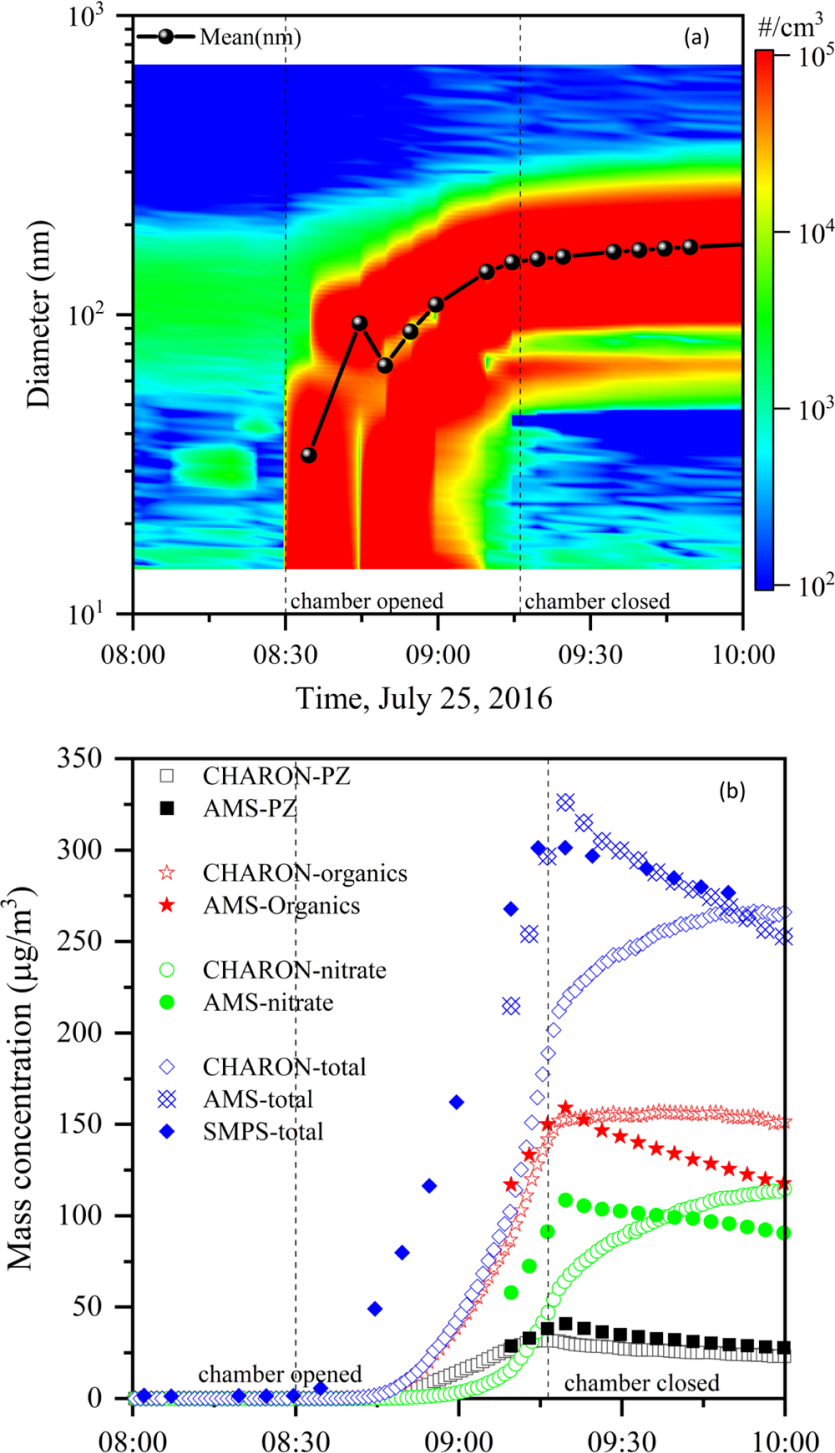
Time evolution of particle size distribution (a) and mass concentrations (b) speciated as PZ, organics, nitrate, and total mass) during the PZ photo-oxidation experiment on July 25, 2016.

#### 3.2.6 Particle Analysis during the Piperazine + OH Reaction

Figure 8 illustrates the results obtained from analyses of particle data collected during PZ photo-oxidation experiments. The top panel shows how the particle size distribution evolved with time. Particles were already present in the chamber before the PZ/NO/IPN mixture was exposed to sunlight. These particles were formed by the reaction of PZ with HNO_3_ (an initial impurity in the NO and later resulting from the NO_2_ reaction with OH). Photo-oxidation of PZ was accompanied by strong particle formation, resulting in a total particle mass loading of ∼300 μg m^–3^ after ∼45 min of solar radiation. At that time, the particle number concentration was 1.4 × 10^5^ cm^–3^ and the mean diameter of the particles was approximately 174 nm. Both AMS and CHARON PTR–ToF–MS measurements (right panel) show that a considerable part of the total aerosol mass was because of piperazinium nitrate (note the delay in time response by the CHARON PTR–ToF–MS instrument), but they clearly also show that the major fraction of the particle mass was composed of organics other than PZ.

**Figure 9:**
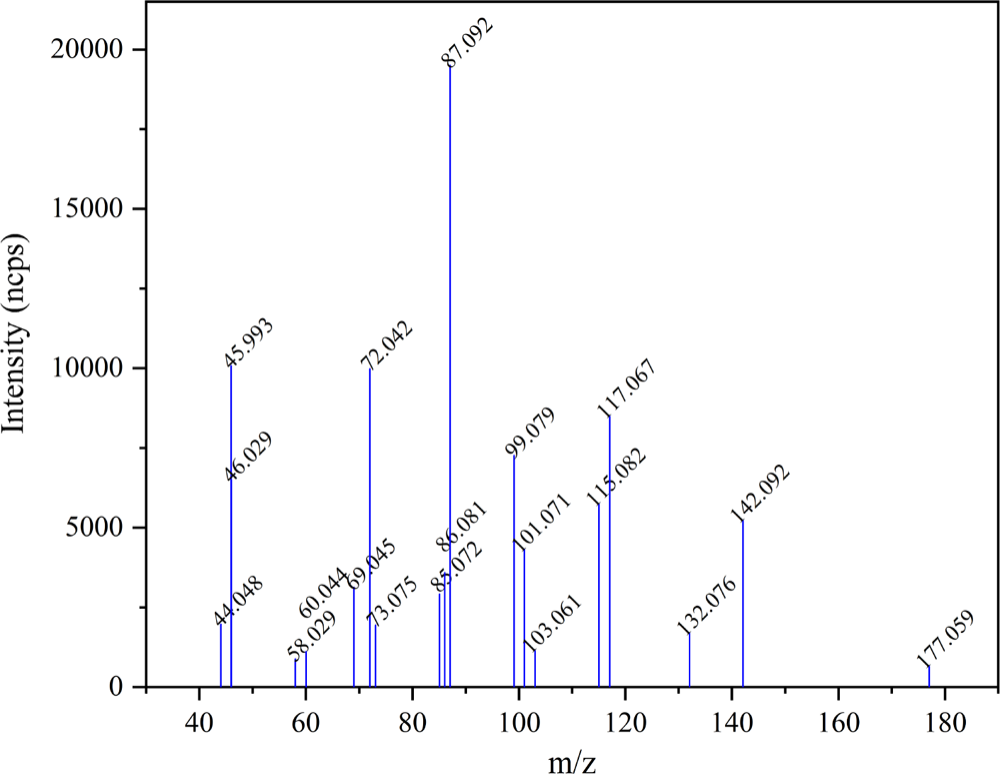
CHARON PTR–ToF–MS mass spectrum obtained from particles formed during 45 min photo-oxidation of a PZ/NO/IPN reaction blend under natural sunlight.

Figure 9 shows the CHARON PTR–ToF–MS mass spectrum collected at 10:00 UTC on 2016.07.25. The most abundant peaks at *m*/*z* 87.092 (C_4_H_11_N_2_^+^) and *m*/*z* 45.993 (NO_2_^+^) are assigned to PZ and nitrate, respectively (nitric acid dehydrates upon protonation in the PTR-MS analyzer). Although most of the aerosol mass peaks observed are also detected in the gas phase (Table 2), there are some important additional ion signals that are assigned to the low volatility products formed upon ring-opening of PZ; see Scheme 1: (1) *m*/*z* 58.029 is assigned to [CHONHCH_2_OH]H^+^ dehydrating in the PTR analyzer; (2) *m*/*z* 101.071 (C_4_H_9_N_2_O^+^) is assigned to the protonated imine, CHONHCH_2_CH_2_N=CH_2_; (3) *m*/*z* 117.067 (C_4_H_9_N_2_O_2_^+^) is assigned to the protonated diamide, CHONHCH_2_CH_2_NHCHO. As already addressed in Section 3.2.4, these three compounds are expected to undergo simple reactions in the aerosol phase to give formamide/formimidic acid and imidazole.

Another important information that can be extracted from the CHARON PTR–ToF–MS mass spectrum is that both the nitramine (PZNO_2_, *m*/*z* 132.076) and the di-nitramine (di-PZNO_2_, *m*/*z* 177.059) were observed in the particle phase. In the exemplified experiment, these two species accounted for 1.7 and 0.9% of the total aerosol mass, respectively. A strong signature of PZNO_2_ was also found in the filter samples analyzed by GC × GC–NCD (see Figure S21 and Table S12 in the Supporting Information). PZNO was not detected in the CHARON PTR–ToF–MS mass spectra, while it was found in trace amounts on the filter samples (Table S12). PZI was not detected in CHARON PTR–ToF–MS mass spectra. Imines are highly reactive compounds and are likely to be rapidly lost in the condensed phase.

## 4 Discussion and Conclusions

To the best of our knowledge, there are only anthropogenic emissions of PZ to the atmosphere. Once in the atmospheric compartment, PZ will partition between the gas phase and the solid/deliquescent particle phase. Kinetic transfer parameters are needed to describe the partitioning, but no such experimental parameters are available for PZ. Assuming that the measured uptake coefficients for methylamines on 59–82 wt % sulfuric acid (γ ∼ 2 × 10^–2^)^42^ establish the level to be expected for amine uptake on deliquescent particles in general, the implication is that the aqueous particle uptake of PZ will be diffusion-controlled under atmospheric conditions. PZ may also form new particles in regions with high levels of acidic compounds. Quantum chemistry calculations of PZ-H_2_SO_4_ clusters suggest that the homogeneous nucleation process may even compete with PZ removal by OH radicals.^43^

The Henry’s law solubility constant for PZ, determined in thermodynamic calculations, is *H*^cp^ = 1.0 × 10^2^ mol m^–3^ Pa^–1^ (the Henry’s law volatility constant *K*_H_ = 1.0 × 10^–2^ m^3^ Pa mol^–1^ = 9.9 × 10^–8^ mol m^–3^ atm^–1^).^44,45^ Under nonreactive equilibrium conditions and assuming the liquid water content in clouds, fog, and urban aerosol to be, respectively, 3, 0.2 and 10^–4^ cm^3^ m^–3^,^46^ PZ will partition roughly 40, 5, and <1% to the aqueous particle phase in the three cases. Nielsen et al.^6^ have estimated the lifetime of PZ with respect to reaction with OH radicals in typical cloud water and deliquescent particles and reported estimated lifetimes of 1 day in the urban cloud, but just 13 min in the deliquescent urban particles. The high reactivity in the deliquescent aerosol will consequently drive additional uptake to the aerosol, and a non-negligible amount of PZ may actually be oxidized there. It should be noted that there are no experimental results from kinetic and mechanistic studies of aqueous phase piperazine reactions, and only speculations on the possible aqueous phase degradation of piperazine have been reported.^47^

With *k*_OH+PZ_ ≈ 2.8 × 10^–10^ cm^3^ molecule^–1^ s^–1^, the lifetime of PZ with respect to gas-phase reaction with OH during daytime will typically be around 1 h. The night-time chemistry of PZ is expected to be dominated by the NO_3_ radical. However, there is no experimental value for *k*_NO_3_+PZ_, but the empirical correlation between OH and NO_3_ rate coefficients for reaction with amines implies a very fast reaction, *k*_NO_3_+PZ_ ≈ 5 × 10^–11^ cm^3^ molecule^–1^ s^–1^ at 298 K.^6^ The average nighttime NO_3_ concentration has been suggested to be around 5 × 10^8^ cm^–3^,^48,49^ which brings the estimated lifetime of PZ during night time to around only a few min. It should be noted that there is no information available in the literature on the branching between N–H and C–H abstraction in amines by NO_3_.

The major product in the atmospheric degradation, PZI, is also expected to react quickly with OH and NO_3_, but also to enter reversible hydrolysis in aqueous particles introducing additional aldehyde and primary amine functionalities: CHOCH_2_NHCH_2_CH_2_NH_2_. Regarding the photo-oxidation products of health concern, PZNO and PZNO_2_, the former will primarily undergo very fast photolysis and only a minor fraction will transfer to the aqueous particle phase (the Henry’s law solubility constant of the dinitrosopiperazine is virtually the same as that of PZ).^50^ PZNO_2_ will undergo relatively fast gas phase photo-oxidation with a few hours’ lifetime with respect to reaction with OH radicals with 1-nitroso-4-nitropiperazine and 1,4-dinitropiperazine among the products. There are no data for the Henry’s law solubility constants for nitramines, but to a first approximation, they are expected to be the same as those of the nitrosamines. Consequently, the major atmospheric degradation of PZNO_2_ is expected to occur in the gas phase.

**Figure 10:**
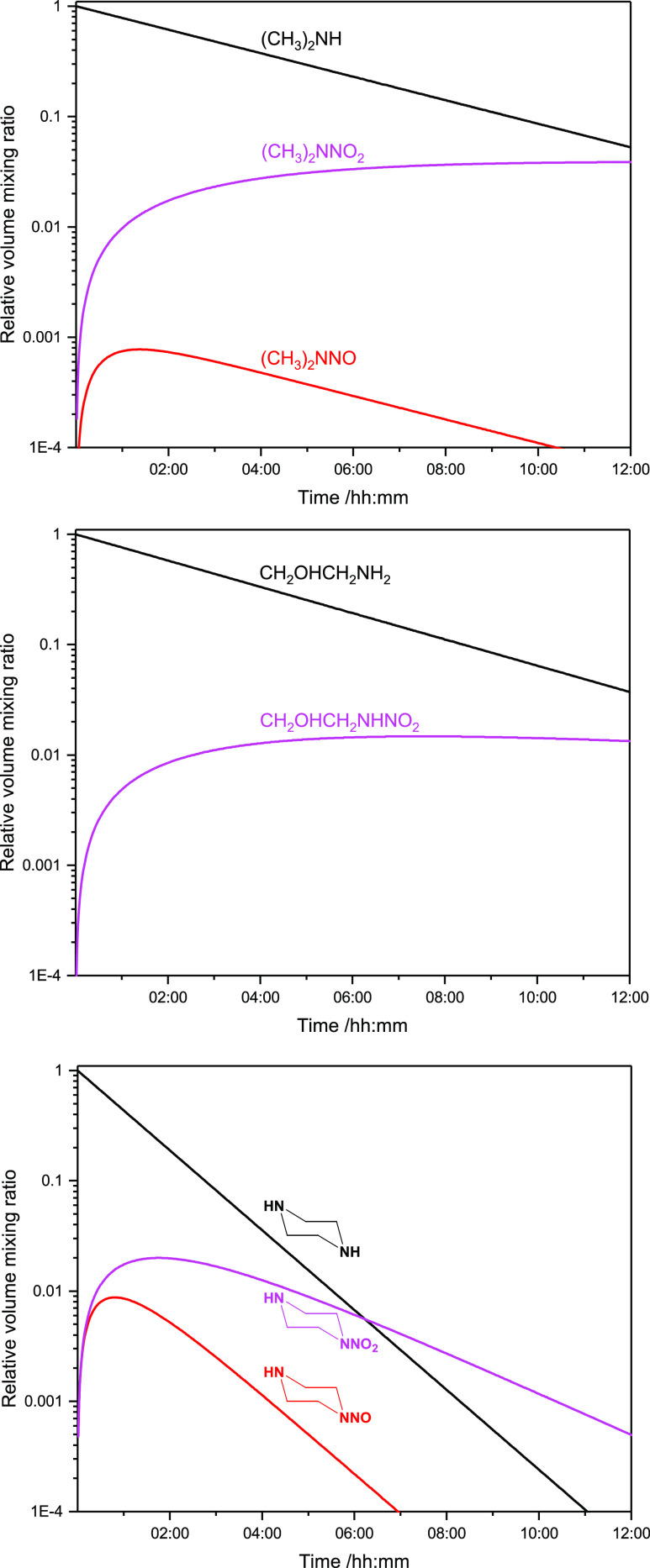
Results from box-modeling the formation of nitrosamines and nitramines in the atmosphere under average conditions in the Oslo region. (top) Dimethylamine, (middle) ethanolamine, and (bottom) piperazine.

The present results permit implementation of a consistent PZ gas-phase degradation mechanism in emission dispersion modeling. A simple box model, based on the atmospheric conditions in the Oslo region, suffices to compare the potential health impact of dimethylamine, ethanolamine (MEA), and PZ emissions from a point source (model parameters in Tables S13, S14). The results, shown in Figure 10, indicate that PZ is the more worrying amine of the three with respect to nitrosamine and nitramine formation per unit of amine emitted. Although the branching between N–H and C–H abstraction in PZ (0.18) is less than half of that of dimethylamine (0.41),^51^ the faster PZ reaction with OH, and the slower PZ aminyl radical reaction with O_2_, more than counterbalances this. Bearing in mind the dilution of an amine injection with distance from emission point, the calculations show that the maximum potential health impact will arise within the first few km from the emission point.

## Supporting Information Available

Details on instrumentation and methodologies including chemical synthesis, atmospheric chemistry of PZ and PZNO_2_ from first principles, PZ + OH kinetics study, PZNO_2_ photo-oxidation study, PZNO photolysis study, PZ photo-oxidation study, and particle analysis (PDF)
