# Supplementary material for: Experimental and Theoretical Study of the OH-Initiated Degradation of Piperazine under Simulated Atmospheric Conditions
Source: J Phys Chem A. 2020 Dec 30;125(1):411–22. doi: 10.1021/acs.jpca.0c10223 (PMC8021224; doi:10.1021/acs.jpca.0c10223)
Supplement: Supplementary file 1 — jp0c10223_si_001.pdf [file jp0c10223_si_001.pdf]

Experimental and Theoretical Study of the OH-Induced Degradation of Piperazine under Simulated Atmospheric Conditions

Supporting Information

Wen Tan,[1§] Liang Zhu,[1§] Tomas Mikoviny,[1] Claus J. Nielsen,[1] Armin Wisthaler,[1] Barbara D´Anna,[2] Simen Antonsen,[3] Yngve Stenstrøm,[3] Naomi J. Farren,[4] Jacqueline F. Hamilton,[4]Graham A. Boustead,[5] Alexander Brennan,[5] Trevor Ingham[5] and Dwayne E. Heard[5]*

[1] Section for Environmental Sciences, Department of Chemistry, University of Oslo, P.O.Box. 1033 Blindern, NO-0315 Oslo, Norway.

[2] Aix Marseille Univ, CNRS, LCE, UMR 7376, 13331, Marseille, France

[3] Faculty of Chemistry, Biotechnology and Food Science, Norwegian University of Life Sciences, P.O. Box 5003, N-1432 Ås, Norway.

[4] Wolfson Atmospheric Chemistry Laboratories, Department of Chemistry, University of York, York, YO10 5DD, United Kingdom.

[5] School of Chemistry, University of Leeds, Leeds, LS2 9JT, United Kingdom.

Content:

Instrumentation and methodologies ... 3
  Table S1. ... 4
Chemicals ... 6
  Acetone cyanohydrin nitrate ... 6
  1-Nitrosopiperazine ... 6
  1-Nitropiperazine ... 6
Atmospheric photo-oxidation of piperazine from first principles. ... 7
  Figure S1. ... 8
  Figure S2. ... 10
  Figure S3. ... 11
  Figure S4. ... 12
  Figure S5. ... 13
  Table S2. ... 14
  Table S3. ... 15
  Table S4. ... 18
  Table S5. ... 22
  Table S6. ... 25
Atmospheric photo-oxidation of 1-nitropiperazine from first principles. ... 27
  Figure S6. ... 28
  Scheme S1. ... 29
  Table S7. ... 30
Piperazine + OH reaction kinetics ... 32
  Figure S7. ... 33
  Figure S8. ... 33
  Figure S9. ... 34
  Figure S10. ... 36
  Figure S11. ... 36
1-Nitropiperazine photo-oxidation studies ... 37
  Table S8. ... 37
  Figure S12. ... 38
1-Nitrosopiperazine photolysis studies ... 39
  Figure S13. ... 39
  Figure S14. ... 40
  Figure S15. ... 40
  Figure S16. ... 41
  Figure S17. ... 41
  Table S9. ... 42
Piperazine photo-oxidation studies ... 43
  Table S10. ... 43
  Figure S18. ... 44
  Figure S19. ... 44
  Scheme S2. ... 45
  Table S11. ... 46
  Figure S20. ... 47
Particle analysis during the piperazine + OH reaction ... 48
  Figure S21. ... 48
  Table S12. ... 48
Implications ... 49
  Scheme S3. ... 49
  Table S13. ... 49
  Table S14. ... 49
References ... 50

Instrumentation and methodologies

European Photoreactor (EUPHORE).

A series of experiments was carried out in chamber B of the EUPHORE facility in Valencia, Spain. The 200 m^3 polytetrafluoroethylene (PTFE) atmosphere simulation chamber has been described in detail elsewhere[1] and only the details pertinent to this work are given here. A syringe pump was used to inject known amounts of PZ dissolved in water (or PZNO or $PZNO_2$) into the chamber via a heated (120 °C) transfer line made of passivated stainless steel. The line was flushed with nitrogen during and after injection. Nitric oxide (NO) was injected using a gas-tight syringe. A basic scrubber was inserted into the injection line for removing trace nitric acid. Nitrogen dioxide (NO_2) was generated from NO via addition of ozone (O_3). After ~1 hour of chamber conditioning the chamber canopy was opened to sunlight radiation. In some experiments isopropyl nitrite (IPN) was continuously added to the chamber in a flow of nitrogen as an efficient OH radical precursor. Acetonitrile was used as a virtually inert dilution tracer in the kinetic study. The EUPHORE facility is equipped with standard monitors for pressure (p), temperature (T), relative humidity, NO, NO_2, O_3, NO_2 photolysis frequency (j_{NO2}) and submicrometer particle size distribution. The latter was measured using a Scanning Mobility Particle Sizer (SMPS) consisting of an electrostatic classifier (Model 3080, impactor: 0.0457 cm) with a long differential mobility analyzer (DMA, Model 3081), and a condensation particle counter (CPC, Model 3022A). For the experiments described herein, we deployed a series of additional analyzers/analytical methods, which are described in more detail below.

Proton-Transfer-Reaction Time-of-Flight Mass Spectrometry (PTR-ToF-MS).

A PTR-TOF 8000 instrument (Ionicon Analytik GmbH, Innsbruck, Austria) was used for measuring PZ and its photochemical oxidation products in the gas phase. The instrument has been described in detail elsewhere[2] and thus only the details pertinent to this study are described here. The drift tube was kept at a temperature of 100 °C and a pressure of 2.30 mbar. The electric field applied to the drift tube was periodically switched in 80 s intervals, i.e. measurements were performed at alternating E/N-values of 65 and 105 Td (1 Td = 10^{-17} V cm^{-2} $molecule^{-1}$), respectively. The PTR-TOF 8000 instrument was interfaced to the chamber using Siltek®/Sulfinert®-treated stainless steel tubing (total length: 143 cm, 60 cm extending into the chamber, ID: 4.57 mm, temperature: 100 °C, flow: 20 lpm). The flow to the instrument was subsampled through PEEK® (polyetheretherketone) capillary tubing (OD: 1.59 mm). The instrument was regularly calibrated against a gaseous reference standard containing 13 hydrocarbons and oxygenated hydrocarbons. The instrument was further calibrated for piperazine and 1-nitro-piperazine using a commercial liquid calibration unit (LCU; Ionicon Analytik GmbH, Innsbruck, Austria) for evaporation of gravimetrically prepared aqueous standards in nitrogen. The fragmentation pattern of 1-nitroso-piperazine was taken from measurement of 1-nitroso-piperazine in the chamber prior to canopy opening. The calibration factor of 1-nitroso-piperazine was further derived relying on experimental mass discrimination function and calculated reaction rate coefficient. For other analytes reported herein, we used averages of the theoretically derived instrumental response factors summarized in Table S1.

Chemical Analysis of Aerosol Online (CHARON)

A prototype CHARON inlet[3,4] was interfaced to a second PTR-TOF 8000 instrument (Ionicon Analytik GmbH, Innsbruck, Austria) for measuring particle chemical composition. The CHARON inlet strips off gas-phase analytes, enriches the particle concentration in the PTR-ToF-MS subsampling flow and vaporizes the particles prior to ionization and mass spectrometric analysis. In this study, the vaporization temperature was set to 140 °C. The CHARON inlet was interfaced to the EUPHORE chamber using Siltek®/Sulfinert®-treated stainless steel tubing (total length: 415 cm, 40 cm extending into the chamber, ID: 4.57 mm). The PTR-ToF-MS drift tube was kept at a voltage of 350 V, a temperature of 130 °C and a pressure of 2.40 mbar (100 Td). The instrument was regularly calibrated against a gaseous reference standard containing 13 pure and oxygenated hydrocarbons. A reference mass spectrum was obtained from the PZ-aminium nitrate salt.

Table S1.

Dipole moments (μ /Debye), isotropic polarisabilities (α /A^3)

Formula	QCC[a]	μ	α	k[b] /10^{-9} cm^3 molecule^{-1} s^{-1}		
				E/N = 65	E/N = 85	E/N = 105
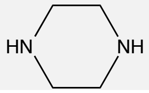	B3L	0	10.022	1.871	1.871	1.871
	M06	0	9.619	1.833	1.833	1.833
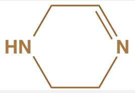	B3L	2.595	9.386	3.303	3.210	3.092
	M06	2.625	9.189	3.308	3.215	3.100
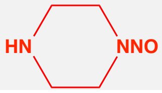	B3L	4.599	11.831	4.823	4.614	4.442
	M06	4.533	11.397	4.748	4.541	4.372
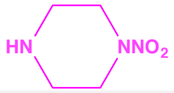	B3L	4.516	12.292	4.738	4.540	4.376
	M06	4.204	11.712	4.473	4.296	4.147
$CHONHCH_2CH_2N{=}CH_2$	B3L	4.516	12.292	4.850	4.631	4.452
	M06	4.204	11.712	4.871	4.645	4.459
$CHONHCH_2CH_2NHCHO$	B3L	5.567	11.133	5.524	5.237	4.998
	M06	5.065	10.687	5.108	4.856	4.648
CHONHCHO	B3L	2.791	6.220	3.230	3.118	3.020
	M06	2.896	5.959	3.289	3.167	3.063
$CHONHCH_2OH$	B3L	3.098	6.220	3.486	3.353	3.240
	M06	3.299	5.959	3.620	3.468	3.342
$(CH_3)_2CO$	B3L	3.079	6.34	3.646	3.507	3.390
	M06	3.100	6.11	3.644	3.501	3.381

[a] B3L, B3LYP/aug-cc-pVTZ; M06, M06-2X/aug-cc-pVTZ. [b] Collisional rate coefficients at 100 °C (H_3O^+ - analyte molecule) used for determining the PTR-ToF-MS response factors.

Aerosol Mass Spectrometry

A compact time-of-flight Aerosol Mass Spectrometer (C-ToF-AMS, Aerodyne Research Inc., Billerica, MA, U.S.A.) [5] was used for measuring total particle mass loading and particle chemical composition. A reference spectrum was obtained from the piperazinium nitrate salt. The cToF-AMS data set was processed using a cumulative peak fitting analysis which allows to separate multiple isobaric peaks that are not taken into account in the traditional analysis of unit mass resolution data. [6] Uncertainties in the major chemical species from the cToF-AMS are typically of the order of ±30%. [5]

Aerosol filter sample collection, extractionand analysis

Aerosol formed in the chamber was collected onto pre-baked (550 °C, 6 h) 47 mm diameter quartz microfibre filters (Whatman, Maidstone, U.K.) at regular intervals throughout each photo-oxidation experiment. Two sets of filters were collected in parallel by sampling at 10 L min^{-1} and splitting the air flow equally between two 47 mm filter holders. The filter collection times ranged from 30 to 60 min depending on the mass of aerosol present in the chamber. ¼" PFA tubing was used for all connections between the chamber, filters and sampling pump. At the end of each experiment when the chamber canopy had been closed, an additional filter was collected using a high-volume sampling pump (flow rate: 48 L min^{-1}, sampling time: 60 min, total volume collected: 2.88 m^3). After collection the filter samples were stored at -18 °C until analysis. One set of filter samples were extracted into ethyl acetate (EtOAc) using an accelerated solvent extraction (ASE) system, prior to being evaporated to 1 mL under a gentle stream of nitrogen. Full details of the ASE method can be found elsewhere. [7] This set of samples were analysed using two-dimensional gas chromatography – nitrogen chemiluminescence detection (GC×GC-NCD) to detect nitrosamines and nitramines in the aerosol.

Chromatographic analysis was carried out on a GC×GC-NCD system comprised of an Agilent 7890 gas chromatograph and an Agilent 255 NCD system (Palo Alto, CA, United States). The first column was a nonpolar Ultra Inert DB5 (30 m × 0.32 mm i.d. × 0.25 µm film thickness) and the second column a midpolarity DB-17 (3 m × 0.10 m i.d. × 0.10 µm film thickness). Both columns were purchased from Agilent Technologies Ltd., Stockport, U.K. The initial temperature of the first dimension column was 40 °C for 2 min, followed by a heating rate of 7 °C min^{-1} to 100 °C for 8 min and then further heating at 7 °C min^{-1} until 270 °C was reached and held isothermally for a further 5 min. A temperature offset of 30 °C was applied to the second dimension column throughout the GC temperature program. A liquid nitrogen two-stage cold jet modulation system was used, with a modulation period of 5 s and a +15 °C offset from the secondary GC oven temperature. Data was collected at 200 Hz over the entire course of the analysis, and hydrogen was used as a carrier gas at 1.4 mL min^{-1}. Injections of 1 µL were performed in splitless mode at an injection temperature of 200 °C using an automated liquid injector (Gerstel, Mülheim an der Ruhr, Germany). Pyrolysis of the analytes in the dual plasma burner was carried out at 900 °C under a hydrogen flow rate of 4 mL min^{-1} and an oxygen flow rate of 10 mL min^{-1}. *N*-nitropiperazine was synthesized at the Norwegian University of Life Sciences in Oslo, Norway and *N*-nitrosopiperazine and 1,4-dinitrosopiperazine were available as solids from Santa Cruz Biotechnology (U.S.). *N*-nitrosopiperazine and *N*-nitropiperazine were derivatised prior to analysis, using acetyl chloride and triethylamine (a full description of the derivatisation process can be found elsewhere, manuscript in preparation). Nitrosamine and nitramine recoveries were calculated by comparing the peak areas obtained from spiked filters with peak areas acquired from standard directly injected to the GC×GC-NCD. The reported recoveries include the derivatisation step for *N*-nitrosopiperazine and *N*-nitropiperazine. The recovery tests were performed in triplicate and average recovery levels of 82.1% (RSD = 11%), 91.6% (RSD = 11.4%) and 89.3% (RSD = 0.9%) were achieved for *N*-nitrosopiperazine, *N*-nitropiperazine and 1,4-dinitrosopiperazine respectively.

Fluorescence Assay by Gas Expansion (FAGE)

A FAGE (Fluorescence Assay by Gas Expansion) apparatus was used for the measurement of OH radicals during the 2016 ACA campaign. FAGE is a method based on laser-induced fluorescence (LIF) spectroscopy at low pressure, and has been widely used for the measurement of OH concentrations both in the field [8] and in the laboratory. The OH radical is excited with a wavelength-tunable pulsed laser at 308 nm (via the $A^2\Sigma\ v'=0 \leftarrow X^2\ \Pi_i\ v''=0$ electronic transition), after which on-resonance fluorescence around 308 nm is detected.[9] FAGE uses a low pressure fluorescence detection cell (pressure held at ~2 Torr or less) to extend the fluorescence lifetime of OH to beyond that of the excitation laser pulse (~10 ns), and gated photon counting is then used to separate the small fluorescence signal (which is detected) from the much larger signal from laser-scattered light [9].

The laser and detection cells used in the ACA campaign both follow the same design as those used in the Leeds aircraft FAGE instrument which is discussed in detail in Commane et al., 2010.[10] The 308 nm laser light was generated by tripling the 924 nm output from a Nd:YAG pumped Ti:Sapphire laser (Photonics Industries Inc). The 308 nm light was generated at a pulse repetition frequency of 5000 Hz and with a typical laser power of 5-15 mW (corresponding to 1-3 µJ per pulse) and delivered to the fluorescence cell via a fibre optic cable (Oz optics). The fluorescence signal was detected using a multi-channel plate (MCP) detector (PMT325/Q/BI/G, Photek Ltd.) equipped with a gating unit (GM10-50, Photek) and fast pre-amplifier (PA200-10, Photek), with the output sent to a PMS-400A photon counting card (Becker & Hickl GmbH). The photon counts are integrated over a one second period, which consists of 5000 excitation laser pulses.

As the FAGE technique does not provide an absolute measurement, a calibration is required in order to relate the fluorescence signal to a concentration. The calibration of the instrument for OH utilises a turbulent reactor [10] which produces OH following the photolysis of water vapour at 185 nm using a mercury pen-lamp. With knowledge of the concentration of water vapour (determined using a chilled mirror hygrometer), the flux of the lamp at 185 nm and the photolysis time (the product of which is determined using a N_2O chemical actinometer), and the absorption cross-section of water vapour and the OH quantum yield at 185 nm, the OH concentration can be calculated.[10] By changing the flux of the radiation from the pen-lamp at 185 nm, a range of known OH concentrations could be generated and

were used to calibrate the fluorescence signal. From the calibration a limit of detection for OH of ~ 1 x 10^6 molecule cm^{-3} was derived for a signal-to-noise ratio of 1 and an averaging time of 2 minutes.

Chemicals

PZ (Sigma-Aldrich, ReagentPlus®, 99%), acetonitrile (Sigma-Aldrich, LiChrosolv®, ≥99.8%), pyrrole (Sigma-Aldrich, 98 %), 1,3,5-trimethylbenzene (Sigma-Aldrich, 98 %), isoprene (Sigma-Aldrich, analytical standard) and limonene (R-(+), Sigma-Aldrich, analytical standard) were used without further purification. 2-propyl nitrite (isopropyl nitrite, IPN) was synthesized from isopropanol, hydrochloric acid and sodium nitrite, and purified by repeated washing with ice water. The 1:1 nitric acid salt of PZ was prepared by adding a small excess of diluted nitric acid (HNO_3) to diluted PZ followed by rotary evaporation to dryness at 80 °C.

Acetone cyanohydrin nitrate

Acetone cyanohydrin nitrate, $NCC(CH_3)_2ONO_2$, was synthesized according to a previously published procedure,[11] but with some modifications due to the hazard of nitrate compounds. To a stirred solution of white fuming nitric acid (d. 1.48-1.50; 231 g; 3.67 mol) in acetic anhydride (612.0 g; 6.0 mol) was added acetone cyanohydrin[2] (127.5 g; 1.5 mol) in one portion. The solution was stirred at room temperature for 30 minutes and then poured into 750 mL of ice-water. After one hour of intermittent stirring, the aqueous solution was extracted with methylene chloride (3 x 150 mL). The combined methylene chloride solution was washed once with 200 mL of 5% $NaHCO_3$ (aq) and then dried ($MgSO_4$). Fractional distillation at reduced pressure yielded 131.1 g (66%) acetone cyanohydrin nitrate, b.p. 70-72°C/12 mmHg. The compound was stored cold (refrigerator) and used directly in the piperazine-mono-nitramine synthesis.

Safety precautions in the syntheses of 1-nitrosopiperazine and piperazine-mono-nitramine

All reactions have been done in an inert atmosphere (N_2) in a well-ventilated fume hood. Usual precautions have been implemented when working with these compounds, i.e. use of gloves, laboratory coat and safety goggles. Those compounds specially suspected to be cancer promoting or explosive is treated wet and destroyed according to standard laboratory practice. All chemical waste are sealed in special flasks and sent to authorised companies for destruction of such materials.

1-Nitrosopiperazine

1-Nitrosopiperazine; $HN<^{CH_2CH_2}_{CH_2CH_2}>NNO$. A slight modification of a previously published procedure was used:[12] Piperazine (1.72 g, 20 mmol) in 6M HCl (12 mL) was cooled to -10 °C and a solution of $NaNO_2$ (1.39 g, 20 mmol) in water (24 mL) was added slowly over 1 h. The temperature was kept below 0 °C when pH was adjusted to ≈10 (pH-paper) using 2M NaOH. The mixture was extracted with chloroform, dried ($MgSO_4$), and the solvent removed by evaporation. The crude product was purified by column chromatography using silica gel and eluted with 8% MeOH/CH_2Cl_2. A yellow oil resulted. Yield: 1.70 g (74%) ^{1}H NMR (300 MHz, $CDCl_3$) δ: 4.15-4.18 (m, 2H), 3.74-3.77 (m, 2H), 3.00-3.03 (m, 2H), 2.75-2.79 (m, 2H), 1.83 (s, 1H).

1-Nitropiperazine

Piperazine-mono-nitramine; $HN<^{CH_2CH_2}_{CH_2CH_2}>NNO_2$. The published procedure[11] has been modified due to the potential hazard working with these compounds. A three-necked flask was charged with DMF (100 mL) and piperazine hexahydrate (38.41g; 0.20 mol). Acetone cyanohydrin nitrate (13.0 g, 0.10 mol; synthesis described above) was added dropwise and the reaction was heated to 80 °C for 5 h. The bulk of the solvent (60-70%) was evaporated in vacuo by distillation using a Vigreux column. The residue was cooled in a freezer at ≈ -20 °C overnight to ensure slow crystallization, then the flask was cooled to -72 °C (dry-ice/ethanol) and the precipitated pale, yellow crystals were collected by filtration. Recrystallization from ethanol (95%) gave 7.9 g (60%) of white crystals. Mp. 127-128 °C (lit.[13] mp.

127-128 °C). ^{1}H NMR (300 MHz, $CDCl_3$): δ: 1.54 (bs, 1H), 2.96 (m, 4H), 3.79 (m, 4H) ppm; ^{13}C NMR (75 MHz, $CDCl_3$): δ: 44.70, 49.67 ppm.

Atmospheric photo-oxidation of piperazine from first principles.

The initial step in the OH reaction with piperazine is either N-H or C-H abstraction:

$\Delta H^{\ominus}$ = -103 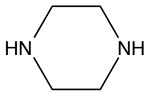 + OH → 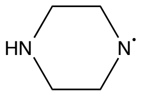 + H_2O (1a)

$\Delta H^{\ominus}$ = -111 → 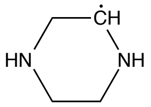 + H_2O (1b)

The reaction enthalpies given with the equations ($\Delta H^{\ominus}$ /kJ mol^{-1}) stem from G4 calculations and always refer to the lowest energy conformations of the species involved.

Atmospheric fate of the $HN\langle^{CH_2CH_2}_{CH_2CH_2}\rangle\dot{N}$ radical

The $HN\langle^{CH_2CH_2}_{CH_2CH_2}\rangle\dot{N}$ radical is expected to react with O_2, NO, NO_2 and O_3. Lazarou *et al.* reported the rate coefficients for the $(CH_3)_2\dot{N}$ radical reactions with NO and NO_2 at low pressure to be respectively $(8.5 \pm 1.4) \times 10^{-14}$ and $(9.1 \pm 1.4) \times 10^{-13}$ cm^3 molecule^{-1} s^{-1} at 298 K.[14] Lindley *et al.* reported the $(CH_3)_2\dot{N}$ radical reaction with O_2 to be 3.9×10^{-7} times slower than the corresponding NO_2 reaction.[15] There are no kinetic data on the reaction between O_3 and alkylamino radicals. The $\dot{N}H_2$ radical is reported to react with O_3 with a rate coefficient of $k = 1.7 \times 10^{-13}$ cm^3 molecule^{-1} s^{-1} (extensive review[16]). Peiró–García et al. characterized the $\dot{N}H_2 + O_3$ reaction in MP2 and CCSD(T) calculations, and found the reaction to proceed via $H_2N\dot{O}$ to HNO.[17-18] A comparison of barriers obtained in CCSD(T)/6-311G(3df,2p)//MP2/6-311+G(d,p) calculations on the $\dot{N}H_2 + O_3$ and $CH_3\dot{N}H + O_3$ reaction systems indicates that the methylamino radical will react around 3 times faster with O_3 than the $\dot{N}H_2$ radical does, i.e. with a rate coefficient ~5×10^{-13} cm^3 molecule^{-1} s^{-1}; it is expected that the $HN\langle^{CH_2CH_2}_{CH_2CH_2}\rangle\dot{N}$ radical will react in a similar manner and with a similar rate coefficient. The total bimolecular loss rate of the $HN\langle^{CH_2CH_2}_{CH_2CH_2}\rangle\dot{N}$ radical at semi-rural atmospheric conditions (20 % O_2, 6 ppb NO_2, 2 ppb NO, 40 ppb O_3) is therefore expected to be in the order of 1 s^{-1}; reaction with O_2 is estimated to account for around 50 % of the $HN\langle^{CH_2CH_2}_{CH_2CH_2}\rangle\dot{N}$ radical loss, O_3 for around 40 %, NO_2 for around 10 %, and NO for <1 %. Quantum chemistry calculations show that the $>\dot{N} + O_2$ reaction proceeds via the $>NO\dot{O}$ (piperazinyldioxy) radical on the entrance side and a post reaction HO_2 complex on the exit side, and that the barrier is around 5 kJ mol^{-1} higher than in the corresponding $(CH_3)_2\dot{N} + O_2$ reaction, see Table S2. This suggests that the piperazinyl + O_2 reaction is an order of magnitude slower than the $(CH_3)_2\dot{N} + O_2$ reaction.

$\Delta H^{\ominus}$ = -103 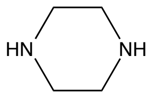 + OH → 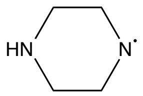 + H_2O (1a)

$\Delta H^{\ominus}$ = -66 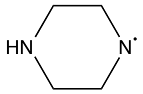 + O_2 → 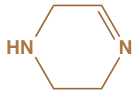 + HO_2 (2)

The $HN<^{CH_2CH_2}_{CH_2CH_2}>\dot{N}$ radical reaction with NO will result in nitrosamine formation, whereas the reaction with NO_2 in principle can lead to the imine, the nitramine and the nitrite:

$\Delta H^{\Theta} = -185$ (3)

$\Delta H^{\Theta} = -184$ (4a)

$\Delta H^{\Theta} = -192$ (4b)

$\Delta H^{\Theta} = -115$ (4c)

$\Delta H^{\Theta} = +39$ (5)

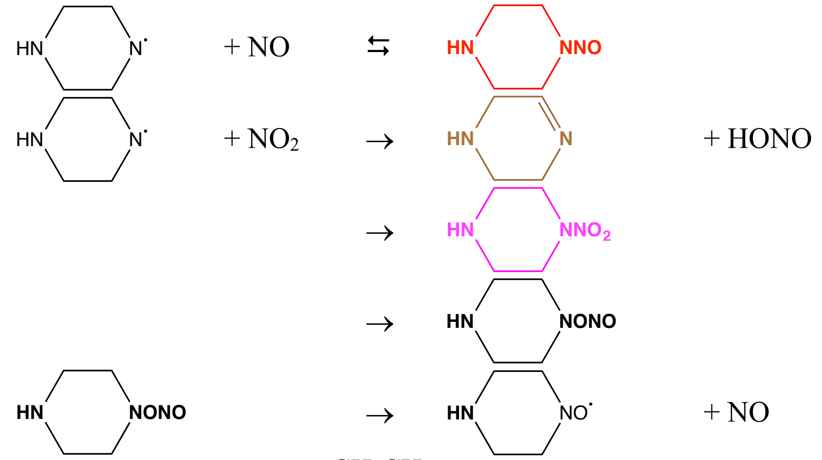

Figure S1 illustrates the PES for the $HN<^{CH_2CH_2}_{CH_2CH_2}>\dot{N} + NO_2$ reaction; the underlying quantum chemistry data are collected in Table S3. An inspection of the PES illustrated in Figure S1 shows that reaction (4a) is of no importance under atmospheric conditions. There is apparently no additional electronic barrier to the nitrite dissociation, reaction (5), and the nitrite will therefore undergo spontaneous dissociation.

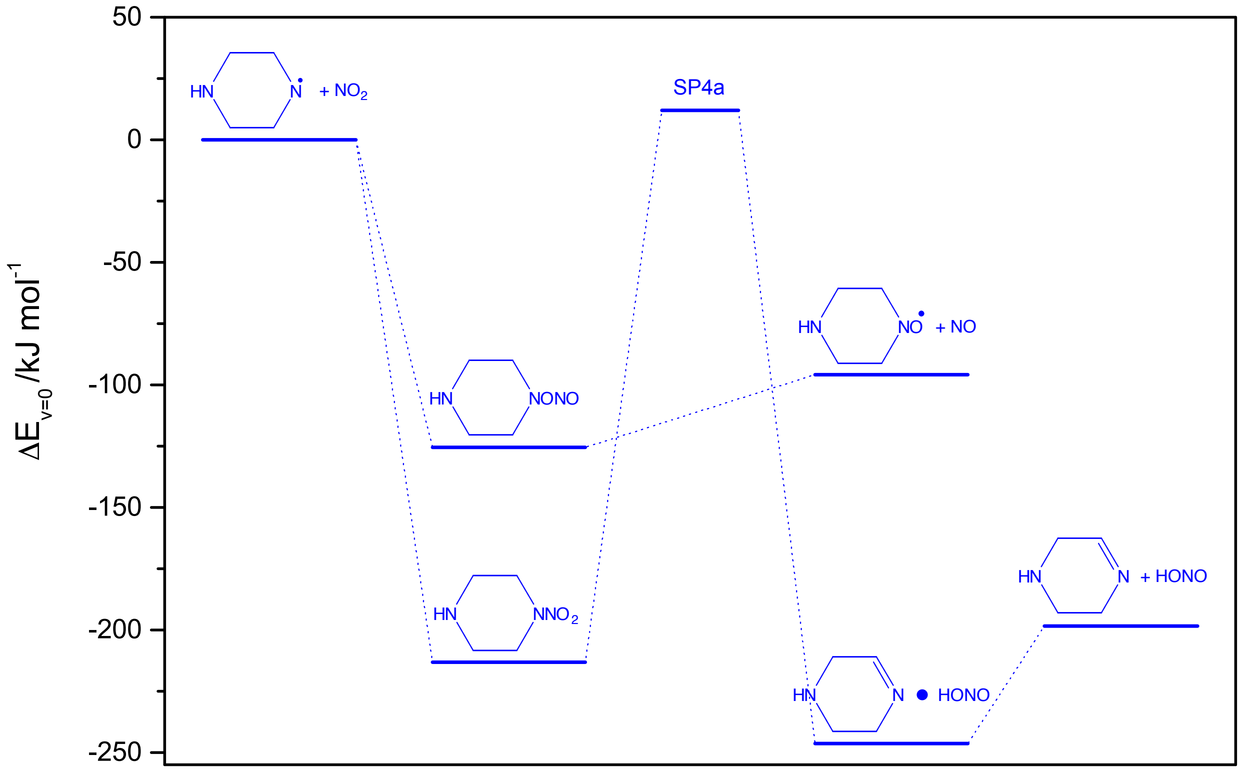

Figure S1.

Stationary points on the PES for the $HN<^{CH_2CH_2}_{CH_2CH_2}>\dot{N} + NO_2$ reaction. Results from M06-2X/aug-cc-pVTZ calculations.

The $HN<^{CH_2CH_2}_{CH_2CH_2}>\dot{N}$ radical reaction with O_3, mentioned above, leads to the piperazinyloxy radical:

$\Delta H^{\ominus} = -280$ 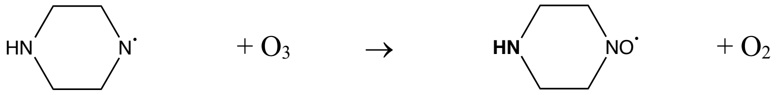 (6)

The nitroxide radical formed in (4c) and (6) may in principle dissociate before reacting with O_3. In a worst case scenario with respect to nitrosamine and nitramine formation, however, the $HN<^{CH_2CH_2}_{CH_2CH_2}>\dot{N}$ radical is just recycled in reactions (4c), (6) and (7).

$\Delta H^{\ominus} = -11$ 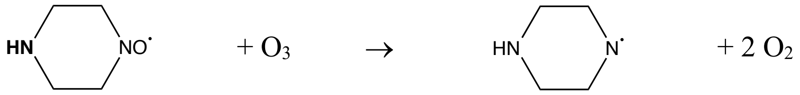 (7)

In summary, the quantum chemistry calculations predict the atmospheric fate of the $HN<^{CH_2CH_2}_{CH_2CH_2}>\dot{N}$ to be as shown below. Note that the piperazinyl and piparzinyloxo radicals are in steady state, and that the steady state approximation can be applied for modelling purposes.

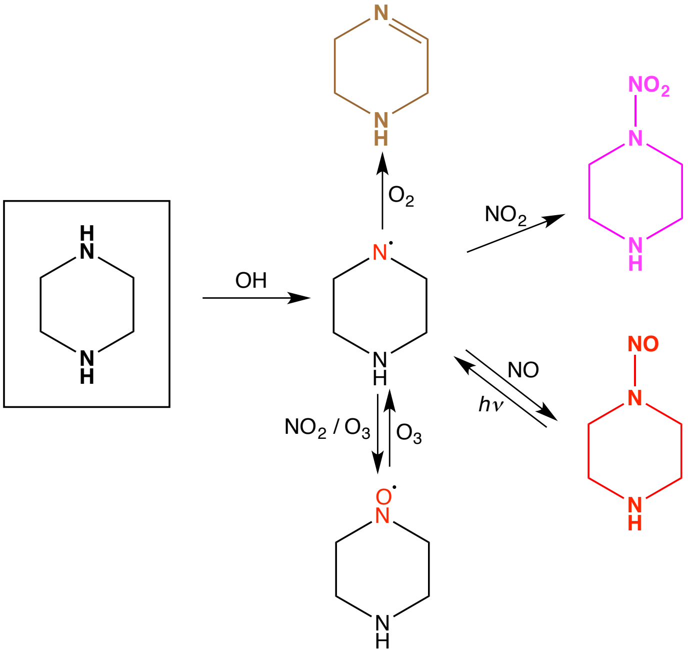

Atmospheric fate of the $HN<^{CH_2\dot{C}H}_{CH_2CH_2}>NH$ radical

The alkyl radical formed in (1b) will add O_2 forming a vibrationally excited peroxy radical that may initiate several internal H-transfer reactions before being quenched by collisions and reaction with NO. Note that there are two different isomers formed of the peroxy radicals, (*eq* or *ax*) and that the barriers to the possible internal H-transfer reactions will be different for these two isomers.

$\Delta H^{\ominus} = -111$ 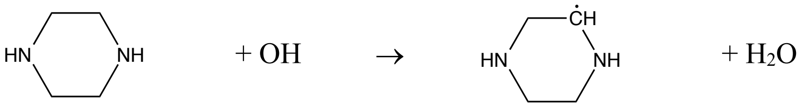 (1b)

$\Delta H^{\ominus} = -147$ 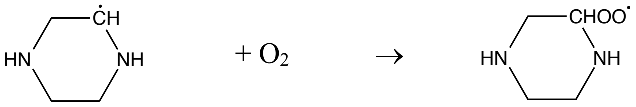 (8)

$\Delta H^{\ominus} = +88$ 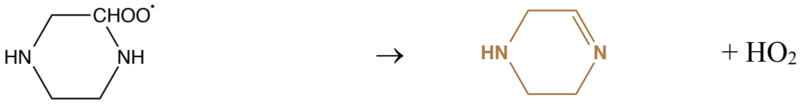 (9a)

$\Delta H^{\Theta} = +89$ → 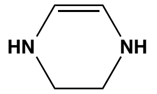 $+ HO_2$ (9b)

$\Delta H^{\Theta} = +37$ → 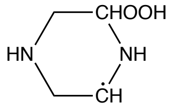 (9c)

$\Delta H^{\Theta} = -40$ 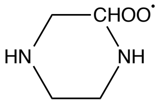 $+ NO$ → 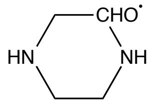 $+ NO_2$ (10)

Figure S2 illustrates the relative energies of the stationary points on the PES of the $HN<^{CH_2\dot{C}H}_{CH_2CH_2}>NH + O_2$ reaction; the underlying quantum chemistry data are collected in Table S3. The barriers to the internal H-transfer reactions are all calculated to be well below the entrance energy of reactants. However, the barrier to imine formation, reaction (9a), is significantly lower than those of (9b) and (9c), and the latter two reactions can be disregarded at atmospheric conditions. The competition between (9a) and (10) was investigated in master equation calculations based on the PES shown in Figure S2. Assuming a collisional quenching of $<\Delta E_{down}> = 250$ cm^{-1}, a typical rate coefficient of 4×10^{-12} cm^3 molecule^{-1} s^{-1} for reaction (10), and a mixing ratio of 5 ppb for NO leads to around 75 % yield of the oxy-radical. For $<\Delta E_{down}> = 200$ cm^{-1} the oxy-radical yield is reduced to 67%, and modifying the barrier height by ±4 kJ mol^{-1} in addition changes the yield to 80 and 40%, respectively. Increasing the NO mixing ratio to 50 ppb has little impact on the branching. It is concluded that the theoretical calculations can only place the oxy-radical yield to be in the range 40 – 80 %.

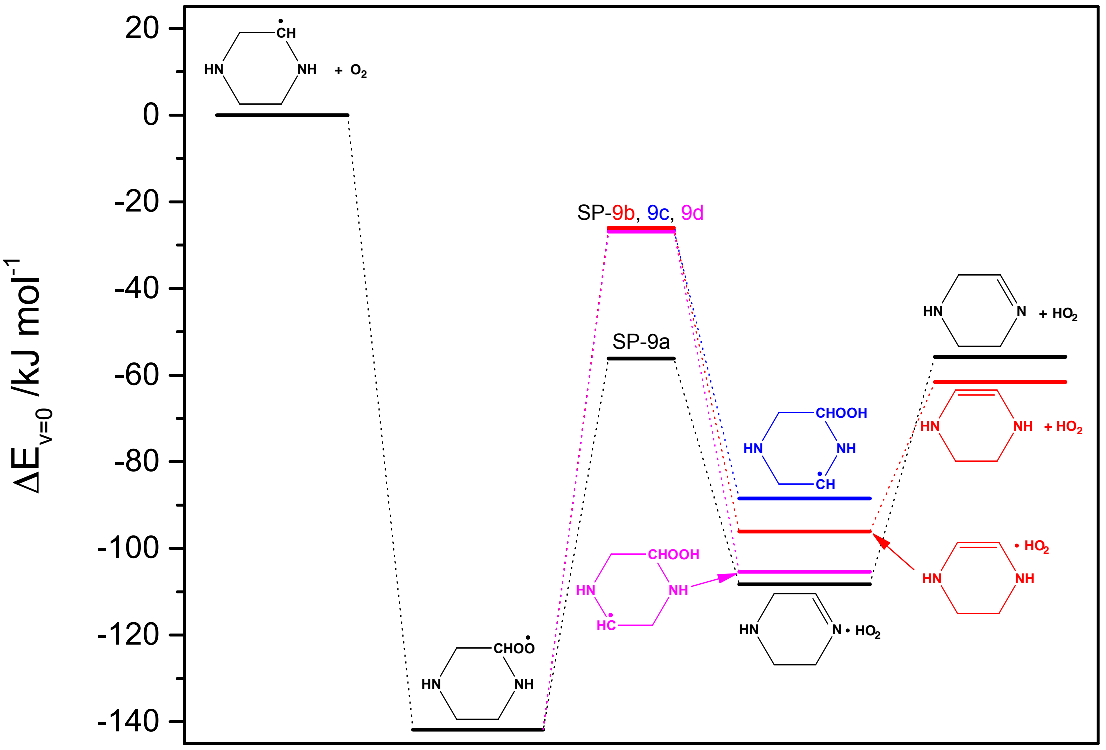

Figure S2.

Stationary points on the PES for $HN<^{CH_2\dot{C}H}_{CH_2CH_2}>NH + O_2$ reaction. Results from M06-2X/aug-cc-pVTZ calculations.

The oxy radical may in principle dissociate (ring opening), initiate internal H-transfer reactions or react with O_2 forming an amide moiety. It turns out, however, that the oxy radical is metastable with a very low barrier to C–C bond scission illustrated in Figure S3. The oxy radical will therefore undergo spontaneous ring opening:

$\Delta H^{\Theta} = -84$ 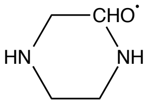

$\xrightarrow{\Delta}$ $CHONHCH_2CH_2NH\dot{C}H_2$ (11)

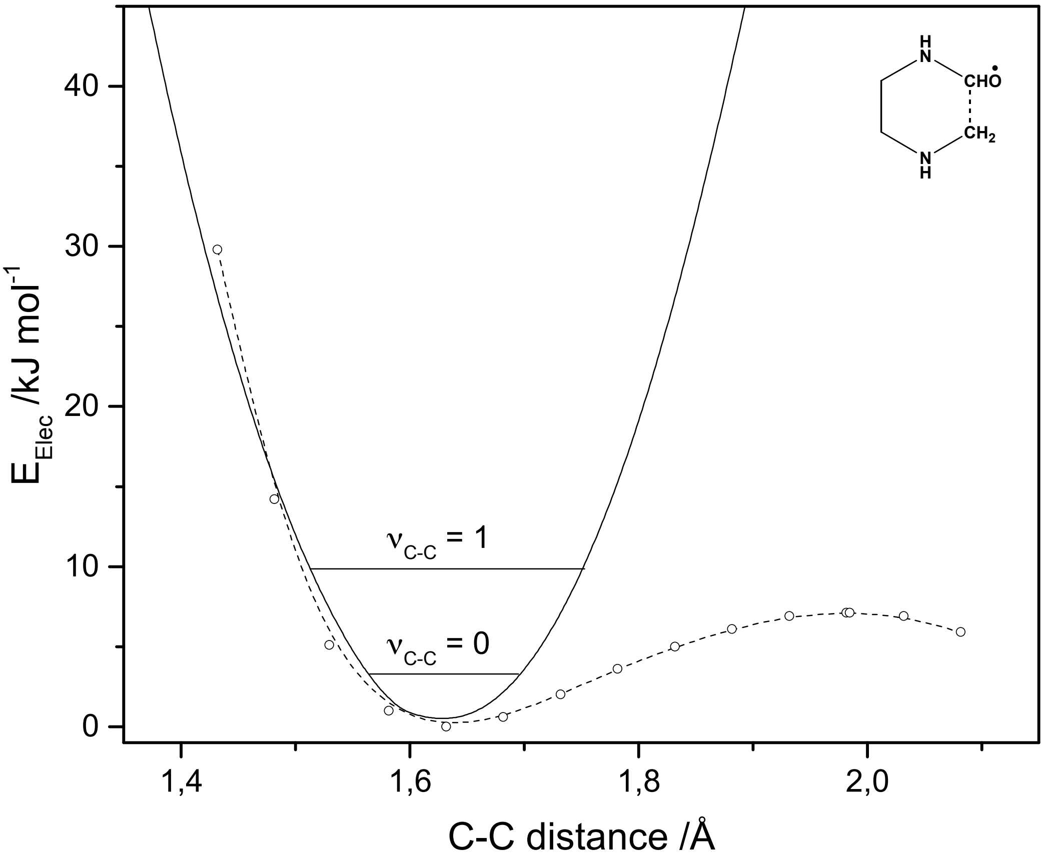

Figure S3.
Potential energy of the $NHCH_2CH_2NHCH_2CH\dot{O}$ radical as function of the $(HNH_2)C$—$C(H(\dot{O})NH)$ distance. ○, results from M06-2X/6-31+G(d,p) calculations. Full curve: The harmonic oscillator potential of the C-C stretching mode in the $NHCH_2CH_2NHCH_2CH\dot{O}$ radical.

The alkyl radical formed in (11) will add O_2 to give an an energized peroxy radical that may initiate internal H-transfer reactions (imine formation) before being quenched by collisions and subsequent reaction with NO to the corresponding oxy radical:

$\Delta H^{\Theta} = -137$ $CHONHCH_2CH_2NH\dot{C}H_2 + O_2 \rightarrow CHONHCH_2CH_2NHCH_2O\dot{O}^{\ddagger}$ (12)

$\Delta H^{\Theta} = -56$ $CHONHCH_2CH_2NHCH_2O\dot{O}^{\ddagger} \rightarrow CHONHCH_2CH_2N{=}CH_2 + HO_2$ (13)

$\Delta H^{\Theta} = -49$ $CHONHCH_2CH_2NHCH_2O\dot{O} + NO \rightarrow CHONHCH_2CH_2NHCH_2\dot{O} + NO_2$ (14)

The branching between reactions (13) and (14) was estimated in master equation calculations based on the PES illustrated in Figure S4 (the underlying quantum chemistry data are collected in Table S5),

and a collisional quenching of <ΔE_{down}> = 250 cm^{-1}, a typical rate coefficient of 4 × 10^{-12} cm^3 molecule^{-1} s^{-1} for reaction (14), and a mixing ratio of 2 ppb for NO. The calculations show that the branching is virtually independent of <ΔE_{down}> and the NO mixing ratio implying a 98 % yield of the oxy-radical. Lowering the barrier to reaction (13) by 10 kJ mol^{-1} decreases the oxy-radical yield to 87 %. A conservative estimate of the (13) : (14) branching is therefore <10% : >90 %.

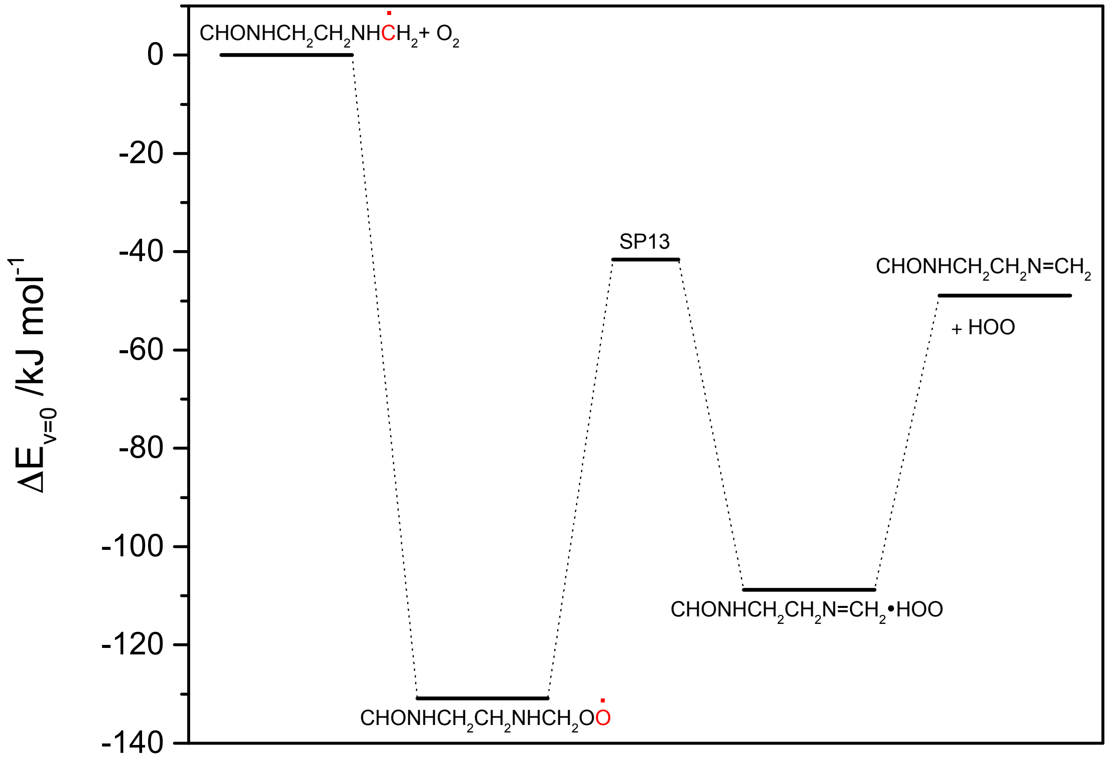

Figure S4.
Stationary points on the PES for the $CHONHCH_2CH_2NH\dot{C}H_2 + O_2$ reaction. Results from M06-2X/aug-cc-pVTZ calculations.

The oxy-radical formed in (14) may dissociate, undergo H-abstraction by O_2 or initiate internal H-transfer reactions:

$\Delta H^{\ominus} = +49$	$CHONHCH_2CH_2NHCH_2\dot{O}$	$\xrightarrow{\Delta}$	$CHONHCH_2CH_2\dot{N}H + CH_2O$	(15)
$\Delta H^{\ominus} = -201$	$CHONHCH_2CH_2NHCH_2\dot{O} + O_2$	$\rightarrow$	$CHONHCH_2CH_2NHCHO + HO_2$	(16)
$\Delta H^{\ominus} = -29$	$CHONHCH_2CH_2NHCH_2\dot{O}$	$\rightarrow$	$CHONHCH_2CH_2\dot{N}CH_2OH$	(17a)
$\Delta H^{\ominus} = -60$		$\rightarrow$	$CHONHCH_2\dot{C}HNHCH_2OH$	(17b)
$\Delta H^{\ominus} = -46$		$\rightarrow$	$CHONH\dot{C}HCH_2NHCH_2OH$	(17c)
$\Delta H^{\ominus} = +25$		$\rightarrow$	$CHO\dot{N}CH_2CH_2NHCH_2OH$	(17d)
$\Delta H^{\ominus} = -45$		$\rightarrow$	$\dot{C}ONHCH_2CH_2NHCH_2OH$	(17e)

The barrier to the dissociation reaction (15) is calculated to be around 82 kJ mol^{-1} and the rate is estimated from conventional TST to be around 1 s^{-1}. Assuming the $CHONHCH_2CH_2NHCH_2\dot{O}$ radicals to be thermalized and a typical rate coefficient of 1 x 10^{-14} cm^3 molecule^{-1} s^{-1} for reaction (16),[19] places the rate for this reaction to around 5 × 10^4 s^{-1} at atmospheric conditions. The barriers to reactions (17a) – (17e) are calculated to be around 90, 94, 54, 75 and 90 kJ mol^{-1}, respectively, suggesting that only the

1,5 H-shift reaction (17c) may compete with reaction (16). A higher-level calculation (CCSD(T*)-F12a/aug-cc-pVTZ//M06-2X/aug-cc-pVTZ) predicts a barrier of 47 kJ mol^{-1} for reaction (17c). A TST calculation places the unimolecular reaction (17c) with a rate of 6×10^3 s^{-1}, which is around 10% of the rate for reaction (16). It is therefore concluded that the H-abstraction reaction (16) and the internal H-transfer reaction (17c) will dominate the atmospheric fate of the $CHONHCH_2CH_2NHCH_2\dot{O}$ radical.

The $CHONH\dot{C}HCH_2NHCH_2OH$ radical will add O_2 and subsequently react with NO to the corresponding oxy radical, $CHONHCH(\dot{O})CH_2NHCH_2OH$ radical, that is calculated to dissociate upon formation:

$\Delta H^{\Theta} = -131$	$CHONH\dot{C}HCH_2NHCH_2OH + O_2$	$\rightarrow$	$CHONHCH(O\dot{O})CH_2NHCH_2OH$	(18)
$\Delta H^{\Theta} = -78$	$CHONHCH(O\dot{O})CH_2NHCH_2OH + NO$	$\rightarrow$	$CHONHCHO + \dot{C}H_2NHCH_2OH + NO_2$	(19)

The alkyl radical formed in (19) with add O_2 forming a vibrationally excited peroxy radical that may initiate internal H-transfer resulting in an imine before being quenched by collisions and or reacting with NO to give an oxy radical:

$\Delta H^{\Theta} = -135$	$\dot{C}H_2NHCH_2OH + O_2$	$\rightarrow$	$\dot{O}OCH_2NHCH_2OH^{\ddagger}$	(20)
$\Delta H^{\Theta} = -48$	$\dot{O}OCH_2NHCH_2OH^{\ddagger}$	$\rightarrow$	$CH_2{=}NCH_2OH + HO_2$	(21)
$\Delta H^{\Theta} = -51$	$\dot{O}OCH_2NHCH_2OH + NO$	$\rightarrow$	$\dot{O}CH_2NHCH_2OH + NO_2$	(22)

The branching between reactions (21) and (22) was estimated in master equation calculations based on the PES illustrated in Figure S5 (the underlying quantum chemistry data are collected in Table S6), and a collisional quenching of $\langle\Delta E_{down}\rangle = 250$ cm^{-1}, a typical rate coefficient of 4×10^{-12} cm^3 molecule^{-1} s^{-1} for reaction (22), and a mixing ratio of 2 ppb for NO. The calculations show that the brancing is virtually independent of $\langle\Delta E_{down}\rangle$ and the NO mixing ratio implying a 100 % yield of the oxy-radical. Lowering the barrier to reaction (21) by 10 kJ mol^{-1} does not change the oxy-radical yield. It can therefore be concluded that the imine formation, reaction (21), can be neglected at atmospheric conditions.

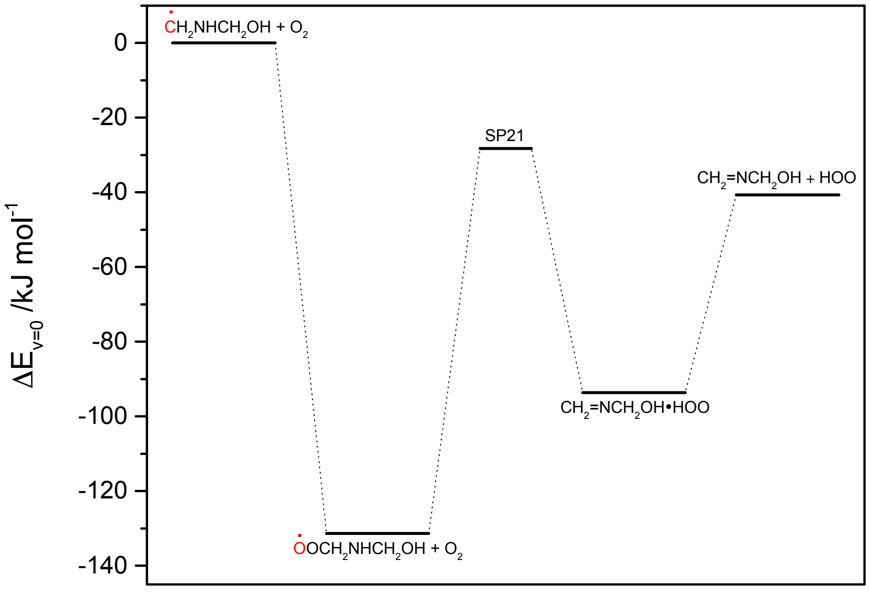

Figure S5.
Stationary points on the PES for the $\dot{C}H_2NHCH_2OH + O_2$ reaction. Results from M06-2X/aug-cc-pVTZ calculations.

The oxy radical formed in (22) may either undergo H-abstraction by O_2 leading to an amide or dissociate:

$\Delta H^{\Theta} = -196$	$\dot{O}CH_2NHCH_2OH + O_2$	$\rightarrow$	$CHONHCH_2OH + HO_2$	(23)
$\Delta H^{\Theta} = +52$	$\dot{O}CH_2NHCH_2OH$	$\xrightarrow{\Delta}$	$\dot{N}HCH_2OH + CH_2O$	(24)

With a barrier of more than 50 kJ mol^{-1} reaction (24) will be slow to compete with reaction (23).

In summery, the atmospheric fate of the HN<$\genfrac{}{}{0pt}{}{CH_2\dot{C}H}{CH_2CH_2}$>NH radical is assessed to follow the routes shown below:

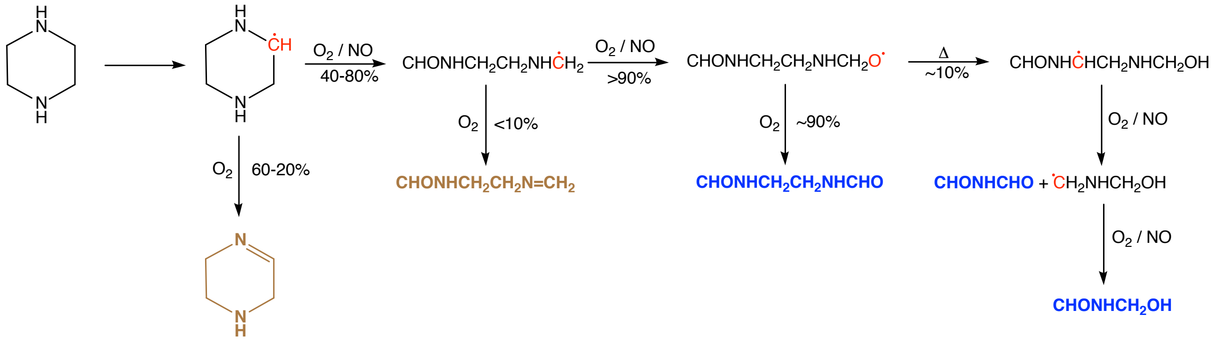

Table S2.

Comparison of quantum chemistry results for the $\underline{CH_2CH_2NHCH_2CH_2\dot{N}}$ + O_2 and $(CH_3)_2N$ + O_2 reactions. Electronic energies of reactants, intermediates and products (/Hartree), and relative energies including Zero Point Energies, $\Delta E_{v=0}$ (/kJ mol^{-1}), of stationary points on the potential energy surface.

	M06-2X/aug-cc-pVTZ			CCSD(T*)-F12a/aTZ //M06-2X/aTZ	
Species	E_{Elec}	E_{ZPE}	$\Delta E_{v=0}$	E_{Elec}	$\Delta E_{v=0}$
$\underline{CH_2CH_2NHCH_2CH_2\dot{N}}$	-267.250385	0.134765		-266.926695	
O_2	-150.324795	0.003997		-150.191136	
Sum Reactants	-417.575180	0.138762	0	-417.117831	0
$\underline{CH_2CH_2NHCH_2CH_2N}O\dot{O}$	-417.590460	0.144171	-25.9	-417.135496	-32.2
SP-2	-417.549315	0.135810	60.2	-417.103482	29.9
$\underline{CH_2CH_2NHCH_2CH=N}\bullet HO_2$	-417.622645	0.141815	-116.6	-417.168065	-123.9
$\underline{CH_2CH_2NHCH_2CH=N}$	-266.692840	0.124384		-266.367913	

HO_2	-150.908095	0.014578		-150.778909	
Sum Products	-417.600935	0.138963	-67.1	-417.146822	-75.6
$(CH_3)_2\dot{N}$	-134.484965	0.077849		-134.325741	
O_2	-150.324795	0.003997		-150.191136	
Sum Reactants	-284.809760	0.081846	0	-284.516877	0
$(CH_3)_2NO\dot{O}$	-284.823872	0.088303	-20.1	-284.533196	-25.9
SP	-284.789250	0.082537	55.7	-284.509362	21.5
$CH_3N{=}CH_2{\bullet}HO_2$	-284.853258	0.086037	-103.2	-284.564017	-112.8
$CH_3N{=}CH_2$	-133.924870	0.068750		-133.765118	
HO_2	-150.908108	0.014608		-150.778909	
Sum Products	-284.832977	0.083358	-57.0	-284.544028	-67.3

Table S3.

Quantum chemistry results for the $\underline{CH_2CH_2NHCH_2CH_2\dot{N}}$ + NO_2 reaction. Electronic energies of reactants, intermediates and products (/Hartree), and relative energies including Zero Point Energies, $\Delta E_{v=0}$ (/kJ mol^{-1}), of stationary points on the potential energy surface.

	M06-2X/aug-cc-pVTZ		
Species	E_{Elec}	E_{ZPE}	$\Delta E_{v=0}$
$\underline{CH_2CH_2NHCH_2CH_2\dot{N}}$	-267.250385	0.134765	
NO_2	-205.074635	0.009168	
Sum Reactants	-472.325020	0.143933	0
$\underline{CH_2CH_2NHCH_2CH_2N}NO_2$	-472.414728	0.152436	-213.2
SP-4a	-472.320181	0.143681	12.0
$\underline{CH_2CH_2NHCH_2CH{=}N}{\bullet}HO_2$	-472.422081	0.147161	-246.4
$\underline{CH_2CH_2NHCH_2CH{=}N}$	-266.692840	0.124384	
HONO	-205.709185	0.020954	
Sum Products	-472.402026	0.145339	-198.5
$\underline{CH_2CH_2NHCH_2CH_2N}ONO$	-363.464828	0.139428	-77.5
$\underline{CH_2CH_2NHCH_2CH_2N}\dot{O}$	-342.469636	0.140705	
NO	-129.893351	0.004707	
Sum Products	-472.362987	0.145412	-95.8

Table S3, continued: Vibrational frequencies ($\tilde{\nu}$: /cm^{-1}), Rotational constants (B /GHZ) and Cartesian Coordinates (/Å) of the species listed above

$CH_2CH_2NHCH_2CH_2\dot{N}$

$\tilde{\nu}$: 232.6, 236.5, 383.6, 447.4, 470.5, 590.2, 787.6, 853.8, 861.2, 905.8, 913.6, 1013.0, 1052.9, 1053.3, 1112.2, 1132.9, 1181.8, 1190.7, 1245.9, 1269.9, 1312.9, 1316.9, 1344.8, 1353.8, 1415.0, 1473.4, 1481.9, 1497.8, 1502.6, 1513.5, 2985.6, 2986.3, 3007.6, 3013.9, 3110.5, 3110.5, 3112.5, 3114.0, 3566.0

B: 4.8898649 4.7907335 2.7234728

C	-0.018137	-0.811324	1.185037
C	-0.018137	0.721086	1.199751
N	0.653579	1.197157	0.000000
C	-0.018137	0.721086	-1.199751
C	-0.018137	-0.811324	-1.185037
N	-0.680058	-1.299597	0.000000
H	-0.525272	-1.197361	2.068100
H	1.026409	-1.148139	1.197602
H	0.514890	1.075812	2.081090
H	-1.059816	1.066306	1.264324
H	0.708217	2.206706	0.000000
H	0.514890	1.075812	-2.081090
H	-1.059816	1.066306	-1.264324
H	1.026409	-1.148139	-1.197602
H	-0.525272	-1.197361	-2.068100

NO_2 $\tilde{\nu}$: 783.5, 1465.2, 1775.4
B: 253.9647127 13.2906276 12.6296837

N	0.000000	0.314443	0.000000
O	1.090260	-0.137566	0.000000
O	-1.090260	-0.137572	0.000000

$CH_2CH_2NHCH_2CH_2NNO_2$

$\tilde{\nu}$: 96.4, 100.7, 201.1, 322.7, 405.7, 416.6, 455.2, 483.4, 578.1, 621.6, 635.7, 792.9, 820.4, 851.4, 857.6, 899.7, 928.6, 1003.8, 1063.4, 1069.1, 1085.2, 1157.8, 1182.0, 1194.4, 1224.5, 1306.4, 1308.7, 1355.5, 1363.3, 1374.5, 1383.2, 1411.6, 1430.6, 1473.7, 1487.6, 1499.3, 1502.4, 1512.7, 1660.7, 2988.0, 2989.4, 3103.1, 3107.4, 3118.1, 3119.3, 3196.8, 3197.2, 3574.6

B: 2.8506873 1.5137742 1.2031659

C	0.369412	1.223297	0.759867
C	1.337009	1.207980	-0.414891
N	2.152414	0.000000	-0.335184
C	1.337009	-1.207980	-0.414891
C	0.369412	-1.223297	0.759867
N	-0.436960	0.000000	0.761494
N	-1.515098	0.000000	-0.098271
O	-1.967887	-1.077203	-0.426501
O	-1.967887	1.077203	-0.426501
H	-0.288809	2.083151	0.740218
H	0.925263	1.212811	1.696489
H	1.985019	2.081879	-0.363676
H	0.755862	1.266116	-1.346818
H	2.851992	0.000000	-1.064391
H	1.985019	-2.081879	-0.363676
H	0.755862	-1.266116	-1.346818
H	0.925263	-1.212811	1.696489
H	-0.288809	-2.083151	0.740218

SP-4a

$\tilde{\nu}$: -1502.9, 70.7, 120.0, 149.4, 215.4, 267.8, 315.7, 425.9, 490.8, 538.1, 574.0, 595.5, 750.9, 768.6, 834.7, 852.0, 863.5, 922.7, 953.9, 1021.4, 1033.1, 1058.6, 1133.6, 1183.4, 1217.2, 1258.8, 1284.5, 1303.0, 1326.5, 1337.5, 1368.5, 1370.8, 1407.2, 1472.4, 1495.1, 1498.3, 1516.5, 1519.0, 1600.7, 1802.7, 3034.1, 3040.0, 3048.2, 3094.2, 3097.8, 3125.1, 3132.4, 3578.6

B: 2.5612773 1.3966540 1.2594375

C	-0.626784	-1.140608	0.766260
C	-1.823456	-0.896327	-0.138671
N	-1.530243	0.121251	-1.140779
C	-0.985865	1.359914	-0.572220
C	-0.530878	1.219719	0.885164
N	0.024098	-0.072837	1.254858
N	1.594823	-0.075385	-0.046717
O	2.510659	0.667458	-0.194877
O	1.510838	-1.179962	-0.606979
H	0.372806	-1.482163	-0.027376
H	-0.612977	-2.010881	1.423763
H	-2.679494	-0.564015	0.456935
H	-2.124697	-1.814393	-0.639373
H	-0.881538	-0.257521	-1.816040
H	-1.729575	2.158845	-0.627884
H	-0.131763	1.676172	-1.172138
H	-1.388692	1.329701	1.558519

H	0.185116	1.996899	1.143718

$CH_2CH_2NHCH_2CH{=}N{\bullet}HO_2$

$\tilde{\nu}$: 17.8, 42.2, 55.7, 115.5, 164.4, 195.5, 199.8, 311.6, 426.2, 484.6, 546.4, 717.0, 771.9, 797.7, 853.6, 877.6, 919.6, 968.2, 1022.3, 1038.9, 1055.9, 1066.9, 1085.0, 1121.5, 1195.7, 1232.1, 1267.6, 1290.1, 1353.5, 1375.6, 1413.7, 1430.2, 1484.1, 1484.9, 1488.1, 1510.3, 1558.1, 1782.9, 1800.2, 2960.2, 2982.0, 3002.1, 3078.4, 3087.2, 3120.5, 3126.9, 3132.0, 3583.9

B: 4.2529202 0.6408952 0.5757092

C	0.580391	0.990668	-0.397827
C	1.908369	1.327131	0.251563
N	0.210789	-0.415433	-0.231929
N	-2.871363	0.327428	0.208200
O	-2.451606	-0.829175	-0.343820
C	1.098633	-1.257904	0.077490
C	2.559024	-0.973963	0.268447
N	2.889984	0.353177	-0.207438
O	-4.042252	0.389493	0.287873
H	0.618199	1.195078	-1.469052
H	-0.229387	1.586292	0.023306
H	1.788959	1.317110	1.344987
H	2.224603	2.325798	-0.043563
H	-1.449324	-0.741859	-0.339560
H	0.782961	-2.289069	0.228207
H	2.777885	-1.133220	1.337265
H	3.126558	-1.725070	-0.284120
H	3.826037	0.610602	0.070236

$CH_2CH_2NHCH_2CH{=}N$

$\tilde{\nu}$: 177.2, 310.3, 421.5, 476.2, 541.3, 712.5, 797.3, 855.1, 872.3, 913.7, 962.8, 1035.5, 1063.0, 1084.8, 1118.3, 1193.7, 1227.8, 1261.3, 1286.3, 1347.5, 1369.9, 1406.9, 1424.9, 1481.0, 1484.9, 1487.8, 1509.5, 1782.6, 2954.5, 2971.1, 3070.0, 3083.2, 3107.7, 3111.8, 3114.2, 3580.0

B: 5.2047928 4.8943778 2.7589170

C	-0.637567	-1.147006	0.308190
C	-1.424348	0.068204	-0.147401
N	-0.686253	1.322736	-0.032561
C	0.569306	1.267688	0.068554
C	1.425351	0.029330	0.054620
N	0.653327	-1.123714	-0.366697
H	-1.167829	-2.060439	0.042497
H	-0.534473	-1.120359	1.403778
H	-2.340292	0.173393	0.434658
H	-1.717199	-0.045186	-1.193115
H	1.106691	2.209539	0.180745
H	1.862694	-0.076966	1.061195
H	2.255812	0.194519	-0.634357
H	1.168623	-1.976946	-0.204370

HONO

$\tilde{\nu}$: 578.3, 700.4, 904.9, 1342.4, 1841.1, 3830.7

B: 97.1291438 13.0242864 11.4843250

N	0.000000	0.507869	0.000000
O	0.882545	-0.567211	0.000000
O	-1.101706	0.141499	0.000000
H	1.753285	-0.149388	0.000000

$CH_2CH_2NHCH_2CH_2NONO$

$\tilde{\nu}$: 64.3, 110.9, 220.5, 253.9, 267.8, 361.2, 414.6, 451.7, 460.6, 506.5, 595.3, 728.4, 773.9, 818.7, 846.4, 896.6, 933.4, 968.3, 1006.8, 1040.6, 1049.5, 1079.2, 1165.2, 1179.6, 1202.0, 1221.7, 1305.1, 1314.7, 1350.8, 1354.8, 1374.5, 1400.8, 1401.8, 1481.4, 1484.8, 1493.2, 1501.9, 1515.6, 1802.1, 2995.5, 3001.2, 3071.6, 3073.3, 3121.2, 3122.9, 3127.4, 3127.8, 3534.7

B: 3.6483669 1.0883293 0.9515369

C	1.912196	-0.955191	0.548192
C	0.598744	-1.331193	-0.133202
N	-0.331205	-0.219493	0.030044
C	0.182024	1.001331	-0.586787
C	1.497847	1.363333	0.097269
N	2.480707	0.289915	0.050926
O	-2.548237	0.571403	0.836669
N	-2.692925	-0.143554	-0.066961
O	-1.507536	-0.603480	-0.659917
H	2.634540	-1.759439	0.416540
H	1.733305	-0.839325	1.619005
H	0.157433	-2.213672	0.327145
H	0.772230	-1.539521	-1.198289
H	-0.554551	1.791698	-0.448947
H	0.340059	0.847262	-1.663087
H	1.297259	1.591716	1.145917
H	1.916802	2.256716	-0.363253
H	2.808204	0.163410	-0.899938

$CH_2CH_2NHCH_2CH_2\dot{N}O$ (cyclic)	C	-0.118772	-1.091024	1.198699
	C	-0.118772	0.427941	1.242783
	N	0.428318	0.961512	0.000000
$\tilde{\nu}$: 126.5, 245.3, 291.0, 404.5, 438.0, 439.9, 491.1, 585.7, 779.8, 793.8, 848.0, 913.0, 947.7, 1048.5, 1065.2, 1071.6, 1132.7, 1179.7, 1191.2, 1211.6, 1272.4, 1313.9, 1319.0, 1349.7, 1362.9, 1384.2, 1430.0, 1477.3, 1484.0, 1485.6, 1499.5, 1506.6, 1517.3, 2984.2, 2984.3, 3037.1, 3038.8, 3117.3, 3118.0, 3149.8, 3150.8, 3574.5	C	-0.118772	0.427941	-1.242783
	C	-0.118772	-1.091024	-1.198699
	N	-0.819819	-1.529784	0.000000
	O	0.903612	2.136262	0.000000
	H	-0.631336	-1.476952	2.078552
	H	0.922267	-1.444307	1.222802
	H	0.497973	0.814676	2.050004
B: 4.7076738 2.5103116 1.7736377	H	-1.143383	0.796167	1.361918
	H	0.497973	0.814676	-2.050004
	H	-1.143383	0.796167	-1.361918
	H	0.922267	-1.444307	-1.222802
	H	-0.631336	-1.476952	-2.078552
	H	-0.928900	-2.534361	0.000000
NO $\tilde{\nu}$: 2066.0	N	0.000000	0.000000	-0.606410
B: 52.3561936	O	0.000000	0.000000	0.530609

Table S4.
Quantum chemistry results for the $CH_2CH_2NHCH_2CH_2\dot{N}$ (cyclic) + O_2 reaction Electronic energies of reactants, intermediates and products (/Hartree), and relative energies including Zero Point Energies, $\Delta E_{v=0}$ (/kJ mol^{-1}), of stationary points on the potential energy surface of the $CH_2CH_2NHCH_2CH_2\dot{N}$ (cyclic) + O_2 reaction.

	M06-2X/aug-cc-pVTZ		
Species	E_{Elec}	E_{ZPE}	$\Delta E_{v=0}$
$NHCH_2CH_2NHCH_2\dot{C}H$ (cyclic)	-267.255315	0.135394	
O_2	-150.324795	0.003997	
Sum Reactants	-417.580111	0.139391	0
$NHCH_2CH_2NHCH_2CHO\dot{O}$ (cyclic)	-417.640164	0.145438	-141.8
SP9a	-417.601715	0.139600	-56.2
$CH_2CH_2NHCH_2CH{=}N{\bullet}HOO$ (cyclic)	-417.622803	0.141334	-107.0
$CH_2CH_2NHCH_2CH{=}N$ (cyclic)	-266.692840	0.124384	
HO_2	-150.908095	0.014578	
Sum Products	-417.600935	0.138963	-55.8
SP9c	-417.591292	0.140471	-26.5
$NH\dot{C}HCH_2NHCH_2CHOOH$ (cyclic)	-417.617028	0.142608	-88.5
SP9d	-417.590804	0.139844	-26.9
$NHCH_2\dot{C}HNHCH_2CHOOH$ (cyclic)	-417.624952	0.144089	-105.4

SP9b	-417.588956	0.138352	-26.0
$CH_2CH_2NHCH{=}CHNH$•HOO	-417.618427	0.141124	-96.1
$CH_2CH_2NHCH{=}CHNH$	-266.695240	0.124582	
HO_2	-150.908095	0.014578	
Sum products	-417.603335	0.139160	-61.6

Table S4, continued. Vibrational frequencies ($\tilde{\nu}$: /cm^{-1}), Rotational constants (B /GHZ) and Cartesian Coordinates (/Å) of the species listed in Table S3a.

$NHCH_2CH_2NHCH_2\dot{C}H$

$\tilde{\nu}$: 240.5, 257.0, 396.4, 445.0, 486.9, 521.2, 650.5, 724.1, 831.1, 872.8, 923.8, 961.6, 977.0, 1078.2, 1094.6, 1119.0, 1169.8, 1211.0, 1228.7, 1266.4, 1299.5, 1346.8, 1371.2, 1422.6, 1447.0, 1485.2, 1486.0, 1499.9, 1508.5, 1513.6, 2876.0, 2971.1, 3023.0, 3107.4, 3112.0, 3114.0, 3211.6, 3564.9, 3615.0

B: 5.0149649 4.7124731 2.6617911

C	0.041260	-1.352146	0.259639
C	-1.241422	-0.677683	-0.192856
N	-1.196698	0.731107	0.181921
C	-0.037480	1.421858	-0.148363
C	1.247560	0.716286	0.122689
N	1.171264	-0.665776	-0.351098
H	0.036844	-2.393273	-0.060811
H	0.081388	-1.326923	1.359440
H	-2.101407	-1.141753	0.288481
H	-1.331512	-0.795125	-1.278555
H	-2.047053	1.232054	-0.021954
H	-0.079573	2.495318	-0.034581
H	1.466269	0.744463	1.208728
H	2.061742	1.228366	-0.388380
H	2.031832	-1.150331	-0.134778

O_2

$\tilde{\nu}$: 1754.5, B: 44.6355338

O	0.000000	0.000000	0.594925
O	0.000000	0.000000	-0.594925

$NHCH_2CH_2NHCH_2CHO\dot{O}$

$\tilde{\nu}$: 82.2, 168.3, 189.3, 324.8, 334.9, 411.2, 452.9, 524.0, 594.7, 677.4, 744.5, 780.1, 840.4, 872.7, 925.2, 964.6, 1025.9, 1085.1, 1099.6, 1124.8, 1174.5, 1214.3, 1225.1, 1262.7, 1302.1, 1315.9, 1350.6, 1358.9, 1363.8, 1427.8, 1452.2, 1482.1, 1488.9, 1503.4, 1510.4, 1528.2, 2979.5, 2994.4, 3039.4, 3114.0, 3118.9, 3121.6, 3122.8, 3582.7, 3583.4

B: 3.5421955 1.7827543 1.4699386

C	-0.763364	1.414162	-0.171964
C	-1.926680	0.445071	-0.265555
N	-1.395293	-0.873273	-0.583711
C	-0.516765	-1.363322	0.463133
C	0.618824	-0.383996	0.675290
N	0.160856	0.937830	0.857835
O	1.494063	-0.534846	-0.523060
O	2.469661	0.312954	-0.484800
H	-1.117472	2.401027	0.121105
H	-0.282587	1.489184	-1.152800
H	-2.603891	0.755508	-1.059617
H	-2.474741	0.457427	0.687996
H	-2.135023	-1.535820	-0.766584
H	-0.103221	-2.327273	0.170339
H	-1.019752	-1.477108	1.433755
H	1.255280	-0.677337	1.508827
H	0.940576	1.566142	0.995567

SP9a

$\tilde{\nu}$: -688.2, 77.7, 142.8, 173.3, 187.2, 321.9, 384.0, 451.4, 504.4, 559.7, 688.1, 766.1, 802.7, 866.1, 873.2, 917.6, 954.9, 1018.1, 1046.6, 1067.2, 1140.4, 1194.4, 1224.4, 1292.0, 1321.1, 1329.8, 1365.4, 1373.4, 1386.4, 1404.3, 1463.4, 1488.7, 1492.2, 1499.3, 1535.6, 1666.4, 2022.5, 3048.9, 3065.6, 3074.0, 3097.9, 3119.0, 3130.8, 3184.8, 3553.2

C	1.317061	0.800317	-0.803177
C	0.571880	1.449080	0.353055
N	-0.339632	0.501230	1.015822
C	-0.195076	-0.770470	0.794476
C	0.988612	-1.388038	0.117800
N	1.965824	-0.438373	-0.387062
O	-1.850657	-0.668426	-0.723378
O	-2.371841	0.406853	-0.264990
H	2.062953	1.482320	-1.207911

B: 3.3035886 1.6433413 1.4141195

H	0.612592	0.559086	-1.602028
H	-0.030508	2.291602	0.014854
H	1.276965	1.823714	1.097574
H	-1.429629	0.703700	0.731133
H	-0.908100	-1.436272	1.265990
H	0.593144	-1.995859	-0.702200
H	1.468216	-2.082550	0.809675
H	2.656140	-0.238487	0.325617

$CH_2CH_2NHCH_2CH{=}N{\bullet}HOO$

$\tilde{\nu}$: 31.2, 54.9, 83.6, 144.8, 166.7, 266.9, 295.1, 418.1, 500.2, 553.8, 728.3, 802.8, 818.5, 850.0, 878.7, 912.0, 935.8, 1033.5, 1048.6, 1066.9, 1135.8, 1195.0, 1225.7, 1277.6, 1290.8, 1330.7, 1362.8, 1374.3, 1401.4, 1408.8, 1466.3, 1483.7, 1493.1, 1494.9, 1661.2, 1772.7, 2898.0, 3031.6, 3056.6, 3064.2, 3102.8, 3104.7, 3124.1, 3140.6, 3550.5

B: 4.4460774 0.9884888 0.8339871

C	2.097148	-0.857246	0.350084
C	0.750162	-1.370322	-0.134809
N	-0.308894	-0.362066	-0.050650
C	0.003205	0.860889	0.048851
C	1.403327	1.410741	0.053023
N	2.435906	0.432451	-0.240496
O	-3.214484	0.649851	0.056578
O	-2.962450	-0.629816	-0.046496
H	2.884208	-1.576679	0.129374
H	2.069358	-0.726702	1.434776
H	0.424842	-2.234044	0.446062
H	0.813715	-1.697027	-1.176164
H	-1.950065	-0.674448	-0.061262
H	-0.818724	1.568372	0.145944
H	1.581998	1.856430	1.038605
H	1.457307	2.233959	-0.660391
H	2.540687	0.332799	-1.242473

$CH_2CH_2NHCH_2CH{=}N$

$\tilde{\nu}$: 177.2, 310.3, 421.5, 476.2, 541.3, 712.5, 797.3, 855.1, 872.3, 913.7, 962.8, 1035.5, 1063.0, 1084.8, 1118.3, 1193.7, 1227.8, 1261.3, 1286.3, 1347.5, 1369.9, 1406.9, 1424.9, 1481.0, 1484.9, 1487.8, 1509.5, 1782.6, 2954.5, 2971.1, 3070.0, 3083.2, 3107.7, 3111.8, 3114.2, 3580.0

B: 5.2047928 4.8943778 2.7589170

C	-0.637567	-1.147006	0.308190
C	-1.424348	0.068204	-0.147401
N	-0.686253	1.322736	-0.032561
C	0.569306	1.267688	0.068554
C	1.425351	0.029330	0.054620
N	0.653327	-1.123714	-0.366697
H	-1.167829	-2.060439	0.042497
H	-0.534473	-1.120359	1.403778
H	-2.340292	0.173393	0.434658
H	-1.717199	-0.045186	-1.193115
H	1.106691	2.209539	0.180745
H	1.862694	-0.076966	1.061195
H	2.255812	0.194519	-0.634357
H	1.168623	-1.976946	-0.204370

HO_2 $\tilde{\nu}$: 1252.8, 1459.4, 3686.8

B: 628.5431720 34.6992321 32.8838526

H	-0.880747	-0.865418	0.000000
O	0.055047	0.708193	0.000000
O	0.055047	-0.600015	0.000000

SP9c

$\tilde{\nu}$: -1896.7, 182.4, 272.0, 282.8, 359.2, 460.2, 504.5, 550.7, 590.8, 699.2, 714.0, 791.8, 825.8, 848.9, 899.4, 906.9, 952.4, 1009.6, 1035.2, 1083.1, 1085.1, 1141.2, 1161.6, 1200.6, 1237.3, 1282.8, 1296.6, 1311.5, 1344.8, 1354.2, 1386.4, 1424.6, 1474.8, 1486.9, 1492.4, 1505.3, 1672.1, 3078.6, 3080.7, 3112.2, 3125.3, 3137.1, 3151.4, 3554.2, 3593.2

B: 3.2507091 2.1499755 1.8144089

C	0.996998	1.082592	-0.666813
C	-0.098105	1.354865	0.374843
N	-0.222919	0.261234	1.270185
C	-0.304974	-0.954526	0.520769
C	1.086062	-1.260733	-0.060099
N	1.851283	-0.038388	-0.281843
O	-1.140247	-0.782989	-0.662755
O	-2.050731	0.240762	-0.509850
H	1.612376	1.970766	-0.799181
H	0.546433	0.845328	-1.631596
H	-1.241693	1.182082	-0.257795
H	-0.087596	2.317237	0.877951
H	-0.980369	0.377891	1.928029
H	-0.712866	-1.755352	1.135412

H	0.946910	-1.793727	-1.001535
H	1.640634	-1.903935	0.620553
H	2.325565	0.204412	0.578405

NHĊHCH$_2$NHCH$_2$CHOOH

$\tilde{\nu}$: 60.5, 132.9, 187.3, 215.6, 268.2, 305.7, 387.0, 417.9, 459.0, 510.2, 558.3, 676.5, 762.5, 792.1, 906.7, 924.0, 943.0, 972.9, 1019.4, 1069.5, 1109.8, 1118.7, 1179.2, 1220.5, 1242.9, 1306.9, 1336.5, 1351.7, 1374.9, 1388.1, 1408.0, 1447.0, 1485.0, 1493.2, 1494.8, 1503.2, 3013.0, 3037.9, 3059.1, 3083.6, 3134.0, 3228.8, 3551.2, 3623.7, 3836.5

B: 3.6728029 1.6648671 1.4554382

C	-2.020594	0.308969	0.472360
C	-0.958004	1.318428	0.152236
N	-0.044170	0.950263	-0.833375
C	0.596311	-0.332666	-0.740896
C	-0.222101	-1.256393	0.161451
N	-1.653650	-1.039995	0.033377
O	1.965372	-0.241180	-0.344175
O	2.032721	0.378982	0.937142
H	-2.978825	0.560789	0.004193
H	-2.202385	0.290233	1.551544
H	-1.144322	2.374837	0.266721
H	0.567458	1.677397	-1.169593
H	0.717363	-0.763906	-1.738863
H	0.054278	-1.057029	1.197686
H	0.024329	-2.292846	-0.063292
H	-1.929182	-1.166965	-0.932308
H	2.417614	-0.326831	1.469261

SP9d

$\tilde{\nu}$: -1863.0, 110.1, 221.0, 301.7, 348.6, 394.5, 455.7, 513.2, 579.0, 616.7, 704.8, 732.9, 819.7, 887.6, 906.3, 943.0, 959.8, 1013.2, 1052.6, 1074.5, 1105.9, 1128.4, 1185.4, 1236.9, 1254.8, 1272.5, 1286.5, 1335.9, 1358.7, 1364.7, 1425.6, 1436.2, 1479.5, 1501.7, 1507.5, 1516.8, 1569.5, 3038.6, 3072.3, 3079.7, 3117.3, 3124.2, 3150.4, 3594.1, 3606.7

B: 2.7185252 2.2827683 2.0690009

C	1.117230	-0.552975	-0.763789
C	1.334713	0.926693	-0.530565
N	0.455525	1.376911	0.556810
C	-0.688137	0.542079	0.774117
C	-0.222189	-0.832200	1.233094
N	0.928961	-1.272062	0.433174
O	-1.566346	0.488785	-0.372293
O	-1.322835	-0.610905	-1.182360
H	1.847126	-1.010921	-1.425030
H	-0.020177	-0.601883	-1.327094
H	1.133067	1.456377	-1.465098
H	2.375256	1.111834	-0.251484
H	0.176276	2.338152	0.426893
H	-1.306681	1.001435	1.543512
H	-1.049668	-1.536803	1.150993
H	0.074240	-0.745241	2.277895
H	0.942912	-2.271515	0.299599

NHCH$_2$ĊHNHCH$_2$CHOOH

$\tilde{\nu}$: 113.4, 154.7, 203.1, 299.5, 330.2, 378.2, 445.6, 461.9, 500.3, 586.9, 632.8, 672.2, 766.2, 819.1, 903.6, 930.9, 944.2, 963.5, 1056.8, 1072.5, 1096.9, 1135.1, 1164.8, 1240.2, 1268.5, 1289.5, 1351.9, 1369.8, 1380.0, 1419.1, 1450.0, 1467.1, 1472.3, 1479.3, 1493.0, 1520.3, 2996.5, 2999.8, 3070.3, 3124.4, 3128.9, 3208.0, 3564.6, 3622.4, 3699.3

B: 3.6137138 1.7484047 1.4264120

C	1.935839	0.551818	-0.282129
C	0.755096	1.452772	-0.143713
N	-0.250912	0.947899	0.802456
C	-0.569974	-0.452096	0.584806
C	0.639368	-1.365823	0.427929
N	1.632751	-0.788218	-0.461077
O	-1.321955	-0.560824	-0.602384
O	-2.531332	0.155483	-0.406357
H	2.815594	0.907711	-0.799164
H	0.248234	1.563530	-1.111537
H	1.060828	2.447171	0.174030
H	0.051968	1.087870	1.757983
H	-1.177581	-0.795029	1.426030
H	0.310969	-2.326396	0.034699
H	1.051903	-1.518315	1.433803
H	2.425341	-1.387685	-0.624473
H	-2.195808	1.026080	-0.132453

SP9b

C	1.272787	-0.667876	-0.890260
C	1.685323	0.764955	-0.551113

$\tilde{\nu}$: -1092.5, 97.5, 102.9, 182.0, 197.3, 337.1, 345.4, 475.7, 510.6, 536.6, 560.7, 702.1, 766.4, 825.1, 874.4, 887.7, 946.8, 966.9, 991.5, 1053.4, 1098.7, 1133.2, 1191.4, 1259.1, 1268.4, 1302.1, 1333.6, 1358.9, 1365.8, 1390.2, 1418.4, 1468.4, 1494.2, 1511.8, 1551.1, 1599.3, 1632.0, 3055.9, 3082.3, 3110.5, 3136.2, 3176.8, 3248.5, 3530.4, 3652.2

B: 2.9353284 1.6905810 1.4729240

N	0.704236	1.356805	0.356441
C	-0.178642	0.621685	1.031175
C	-0.169463	-0.791183	1.001028
N	0.920211	-1.436268	0.293250
O	-2.033082	0.688100	-0.452130
O	-2.169930	-0.571849	-0.620927
H	2.081435	-1.163235	-1.424665
H	0.398182	-0.646209	-1.542177
H	1.741484	1.376949	-1.451213
H	2.669786	0.768906	-0.077983
H	0.526948	2.343310	0.277420
H	-0.873238	1.158949	1.657651
H	-1.201515	-0.934046	0.248302
H	-0.478405	-1.292716	1.910669
H	1.728253	-1.551149	0.893633

$CH_2CH_2NHCH{=}CHNH{\bullet}HOO$

$\tilde{\nu}$: 30.4, 64.1, 71.5, 124.8, 155.5, 218.7, 296.4, 474.5, 509.3, 523.0, 531.5, 564.6, 668.1, 755.4, 804.1, 871.1, 928.9, 981.8, 992.2, 1062.4, 1089.1, 1137.2, 1188.4, 1265.1, 1278.1, 1295.6, 1308.6, 1354.2, 1389.0, 1440.7, 1468.4, 1480.0, 1496.7, 1503.0, 1512.1, 1709.9, 3051.8, 3063.6, 3130.6, 3134.6, 3210.1, 3227.3, 3385.4, 3588.0, 3610.0

B: 2.7097683 1.4741438 1.3589465

C	1.189284	-0.153763	-1.183171
C	1.413388	1.158139	-0.417613
N	0.431498	1.323881	0.636592
C	0.133417	0.151174	1.338673
C	0.601447	-1.054574	0.971194
N	1.192339	-1.289964	-0.277678
O	-2.501416	0.561032	-0.311923
O	-2.202994	-0.702071	-0.461950
H	1.948895	-0.302788	-1.948018
H	0.218064	-0.102265	-1.681910
H	1.368672	2.014184	-1.087547
H	2.407781	1.133255	0.034560
H	-0.386668	1.853580	0.372728
H	-0.445573	0.263393	2.244315
H	-1.415772	-0.851417	0.115479
H	0.493092	-1.912797	1.619262
H	2.054713	-1.810094	-0.244790

$CH_2CH_2NHCH{=}CHNH$

$\tilde{\nu}$: 178.8, 293.0, 476.3, 514.0, 520.4, 567.2, 664.7, 739.4, 800.0, 870.2, 934.7, 982.4, 986.2, 1062.3, 1085.5, 1139.0, 1186.2, 1264.6, 1292.8, 1308.9, 1353.5, 1388.0, 1437.9, 1465.0, 1474.1, 1492.8, 1502.1, 1728.6, 3045.8, 3057.8, 3125.7, 3126.0, 3210.3, 3229.3, 3590.2, 3591.3

B: 5.2863977 4.8692609 2.7604335

C	0.690876	-1.130018	0.334654
C	-0.690877	-1.130000	-0.334714
N	-1.440677	0.061744	0.012816
C	-0.664996	1.233363	0.069221
C	0.664997	1.233366	-0.069156
N	1.440677	0.061743	-0.012813
H	1.265293	-2.012128	0.056924
H	0.550388	-1.146343	1.418573
H	-1.265296	-2.012123	-0.057030
H	-0.550389	-1.146267	-1.418633
H	-2.066682	-0.055730	0.795030
H	-1.209360	2.161798	0.162571
H	1.209363	2.161805	-0.162456
H	2.066681	-0.055691	-0.795033

Table S5.

Quantum chemistry results for the $CHONHCH_2CH_2NH\dot{C}H_2 + O_2$ reaction. Electronic energies of reactants, intermediates and products (/Hartree), and relative energies including Zero Point Energies, $\Delta E_{v=0}$ (/kJ mol^{-1}), of stationary points on the potential energy surface.

	M06-2X/aug-cc-pVTZ

Species	E_{Elec}	E_{ZPE}	$\Delta E_{v=0}$
$CHONHCH_2CH_2NH\dot{C}H_2$	-342.500341	0.136388	
O_2	-150.324795	0.003997	
Sum Reactants	-492.825137	0.140385	0.0
$CHONHCH_2CH_2NHCH_2O\dot{O}$	-492.881996	0.147372	-130.9
SP13	-492.841658	0.141049	-41.6
$CHONHCH2CH2N{=}CH_2{\bullet}HOO$	-492.869953	0.143779	-108.8
$CHONHCH_2CH_2N{=}CH_2$	-341.935922	0.126056	
HO_2	-150.908095	0.014578	
Sum products	-492.844017	0.140634	-48.9

Table S5, continued. Vibrational frequencies ($\tilde{\upsilon}$/cm^{-1}), Rotational constants (B /GHZ) and Cartesian Coordinates (/Å) of the species above

$CHONHCH_2CH_2NH\dot{C}H_2$	C	1.153817	-1.030318	0.396942
	C	-0.224137	-0.916009	-0.244431
$\tilde{\upsilon}$: 15.5, 105.3, 141.9, 231.8, 240.1, 309.1, 347.2, 410.7, 521.8, 588.6, 618.2, 694.0, 773.6, 862.1, 901.5, 1037.1, 1047.6, 1057.3, 1097.6, 1177.6, 1240.5, 1276.1, 1286.3, 1332.3, 1383.1, 1396.0, 1427.5, 1463.9, 1489.7, 1507.5, 1538.1, 1550.0, 1802.4, 3013.4, 3036.7, 3074.8, 3122.3, 3125.7, 3158.7, 3274.0, 3575.7, 3614.1	N	-0.737098	0.433856	-0.148655
	C	-2.053119	0.696689	0.008863
	C	2.262564	1.146572	0.116474
	N	2.161682	-0.189397	-0.218281
	O	-2.921985	-0.141953	0.097781
	H	1.480749	-2.069024	0.344237
	H	1.093223	-0.752676	1.450197
B: 6.5280199 1.3397793 1.1673859	H	-0.931919	-1.575316	0.256722
	H	-0.167996	-1.232892	-1.290759
	H	-0.086246	1.200082	-0.250557
	H	-2.265942	1.775592	0.057969
	H	2.071733	1.391185	1.152264
	H	2.990135	1.728573	-0.427601
	H	2.385317	-0.422713	-1.173262
O_2	O	0.000000	0.000000	0.594925
$\tilde{\upsilon}$: 1754.5, B: 44.6355338	O	0.000000	0.000000	-0.594925
$CHONHCH_2CH_2NHCH_2O\dot{O}$	C	0.214686	-0.497248	0.477970
	C	-1.158451	-0.769650	-0.103881
$\tilde{\upsilon}$: 19.1, 55.2, 62.0, 98.1, 172.7, 225.1, 230.5, 307.2, 318.6, 423.5, 524.0, 575.8, 661.0, 749.8, 786.2, 838.5, 880.1, 905.7, 1031.4, 1047.7, 1081.7, 1097.6, 1175.1, 1221.1, 1230.2, 1239.4, 1282.4, 1300.0, 1333.0, 1382.1, 1402.4, 1416.1, 1429.2, 1493.0, 1504.8, 1509.8, 1525.6, 1537.8, 1806.6, 3011.4, 3038.5, 3048.5, 3099.7, 3106.6, 3139.5, 3171.9, 3549.5, 3643.4	N	-1.910346	0.461818	-0.213824
	C	-3.254494	0.508108	-0.079903
	C	2.113644	0.987903	0.188429
	N	0.928208	0.465368	-0.360416
	O	-3.957410	-0.446807	0.160934
	O	3.125599	-0.039921	0.505949
	O	3.421579	-0.726500	-0.551973
	H	0.760329	-1.439072	0.585063
B: 6.3140557 0.5558225 0.5354441	H	0.109190	-0.055522	1.470829
	H	-1.719279	-1.449956	0.533621
	H	-1.059015	-1.249917	-1.082724
	H	-1.404653	1.295677	-0.464975
	H	-3.658578	1.524576	-0.205093
	H	2.595892	1.674114	-0.503653
	H	1.935388	1.454628	1.155080
	H	1.105232	0.086319	-1.283442
SP13	C	0.019082	0.749930	-0.585687

$\tilde{\nu}$: -944.7, 18.9, 42.2, 84.5, 144.3, 167.2, 219.3, 264.4, 309.0, 325.0, 469.7, 484.9, 523.8, 622.1, 675.5, 775.7, 813.9, 897.8, 901.0, 1046.0, 1051.5, 1056.3, 1109.8, 1112.3, 1210.5, 1234.1, 1299.6, 1312.1, 1322.1, 1389.7, 1396.5, 1414.3, 1428.9, 1473.7, 1486.1, 1510.1, 1538.4, 1654.4, 1803.6, 1850.8, 3016.6, 3065.3, 3092.1, 3119.0, 3126.2, 3148.1, 3231.6, 3674.9

B: 3.6246893 0.7300321 0.6900458

C	-1.280676	1.319243	-0.028692
N	-2.007247	0.350729	0.770058
C	-2.664558	-0.683258	0.198676
C	1.143725	-0.923348	0.642375
N	0.905181	0.347827	0.504971
O	-2.730627	-0.869696	-0.995775
O	3.035544	-0.804743	-0.316876
O	3.174351	0.454360	-0.210883
H	0.516806	1.517438	-1.179361
H	-0.212198	-0.093089	-1.240596
H	-1.916298	1.628906	-0.857624
H	-1.072123	2.184066	0.597699
H	-1.938437	0.382836	1.771813
H	-3.146838	-1.347390	0.932198
H	1.695319	-1.266668	1.507978
H	0.617891	-1.664745	0.046360
H	2.030764	0.753979	0.324569

$CHONHCH_2CH_2N{=}CH_2{\bullet}HOO$

$\tilde{\nu}$: 36.8, 60.3, 70.4, 81.1, 124.0, 154.1, 190.3, 226.5, 276.5, 310.6, 375.2, 483.3, 546.7, 616.3, 713.7, 784.7, 882.1, 903.6, 923.2, 1049.4, 1053.5, 1062.8, 1107.2, 1118.2, 1229.8, 1248.9, 1286.9, 1302.7, 1312.7, 1379.8, 1398.8, 1427.4, 1475.2, 1490.6, 1514.3, 1540.7, 1632.1, 1780.5, 1792.4, 2818.5, 3021.2, 3062.9, 3076.4, 3094.6, 3118.9, 3149.3, 3188.7, 3618.1

B: 2.1310897 1.0246285 0.8497782

C	-0.277613	1.084755	1.038480
C	-0.555474	-0.410606	1.187878
N	-0.874127	-1.038018	-0.075763
C	-2.041624	-0.790908	-0.703260
C	0.572975	1.983589	-0.946638
N	0.793314	1.280165	0.073843
O	-2.928154	-0.097348	-0.249237
O	2.101593	-1.505047	-0.367477
O	2.794635	-0.472548	0.029226
H	0.054160	1.477688	2.000397
H	-1.189279	1.602217	0.731554
H	-1.392421	-0.541020	1.872484
H	0.317991	-0.916018	1.595526
H	-0.120361	-1.493005	-0.568415
H	-2.122963	-1.292066	-1.679445
H	1.357197	2.108055	-1.687708
H	-0.385909	2.476622	-1.117804
H	2.113097	0.281073	0.107997

$CHONHCH_2CH_2N{=}CH_2$

$\tilde{\nu}$: 28.2, 103.6, 107.4, 238.6, 249.6, 334.0, 419.5, 545.0, 595.4, 729.8, 778.4, 861.4, 916.6,1047.4, 1065.9, 1091.6, 1093.3, 1123.1, 1210.7, 1240.4, 1254.9, 1299.4, 1375.7, 1393.1, 1428.1, 1483.9, 1506.9, 1512.7, 1539.7, 1779.8, 1804.0, 3010.2, 3024.5, 3038.1, 3052.6, 3097.2, 3140.3, 3170.7, 3640.6

B: 10.4915557 1.1397915 1.0691865

C	-1.124705	0.731033	0.438431
C	0.183540	0.678421	-0.335659
N	0.898268	-0.538211	-0.015742
C	2.243968	-0.590684	0.081433
C	-3.099532	-0.312923	-0.218746
N	-1.905471	-0.458897	0.145985
O	2.988288	0.351758	-0.073595
H	-1.666636	1.652975	0.199317
H	-0.899352	0.722961	1.506594
H	0.822518	1.519154	-0.073533
H	-0.022077	0.733438	-1.407948
H	0.354560	-1.378919	0.095703
H	2.611004	-1.598624	0.329400
H	-3.699766	-1.188798	-0.448859
H	-3.575761	0.668422	-0.316371

HO_2

$\tilde{\nu}$: 1252.8, 1459.4, 3686.8

B: 628.5431720 34.6992321 32.8838526

H	-0.880747	-0.865418	0.000000
O	0.055047	0.708193	0.000000
O	0.055047	-0.600015	0.000000

Table S6.

Quantum chemistry results for the $\dot{C}H_2NHCH_2OH + O_2$ reaction.Electronic energies of reactants, intermediates and products (/Hartree), and relative energies including Zero Point Energies, $\Delta E_{v=0}$ (/kJ mol^{-1}), of stationary points on the potential energy surface.

	M06-2X/aug-cc-pVTZ		
Species	E_{Elec}	E_{ZPE}	$\Delta E_{v=0}$
$\dot{C}H_2NHCH_2OH$	-209.723072	0.085027	
O_2	-150.324795	0.003997	
Sum reactants	-360.047868	0.089024	0
$\dot{O}OCH_2NHCH_2OH$	-360.104919	0.096067	-131.3
SP21	-360.059069	0.089496	-28.2
CH_2=NCH_2OH•HOO	-360.086844	0.092347	-93.6
CH_2=NCH_2OH	-209.155531	0.074719	
HO_2	-150.908095	0.014578	
Sum products	-360.063625	0.089297	-40.7

Table S6, continued. Vibrational frequencies ($\tilde{\upsilon}$/cm^{-1}), Rotational constants (B /GHZ) and Cartesian Coordinates (/Å) of the species listed above.

$\dot{C}H_2NHCH_2OH$	C	1.540076	-0.454401	0.094562
$\tilde{\upsilon}$: 164.4, 285.3, 361.0, 422.3, 544.7, 637.8, 682.8, 971.8,	N	0.655893	0.558275	-0.238051
1007.8, 1071.5, 1175.8, 1252.8, 1320.0, 1374.7, 1429.1,	C	-0.662545	0.498151	0.331653
1471.4, 1515.0, 1530.0, 3058.1, 3145.7, 3168.8, 3286.1,	O	-1.473553	-0.518055	-0.217548
3602.3, 3843.5	H	2.450396	-0.529811	-0.477111
B: 18.3019307 5.3723517 4.7777199	H	1.540422	-0.768841	1.128653
	H	0.662075	0.826387	-1.211227
	H	-1.184020	1.433160	0.143621
	H	-0.544611	0.361908	1.410068
	H	-0.992277	-1.348787	-0.144556
O_2	O	0.000000	0.000000	0.594925
$\tilde{\upsilon}$: 1754.5, B: 44.6355338	O	0.000000	0.000000	-0.594925
$\dot{O}OCH_2NHCH_2OH$	C	0.673404	1.044222	0.258853
	N	-0.493561	0.944706	-0.513024
$\tilde{\upsilon}$: 75.7, 114.9, 157.8, 327.7, 377.7, 450.4, 506.0, 619.1,	C	-1.614282	0.249007	0.068761
714.9, 824.5, 971.3, 1001.7, 1086.3, 1173.6, 1213.2,	O	-1.522887	-1.157197	0.048702
1235.7, 1302.3, 1342.0, 1380.1, 1402.7, 1441.2, 1487.3,	O	1.300747	-0.260687	0.573669
1516.4, 1524.2, 3051.7, 3106.0, 3142.6, 3175.2, 3584.2,	O	1.784257	-0.804299	-0.501419
3862.3	H	1.441344	1.619397	-0.251201
B: 5.8532125 2.7048332 2.1562686	H	0.458664	1.446154	1.247580
	H	-0.310207	0.663313	-1.467019
	H	-2.510016	0.487821	-0.499002
	H	-1.733928	0.623595	1.089894
	H	-0.742596	-1.435142	0.537610
SP21	C	0.576376	1.420899	0.089472
$\tilde{\upsilon}$: -993.7, 47.2, 131.4, 199.8, 245.0, 399.1, 445.7, 489.7,	N	-0.296390	0.737824	-0.582328
561.6, 677.2, 818.8, 994.3, 1055.7, 1094.1, 1145.8,	C	-1.540210	0.361066	0.097988
1233.0, 1259.3, 1323.1, 1391.8, 1418.1, 1444.9, 1499.2,	O	-1.699800	-1.023675	0.141372
1527.5, 1659.2, 1857.7, 3018.0, 3128.4, 3134.5, 3236.5,	O	1.762075	-0.339960	0.514127
3847.6	O	1.265158	-1.056048	-0.416490

B: 5.2763271 2.7641776 2.0525525	H 1.467182 1.779621 -0.408235<br>H 0.345200 1.826240 1.071175<br>H 0.336977 -0.239120 -0.881855<br>H -2.379297 0.744530 -0.478010<br>H -1.556819 0.808408 1.099465<br>H -0.974970 -1.418777 0.636921
CH_2=NCH_2OH•HOO<br><br>$\tilde{\nu}$: 57.4, 93.3, 135.5, 146.2, 194.9, 282.5, 442.0, 518.5, 631.0, 729.1, 873.8, 971.6, 1070.9, 1109.5, 1166.4, 1224.9, 1297.6, 1306.4, 1410.6, 1452.5, 1505.0, 1517.1, 1662.8, 1788.6, 2856.6, 3037.0, 3071.9, 3092.3, 3186.0, 3703.6<br>B: 4.4462311 2.3505580 1.6180418	C 1.886433 -1.219418 -0.165098<br>N 0.852515 -0.539495 0.057860<br>C 0.996342 0.870595 0.431498<br>O 0.263874 1.674034 -0.429139<br>O -2.151959 0.183806 0.058007<br>O -1.764607 -1.060354 0.003213<br>H 1.795183 -2.265752 -0.441029<br>H 2.889853 -0.794271 -0.093396<br>H -0.749434 -1.009647 -0.021519<br>H 2.040938 1.184427 0.371129<br>H 0.649883 0.952619 1.466577<br>H -0.669144 1.422133 -0.341827
CH_2=NCH_2OH<br>$\tilde{\nu}$: 225.4, 340.1, 348.5, 644.0, 690.4, 967.8, 1022.7, 1096.0, 1101.2, 1234.9, 1252.6, 1382.1, 1406.5, 1487.7, 1501.6, 1774.8, 3064.4, 3080.0, 3121.4, 3178.3, 3877.2<br><br>B: 18.5080192 6.4959234 5.0313707	C -1.411815 0.466332 -0.020698<br>N -0.796149 -0.628577 0.047282<br>C 0.647914 -0.635771 -0.012712<br>O 1.291493 0.612602 -0.092321<br>H -2.497210 0.457338 0.019699<br>H -0.910787 1.429410 -0.129812<br>H 0.926742 -1.185664 -0.911053<br>H 0.997486 -1.204773 0.851861<br>H 1.308271 1.019543 0.777365
HO_2<br>$\tilde{\nu}$: 1252.8, 1459.4, 3686.8<br>B: 628.5431720 34.6992321 32.8838526	H -0.880747 -0.865418 0.000000<br>O 0.055047 0.708193 0.000000<br>O 0.055047 -0.600015 0.000000

Atmospheric photo-oxidation of 1-nitropiperazine from first principles.

Our previous study of the CH_3NHNO_2 and $(CH_3)_2NNO_2$ reactions with OH radicals showed that the electron withdrawing NO_2-group lowers the reactivity of the adjacent groups by a factor of around 20. The initial step in the OH reaction with 1-nitropiperazine is therefore expected to H-abstraction from “the other end of the molecule”:

$\Delta H^{\ominus}$ = -104 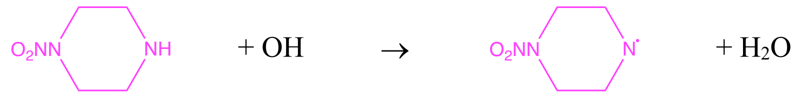

(25a)

$\Delta H^{\ominus}$ = -115 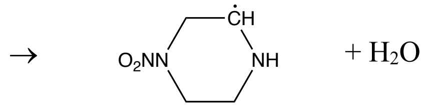

(25b)

The 1-nitropiperazin-1-yl radical will undergo the same reactions as outlined above for the piperazinyl radical in reactions (2) – (7): formation of the corresponding imine, the mixed nitrosamine-nitramine and the di-nitramine.

The alkyl radical, 1-nitropiperazin-2-yl, will undergo the same reactions leading to imine formation and ring-opening as outlined above for piperazin-2-yl in reactions (8) – (11):

$\Delta H^{\ominus}$ = -123 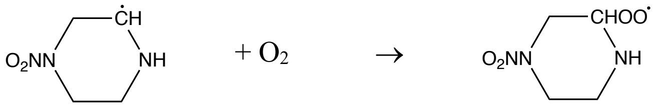

(26)

$\Delta H^{\ominus}$ = +70 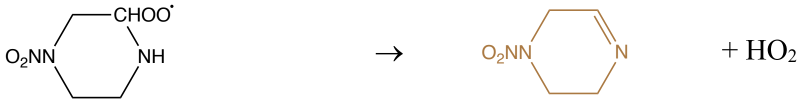

(27a)

$\Delta H^{\ominus}$ = +70 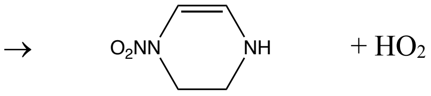

(27b)

$\Delta H^{\ominus}$ = +27 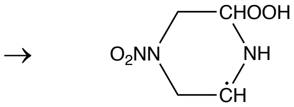

(27c)

$\Delta H^{\ominus}$ = -60 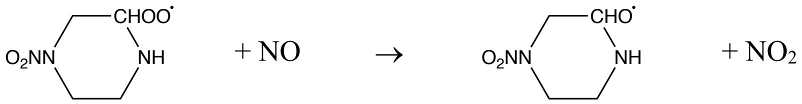

(28)

$\Delta H^{\ominus}$ = -87 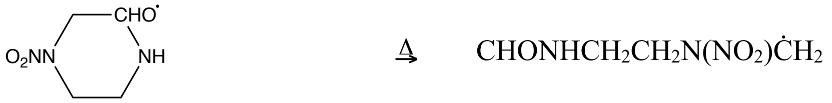

(29)

The barriers to reactions (27a) – (27c) are similar to to those of reaction (9a) – (9c), and only the imine-route will compete with the NO reaction leading to the allkoxy radical. Again, the alkoxy radical is found to be metastable with a barrier of around 10 kJ mol^{-1} to ring opening. Further, the N-nitro alkyl radical formed upon ring opening is found with a barrier of around xx kJ mol^{-1} to NO_2 ejection.

$\Delta H^{\ominus}$ = -45 $\quad CHONHCH_2CH_2N(NO_2)\dot{C}H_2 \rightarrow CHONHCH_2CH_2N{=}CH_2 + NO_2$ (30)

Figure S6 illustrates the PES of reactions (29) – (30) (the underlying quantum chemistry results are collected in Table S6)

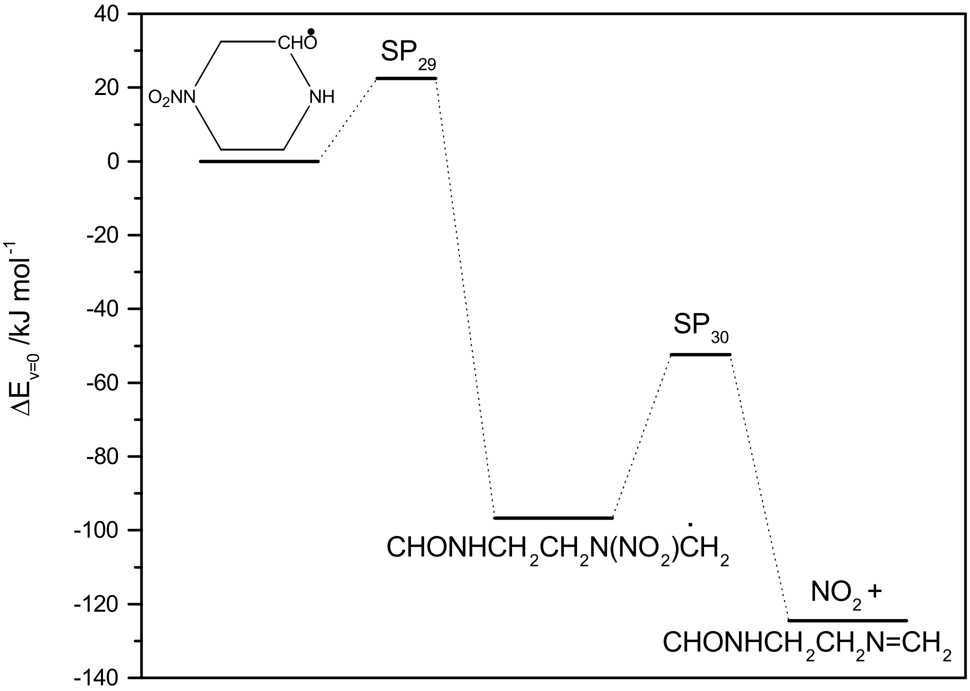

Figure S6.
Stationary points on the PES for ring-opening of the N(NO$_2$)CH$_2$CH$_2$NHCH$_2$CHȮ radical and the subsequent NO$_2$-ejection. Results from M06-2X/aug-cc-pVTZ calculations.

The atmospheric fate of the CHONHCH$_2$CH$_2$N(NO$_2$)ĊH$_2$ radical was investigated in master equation calculations based on the PES illustrated in Figure S6. The competing reactions (H-abstraction from the piperazine alkoxy radical by O$_2$, and O$_2$ addition to the CHONHCH$_2$CH$_2$N(NO$_2$)ĊH$_2$ radical) were included with rate coefficients of respectively 1×10^{-11} and 4×10^{-12} cm^3 molecule^{-1} s^{-1}. The calculations show a CHONHCH$_2$CH$_2$N=CH$_2$ yield of > 99.5%. The major routes in the atmospheric degradation of 1-nitropiperazine will consequently be as follows:

Scheme S1.

Quantum chemistry prediction of the major primary products in the atmospheric photo-oxidation of 1-nitropiperazine ($PZNO_2$).

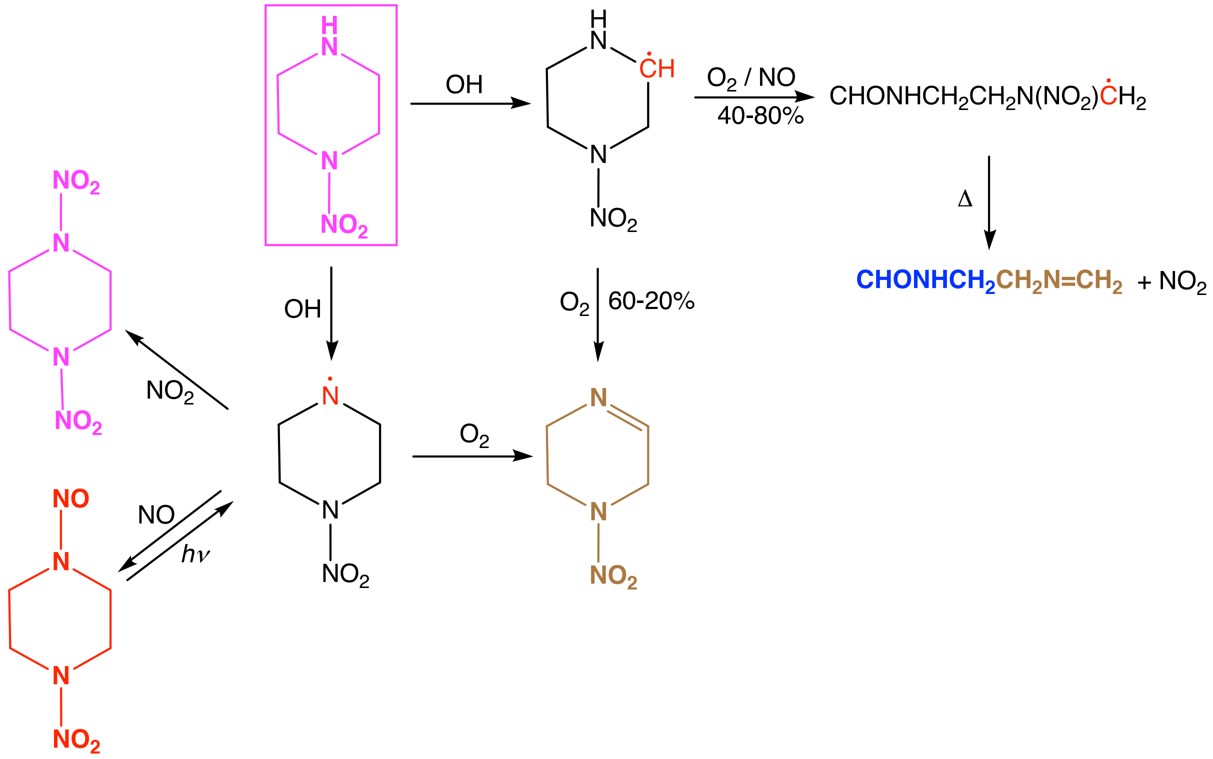

Table S7.

Quantum chemistry results for the $N(NO_2)CH_2CH_2NHCH_2C\dot{H}O$ → $CHONHCH_2CH_2N{=}CH_2$ + NO_2 reaction. Electronic energies of reactants, intermediates and products (/Hartree), and relative energies including Zero Point Energies, $\Delta E_{v=0}$ (/kJ mol^{-1}), of stationary points on the potential energy surface.

	M06-2X/aug-cc-pVTZ		
Species	E_{Elec}	E_{ZPE}	$\Delta E_{v=0}$
$N(NO_2)CH_2CH_2NHCH_2CH\dot{O}$	-546.969930	0.142009	
SP29	-546.960023	0.140682	22.5
$CHONHCH_2CH_2N(NO_2)\dot{C}H_2$	-547.004441	0.139693	-96.7
SP30	-546.985193	0.137429	-52.1
$CHONHCH_2CH_2N{=}CH_2$	-341.935922	0.126056	
NO_2	-205.074635	0.009168	
Sum products	-547.010557	0.135223	-124.5

Table S7, continued. Vibrational frequencies ($\tilde{\nu}$/cm^{-1}), Rotational constants (B /GHZ) and Cartesian Coordinates (/Å) of the species listed above.

$N(NO_2)CH_2CH_2NHCH_2CH\dot{O}$

$\tilde{\nu}$: 85.6, 97.1, 135.5, 246.0, 302.4, 380.9, 414.0, 426.3, 464.6, 503.2, 585.9, 624.3, 655.0, 732.1, 795.0, 824.5, 867.8, 897.5, 927.3, 991.4, 1039.2, 1066.7, 1105.1, 1110.4, 1159.5, 1180.8, 1201.7, 1272.3, 1294.4, 1321.0, 1338.4, 1356.6, 1375.2, 1390.9, 1404.9, 1465.9, 1469.7, 1494.9, 1508.5, 1672.8, 2806.8, 2991.8, 3112.4, 3124.0, 3126.4, 3200.2, 3219.5, 3568.3

B: 2.2200000 0.0076400 1.1931068

C	-0.420640	1.443895	0.710756
C	0.505218	1.724538	-0.463090
N	1.717086	0.928411	-0.297770
C	1.469001	-0.500212	-0.272687
C	0.498674	-0.816595	0.895910
N	-0.696401	0.005066	0.784805
O	2.574919	-1.248067	-0.177928
N	-1.644454	-0.455097	-0.116246
O	-1.600908	-1.628070	-0.418885
O	-2.474138	0.339889	-0.499198
H	-1.358515	1.980072	0.636278
H	0.073186	1.701235	1.646282
H	0.774001	2.779367	-0.474539
H	-0.031875	1.503032	-1.396461
H	2.412126	1.142615	-1.001164
H	0.953555	-0.869829	-1.191984
H	0.989832	-0.537689	1.825529
H	0.241568	-1.867243	0.903286

SP29

$\tilde{\nu}$: -461.0, 92.7, 118.9, 148.6, 230.0, 258.7, 346.5, 396.6, 435.2, 480.6, 504.5, 565.1, 646.2, 662.0, 786.7, 822.8, 833.8, 851.6, 894.6, 951.5, 973.1, 987.0, 1026.1, 1090.8, 1151.7, 1159.6, 1209.0, 1275.4, 1296.2, 1316.8, 1337.8, 1367.5, 1377.3, 1389.1, 1451.4, 1470.7, 1483.9, 1500.6, 1517.4, 1697.2, 3031.3, 3058.1, 3132.9, 3145.5, 3174.5, 3196.3, 3277.1, 3631.3

B: 2.4200000 0.0083696 1.2163926

C	0.074254	1.078728	1.055171
C	0.718255	1.573119	-0.265200
N	0.873157	0.421825	-1.131322
C	1.613358	-0.649945	-0.571680
C	0.341626	-1.293829	0.680876
N	-0.551453	-0.227080	0.838852
O	2.564938	-0.441471	0.240283
H	-0.662692	1.778531	1.430643
H	0.831569	0.905540	1.814556
H	1.664037	2.065483	-0.026390
H	0.056567	2.280824	-0.757495
H	1.042741	0.603892	-2.107290
H	1.664217	-1.508874	-1.251813
H	0.933775	-1.467969	1.567214
H	-0.101062	-2.166914	0.224868

	N	-1.634885	-0.205182	-0.046965
	O	-1.978646	-1.256725	-0.530170
	O	-2.176522	0.864959	-0.199270
$CHONHCH_2CH_2N(NO_2)\dot{C}H_2$<br><br>$\tilde{\nu}$: 39.2, 85.7, 99.7, 155.3, 176.0, 231.8, 289.4, 349.5, 388.8, 416.2, 459.8, 471.7, 492.8, 539.8, 632.0, 650.4, 736.3, 809.3, 845.1, 872.7, 967.1, 1015.9, 1046.1, 1053.1, 1095.8, 1215.2, 1247.6, 1305.4, 1325.6, 1337.0, 1386.8, 1401.8, 1427.4, 1431.9, 1468.7, 1486.9, 1507.9, 1543.6, 1639.3, 1797.5, 3028.0, 3086.1, 3129.0, 3165.7, 3193.6, 3230.8, 3374.8, 3667.7<br><br>B: 2.5700000 0.0096129 0.9086783	C	-0.108835	-0.529596	1.121098
	C	-0.884592	-1.388465	0.121037
	N	-1.496906	-0.609179	-0.936990
	C	-2.422921	0.339896	-0.681585
	C	0.446074	1.749434	0.352921
	N	0.769657	0.435021	0.469331
	O	-2.822445	0.613817	0.429914
	H	0.487667	-1.169912	1.765240
	H	-0.798440	0.057483	1.718467
	H	-1.656743	-1.920815	0.678390
	H	-0.218436	-2.112789	-0.337671
	H	-1.191572	-0.739703	-1.885540
	H	-2.784096	0.849710	-1.586589
	H	-0.475152	2.055822	0.813911
	H	1.129710	2.401126	-0.154363
	N	1.899809	-0.041173	-0.123933
	O	2.606100	0.765217	-0.703804
	O	2.106443	-1.246686	-0.020051
SP30<br><br>$\tilde{\nu}$: -700.8, 40.0, 64.1, 95.7, 159.5, 163.5, 175.0, 262.2, 290.8, 300.0, 423.5, 452.0, 504.1, 517.7, 620.0, 678.6, 785.8, 809.9, 853.5, 885.3, 917.2, 1023.3, 1049.3, 1056.1, 1103.8, 1200.6, 1242.3, 1264.5, 1300.4, 1309.6, 1374.4, 1398.6, 1427.9, 1436.7, 1485.2, 1489.6, 1544.4, 1567.8, 1625.4, 1796.1, 3030.7, 3089.2, 3108.1, 3138.5, 3159.2, 3173.6, 3267.1, 3663.5<br><br>B: 2.4200000 0.0089518 0.9132536	C	-0.156428	-0.564511	1.239913
	C	-0.766473	-1.383845	0.094249
	N	-1.300782	-0.568441	-0.976838
	C	-2.329934	0.282861	-0.781402
	C	0.286927	1.712670	0.583366
	N	0.701077	0.505670	0.761442
	O	-2.880760	0.443976	0.286962
	H	0.440029	-1.226636	1.864640
	H	-0.946259	-0.107715	1.830443
	H	-1.564613	-1.992261	0.520769
	H	-0.011251	-2.038798	-0.329480
	H	-0.861483	-0.594347	-1.880826
	H	-2.623507	0.826873	-1.691105
	H	-0.735261	1.977597	0.827444
	H	0.984103	2.429355	0.172679
	N	1.847305	-0.049097	-0.285290
	O	2.388366	0.845495	-0.894184
	O	2.289956	-1.136226	0.028908
$CHONHCH_2CH_2N{=}CH_2$<br><br>$\tilde{\nu}$: 28.2, 103.6, 107.4, 238.6, 249.6, 334.0, 419.5, 545.0, 595.4, 729.8, 778.4, 861.4, 916.6, 1047.4, 1065.9, 1091.6, 1093.3, 1123.1, 1210.7, 1240.4, 1254.9, 1299.4, 1375.7, 1393.1, 1428.1, 1483.9, 1506.9, 1512.7, 1539.7, 1779.8, 1804.0, 3010.2, 3024.5, 3038.1, 3052.6, 3097.2, 3140.3, 3170.7, 3640.6<br><br>B: 10.4915557 1.1397915 1.0691865	C	-1.124705	0.731033	0.438431
	C	0.183540	0.678421	-0.335659
	N	0.898268	-0.538211	-0.015742
	C	2.243968	-0.590684	0.081433
	C	-3.099532	-0.312923	-0.218746
	N	-1.905471	-0.458897	0.145985
	O	2.988288	0.351758	-0.073595
	H	-1.666636	1.652975	0.199317
	H	-0.899352	0.722961	1.506594
	H	0.822518	1.519154	-0.073533
	H	-0.022077	0.733438	-1.407948
	H	0.354560	-1.378919	0.095703
	H	2.611004	-1.598624	0.329400
	H	-3.699766	-1.188798	-0.448859
	H	-3.575761	0.668422	-0.316371
NO_2<br>$\tilde{\nu}$: 783.5, 1465.2, 1775.4<br><br>B: 253.9647127 13.2906276 12.6296837	N	0.000000	0.314443	0.000000
	O	1.090260	-0.137566	0.000000
	O	-1.090260	-0.137572	0.000000

Piperazine + OH reaction kinetics

In relative-rate kinetic studies the removals of the reacting species are measured simultaneously as a function of reaction time. Assuming that the compounds under study react solely with the same radical species and that none of the compounds are reformed in any side reactions, the relative rate coefficient, k_{rel}, is given according to the following expression:

$$\ln\left\{\frac{[S]_0}{[S]_t}\right\} = k_{rel} \cdot \ln\left\{\frac{[R]_0}{[R]_t}\right\} \;\; ; \;\; k_{rel} = \frac{k_S}{k_R} \tag{I}$$

where $[S]_0$, $[R]_0$, $[S]_t$ and $[R]_t$ are concentrations of substrate and the reference compound at start and at the time t, respectively, and k_S and k_R are the corresponding rate coefficients for reaction with OH. A plot of ln *vs* $\ln\{[R]_0/[R]_t$ will thus give the relative reaction rate coefficient $k_{rel} = k_F/k_R$ as the slope. The kinetic analysis according to eq. (I) assumes that no other loss processes are taking place. Unless corrected for, additional gas-phase or surface reactions with other species, loss to particles and wall surfaces, and dilution will cause systematic errors in the relative rate determination. In the EUPHORE experiments purified air is constantly added to compensate for leakage and continuous sampling by the air monitors. This is corrected for in the data analysis using the loss of the virtually inert CH_3CN ($k_{OH+CH_3CN} = 2.2 \times 10^{-14}$ cm^3 $molecule^{-1}$ s^{-1} at 298 K)[19] of which about 100 ppb was added to the chamber together with the other reactants. The apparent dilution rate coefficient of the chamber, $k_{dilution}$, is determined from:

$$\ln\left\{[\mathrm{CH_3CN}]_0 / [\mathrm{CH_3CN}]_t\right\} = k_{dilution} \cdot t \tag{II}$$

where $[CH_3CN]_0$ and $[CH_3CN]_t$ are the CH_3CN concentrations at times zero and t, respectively. In addition, non-negligible loss of PA and the reference compounds due to adsorption on walls was observed prior to opening the chamber canopy, $k_{wall} = 1 - 2 \times 10^{-5}$ s^{-1}, and the relative rate experiments were therefore initially analyzed according to:

$$\ln\left\{\frac{[S]_0}{[S]_t}\right\} - (k_{wall,S} + k_{dilution}) \cdot t = k_{rel} \cdot \left\{\ln\left\{\frac{[R]_0}{[R]_t}\right\} - (k_{wall,R} + k_{dilution}) \cdot t\right\} \tag{III}$$

Figure S7 shows the time evolutions of piperazine, isoprene, 1,3-5-trimethylbenzene, limonene and the inert tracer (CH_3CN) during the first of two kinetic experiments (the second kinetic experiment is documented in the main text). The dilution loss due to chamber air replenishment can be seen from the CH_3CN signal; the apparent losses of piperazine, and the three reference compounds before opening the chamber are slightly larger than the dilution loss.

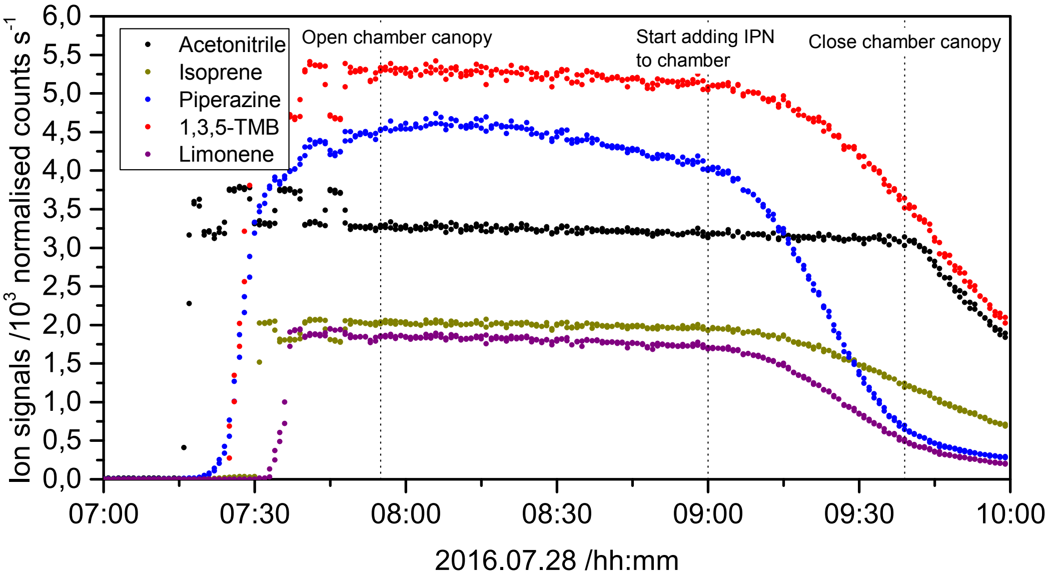

Figure S7.
Time evolution of the acetonitrile, isoprene, piperazine, 1,3,5-trimethylbenzene and limonene ion signals m/z 42.034, 68.063, 87.092, 120.094 and 137.133, respectively.

Piperazine has a high surface affinity that may affect instrument response time. Figure S8 documents a very good response time of the PTR-ToF-MS compared to a home-built high temperature PTR-MS.

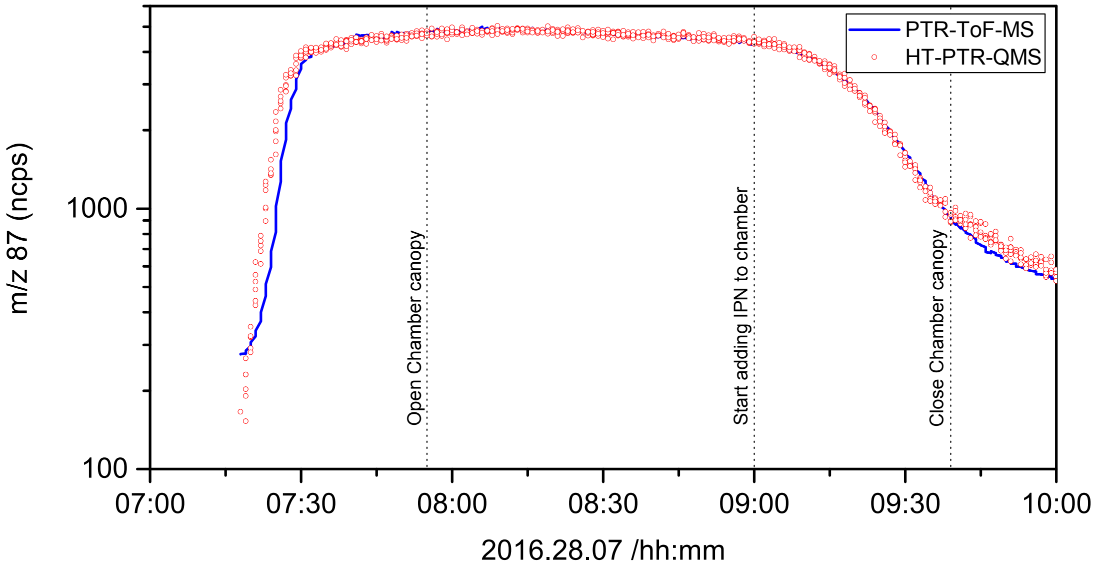

Figure S8.
Time evolution of the piperazine ion signals m/z 87.092 (PTR-ToF-MS) and 87 (HT-PTR-QMS).

The decays of PZ and the reference compounds are plotted according to equation III in Figure S9(A). Least–squares fitting of the loss-corrected data resulted in the following relative rates (2σ statistical error limits): $k_{OH+Piperazine}/k_{OH+Pyrrole} = 2.545 \pm 0.026$, $k_{OH+Piperazine}/k_{OH+Isoprene} = 3.91 \pm 0.17$, $k_{OH+Piperazine}/k_{OH+1,3,5\text{-}Trimethylbenzene} = 5.52 \pm 0.28$, and $k_{OH+Piperazine}/k_{OH+Limonene} = 1.502 \pm 0.021$ (experiment 1) and 1.645 ± 0.019 (experiment 2). Using $k_{OH+Pyrrole} = 1.23 \times 10^{-10}$,[20-22] $k_{OH+Isoprene} = 0.966 \times 10^{-10}$,[23] $k_{OH+1,3,5\text{-}Trimethylbenzene} = 5.5 \times 10^{-11}$,[24-30] and $k_{OH+Limonene} = 1.62 \times 10^{-10}$ cm^3 molecule^{-1} s^{-1} at 307 K [31-33] (all with estimated uncertainty factors of 1.20) places our absolute values for k_{OH+PZ} at 3.13, 3.78, 3.04, 2.43 and 2.67 × 10^{-10} cm^3 molecule^{-1} s^{-1}, respectively. Assuming that there are no additional molecule

specific systematic errors, we derive $k_{OH+Piperazine}$ = (3.0 ± 0.6) × 10^{-10} cm^3 $molecule^{-1}$ s^{-1} at 307 ± 2 K and 1014 ± 1 hPa from the present experiments.

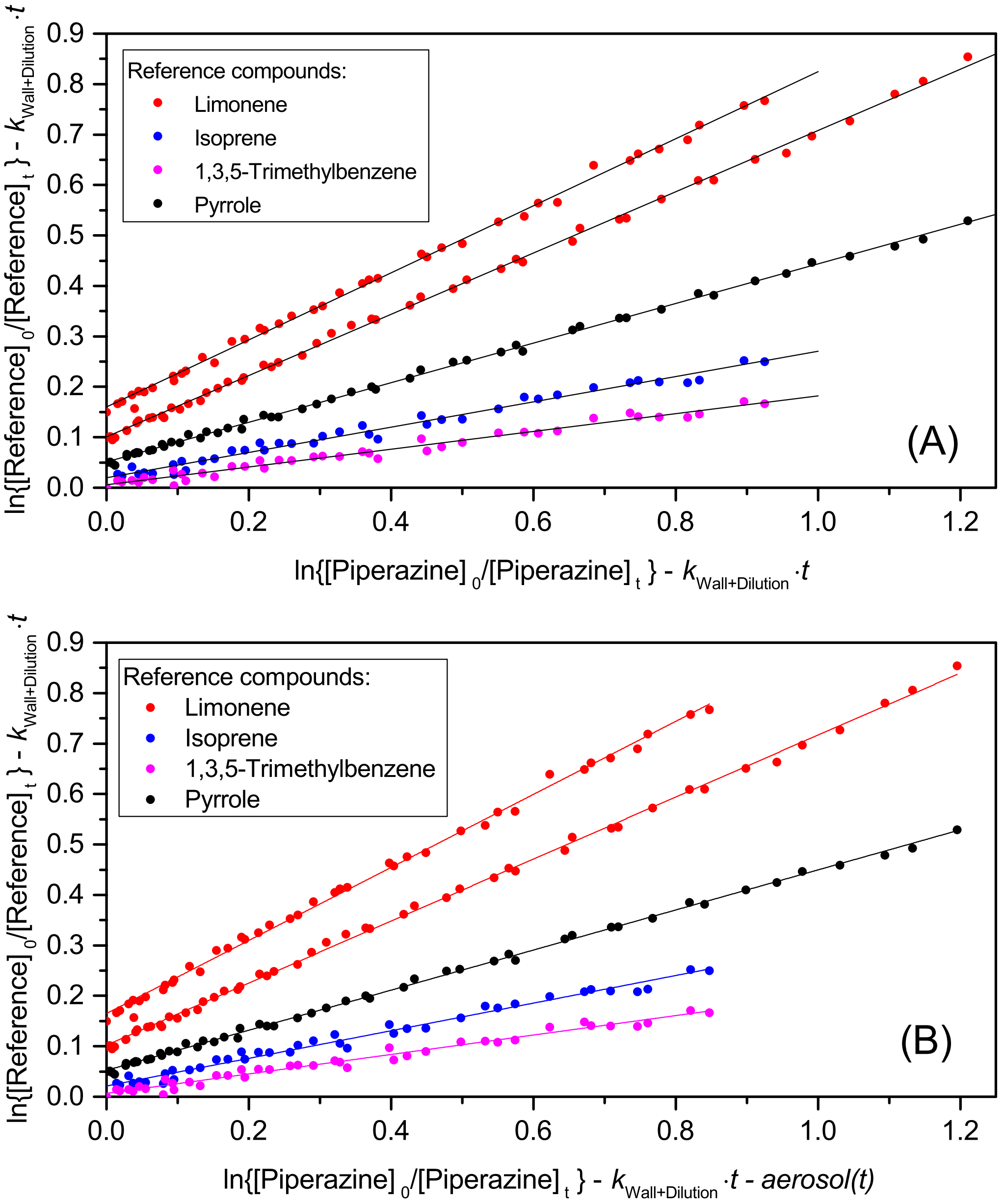

Figure S9.
Decays of piperazine and reference compounds during reaction with OH radicals. (A): Relative rate plot showing the decays of isoprene, limonene, pyrrole and piperazine at 1014 hPa and 307 K in the presence OH radicals. For the sake of clarity, the data have been displaced along the abscissa. The data have been corrected for wall loss and loss due to chamber air replenishment. (B): Relative rate plot showing the decays of isoprene, limonene, pyrrole and piperazine at 1014 hPa and 307 K in the presence OH radicals. For the sake of clarity, the data have been displaced along the abscissa. The data have been corrected for wall loss, loss due to chamber air replenishment and loss connected with aerosol formation, see text for details.

In addition to dilution and wall losses, the partitioning to particles needs to be considered. Inspection of the total particle mass during the kinetic experiments (Figure S10 and S11) reveals that about 50 % more particle mass is formed during experiment 1 than in experiment 2. Only a minor mass fraction of the particles formed during the OH initiated photo-oxidation studies consist of aminium nitrate. The

shape of the PZ content in the particles during the experiments can to a first approximation be described by a linear function corresponding to a zero-order transfer of gas phase PZ to the aerosol phase due to acid-base reaction with HNO_3:

$$\begin{aligned}\frac{d[S]}{dt} &= -k_S \cdot [S] \cdot [OH] - (k_{wall,S} + k_{dilution}) \cdot [S] - k_{aerosol,S} \\ \frac{d[R]}{dt} &= -k_S \cdot [R] \cdot [OH] - (k_{wall,R} + k_{dilution}) \cdot [R] - k_{aerosol,R}\end{aligned} \qquad \text{(IV)}$$

Elimination of [OH] from equations (IV) and rearrangement gives:

$$\frac{d[S]}{[S]} - \left(k_{wall,S} + k_{dilution} + \frac{k_{aerosol,S}}{[S]}\right) \cdot dt = k_{rel} \cdot \frac{d[R]}{[R]} - k_{rel} \cdot \left(k_{wall,R} + k_{dilution} + \frac{k_{aerosol,R}}{[R]}\right) \cdot dt \qquad \text{(V)}$$

Equation (V) has no analytical solution from which one may derive the relative rate, k_{rel}, from a simple relative rate plot. However, treating the gas phase losses of piperazine as perturbations to the major photo-oxidation losses by OH radicals allows a separation of the variables in the differential equation making it readily integrable.

$$\frac{k_{aerosol,S}}{[S]} \approx \frac{f \cdot [S]_0}{\Delta t \cdot [S]} \approx \frac{f \cdot [S]_0}{\Delta t \cdot [S]_0 \cdot e^{-k_S \langle \mathrm{OH} \rangle t}} \approx x_{aerosol,S} \cdot e^{k_S \langle \mathrm{OH} \rangle t} \qquad \text{(VI)}$$

where f is the fraction of the initial gas phase substrate that is transferred to the aerosol phase, and where we have replaced the time-dependent sample concentration by its ideal exponential decay and introduced the average OH radical concentration during the experiment.

$$\begin{aligned}&\ln\left\{\frac{[S]_t}{[S]_0}\right\} - \left(k_{waal,S} + k_{dilution}\right) \cdot t + \frac{x_{aerosol,S}}{k_S \cdot \langle [OH] \rangle} \cdot \left\{e^{k_S \cdot \langle [OH] \rangle \cdot t} - 1\right\} \\ &= k_{rel} \cdot \ln\left\{\frac{[R]_t}{[R]_0}\right\} - \left(k_{waal,R} + k_{dilution}\right) \cdot t + \frac{x_{aerosol,R}}{k_R \cdot \langle [OH] \rangle} \cdot \left\{e^{k_R \cdot \langle [OH] \rangle \cdot t} - 1\right\}\end{aligned} \qquad \text{(VIII)}$$

The decays of piperazine, isoprene, 1,3,5-trimethylbenzene, pyrrole and limonene in the presence of OH radicals in the two independent experiments are plotted in according to equation (VIII) and allowing a non-zero intercept in Figure S9(B).

Least–squares fitting of the loss-corrected data resulted in the following relative rates (2σ statistical error limits): $k_{\mathrm{OH+Piperazine}}/k_{\mathrm{OH+Pyrrole}}$ = 2.514 ± 0.027, $k_{\mathrm{OH+Piperazine}}/k_{\mathrm{OH+Isoprene}}$ = 3.59 ± 0.16, $k_{\mathrm{OH+Piperazine}}/k_{\mathrm{OH+1,3,5\text{-}Trimethylbenzene}}$ = 5.08 ± 0.25, and $k_{\mathrm{OH+Piperazine}}/k_{\mathrm{OH+Limonene}}$ = 1.379 ± 0.022 (experiment 1) and 1.625 ± 0.018 (experiment 2).

Using the abovementioned OH rate coefficients for the reference compounds places our absolute values for $k_{\mathrm{OH+PZ}}$, corrected for aerosol loss, at 3.09, 3.47, 2.79, 2.23 and 2.63 × 10^{-10} cm^3 $molecule^{-1}$ s^{-1}, respectively. Assuming that there are no additional molecule specific systematic errors, we derive $k_{\mathrm{OH+Piperazine}}$ = (2.8 ± 0.6) × 10^{-10} cm^3 $molecule^{-1}$ s^{-1} at 307 ± 2 K and 1014 ± 1 hPa from the present experiments, which is around 20 % higher than the recent results by Onel et al., [34] who reported k = (2.25 ± 0.28) × 10^{-10} cm^3 $molecule^{-1}$ s^{-1} at 307 K from flash photolysis/resonance fluorescence experiments.

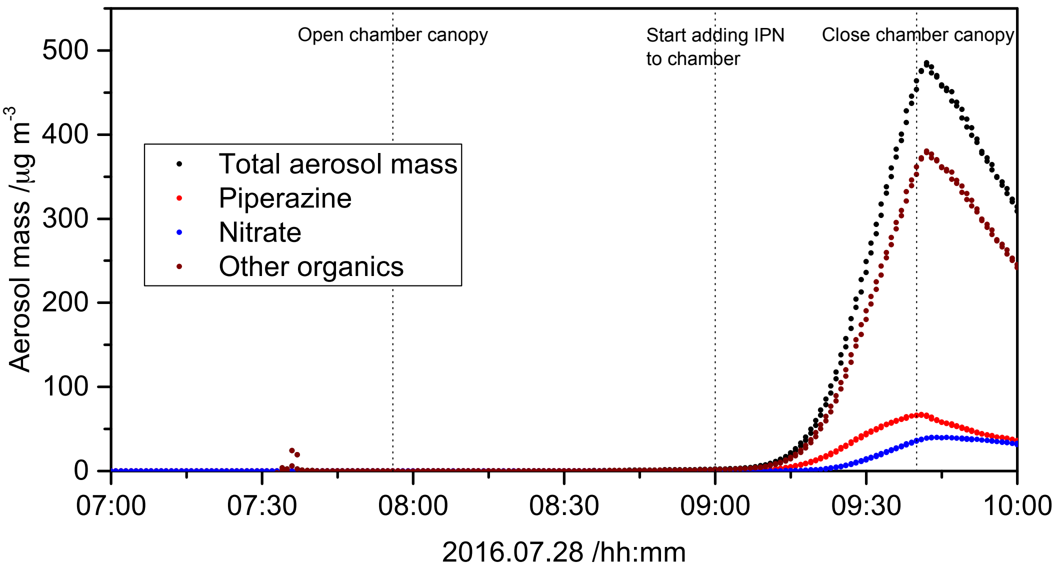

Figure S10.
Time evolution of the aerosol formed during the piperazine + OH kinetic experiment in the morning of 20.07.28. Results from AMS and CHARON PTR-ToF-MS.

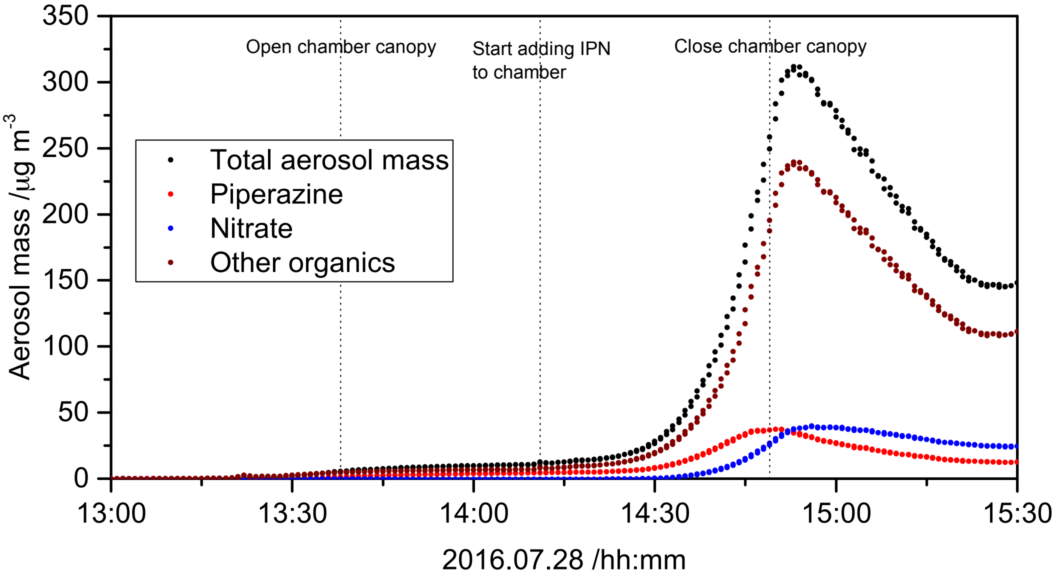

Figure S11.
Time evolution of the aerosol formed during the piperazine + OH kinetic experiment in the morning of 20.07.28. Results from AMS and CHARON PTR-ToF-MS.

1-Nitropiperazine photo-oxidation studies

Table S8.

Mass peaks detected by PTR-ToF-MS during 1-nitropiperazine ($PZNO_2$) photo-oxidation experiments. [a]

m/z 2016.09.19 High NO_2	*m/z* 2016.09.21 High NO	Ion sum formula	Interpretation and comments
42.036		$C_2H_4N^+$	
44.051 [b]	44.051	$C_2H_6N^+$	$[PZNO_2]H^+$ fragment (ring scission)
46.030	46.030	CH_4NO^+	Formamide ?
57.059	57.059	$C_3H_7N^+$	$[PZNO_2]H^+$ fragment (ring scission)
58.033	58.034	$C_2H_4NO^+$	
61.032	61.030	$C_2H_5N_2^+$	
69.045	69.045	$C_3H_5N_2^+$	Imidazole ?
72.046	72.047	$C_3H_6NO^+$	
73.071	73.061	$C_4H_9O^+$	
74.026	74.024	$C_2H_4NO_2^+$	Fragment from products
76.039		$C_2H_6NO_2^+$	
77.032		$CH_5N_2O_2^+$	
81.043	81.044	$C_4H_5N_2^+$	[Pyrazine]H^+ ?
83.060	83.060	$C_4H_7N_2^+$	[PZI]H^+ fragment
85.076	85.076	$C_4H_9N_2^+$	$[PZNO_2]H^+$ fragment
86.085	86.085	$C_4H_{10}N_2^+$	$[PZNO_2]H^+$ fragment
89.040			
97.042	97.041	$C_4H_5N_2O^+$	[Pyrazin-2(5*H*)-one]H^+ ?
99.048	99.058	$C_5H_7O_2^+$	
99.089		$C_5H_{11}N_2^+$	
101.064	101.067	$C_4H_9N_2O^+$	$[CHONHCH_2CH_2N{=}CH_2]H^+$
103.049	103.050	$C_3H_7N_2O_2^+$	
113.057		$C_4H_7N_3O^+$	
	115.073	$C_4H_9N_3O^+$	$[PZ(NO)NO_2]H^+$ fragment (NO_2 ejection)
131.071	131.073	$C_4H_9N_3O_2^+$	$[PZ(NO_2)_2]H^+$ fragment (NO_2 ejection)
132.073	132.074	$C_4H_{10}N_3O_2^+$	$[PZNO_2]H^+$
160.067	160.068	$C_5H_{10}N_3O_3^+$	$PZINO_2 + CH_2O$ condensation product
	161.064	$C_4H_9N_4O_3^+$	$[PZ(NO)NO_2]H^+$
177.055	177.056	$C_4H_9N_4O_4^+$	$[PZ(NO_2)_2]H^+$

[a] Mass peaks having an intensity change $\Delta I_{m/z} < 1$ % of $-\Delta I_{132.074}$, mass peaks originating in isotopes, and mass peaks related to well-established chamber artefacts have been omitted. [b] *m/z* in red font correspond to ion signals decreasing in intensity during the experiments.

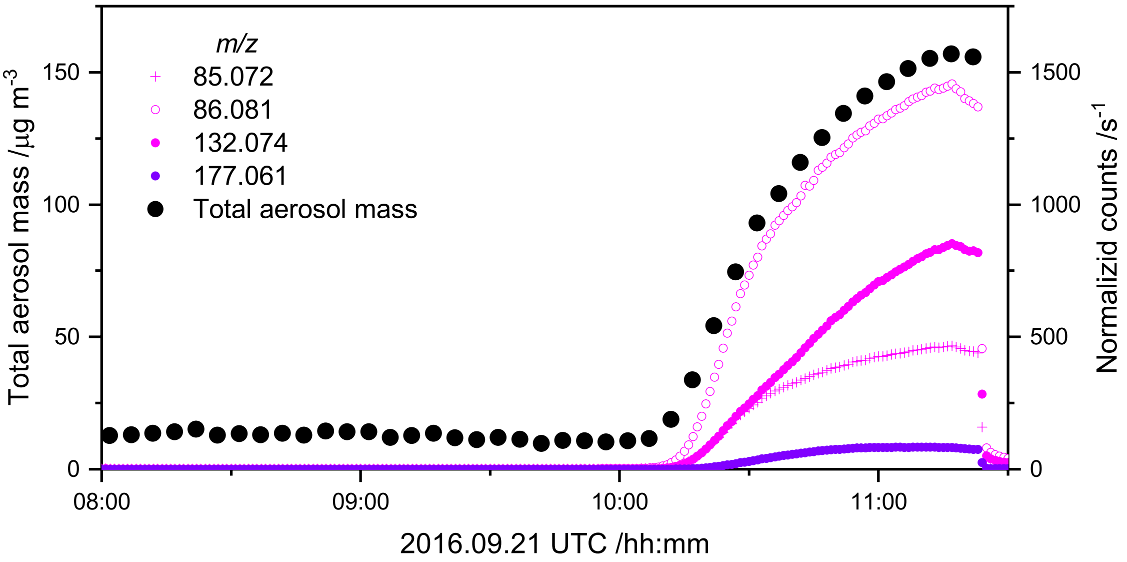

Figure S12.
Total aerosol mass and ion signals related to 1-nitropiperazine ($PZNO_2$), and 1,2-dinitropiperazine ($PZ(NO_2)_2$) observed by CHARON PTR-ToF-MS during the $PZNO_2$ photo-oxidation experiment in the afternoon of 2016.09.21. Other ion signals observed CHARON PTR-ToF-MS during the experiment include: m/z 44.047, 45.994, 46.030, 69.044, 72.041, 73.037, 73.074, 87.087, 89.071, 99.053, 99.089, 101.072, 115.082, and 117.067.

1-Nitrosopiperazine photolysis studies

The ion signals observed during the photolysis experiments are summarized in Table S7, which also includes the results reported in the ADA-2011 project.[35]

The atmospheric fate of the $HN<^{CH_2CH_2}_{CH_2CH_2}>\dot{N}$ radical was predicted in quantum chemistry calculations, see page S7. OH radicals will be generated in the 1-nitrosopiperazine photolysis from HO_2 radicals produced in the imine formation reaction. The OH radical concentration was determined indirectly during the 1-nitrosopiperazine photolysis experiments by following the amount of cyclohexanone formed from cyclohexane. The cyclohexane concentration was quantified by FTIR, whereas the cyclohexanone was determined by PTR-ToF-MS, Figure S13.

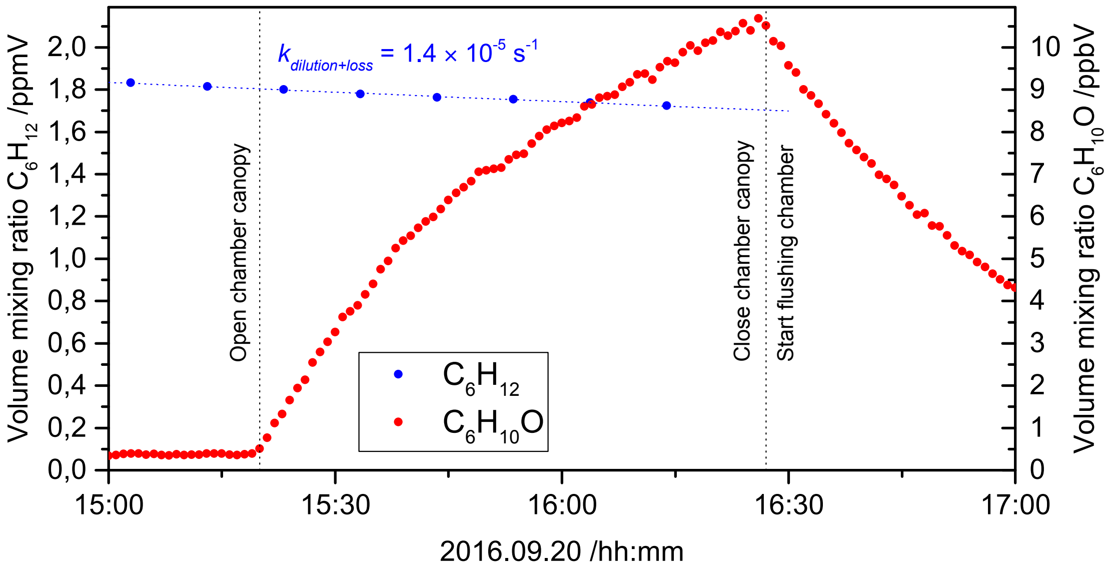

Figure S13.
Concentration profiles for C_6H_{12} (FTIR results) and $C_6H_{10}O$ (PTR-ToF-MS results) during the 1-nitrosopiperazine photolysis experiment in the afternoon of 2016.09.20.

Cyclohexanone may also react with OH radicals; the rate coefficients for OH reaction with cyclohexane and cyclohexanone are, in units of 10^{-12} cm^3 $molecule^{-1}$ s^{-1}, respectively 6.97[36] and 6.39[37] at 298 K. The OH radical concentration can then be determined from an analysis of the cyclohexanone formation rate; the differential rate law for cyclohexanone production is:

$$\frac{d\left[C_6H_{10}O\right]}{dt} = k_{OH+C6H12}\cdot\left[C_6H_{12}\right]\cdot\left[OH\right] - k_{OH+C6H10O}\cdot\left[C_6H_{10}O\right]\cdot\left[OH\right]$$

which can be solved numerically to give the OH concentration:

$$\left[OH\right]_{i+1} = \frac{\Delta\left[C_6H_{10}O\right]_i}{\Delta t\cdot\left(k_{OH+C6H12}\cdot\left[C_6H_{12}\right]_i - k_{OH+C6H10O}\cdot\left[C_6H_{10}O\right]_i\right)}$$

Figure S14 shows the OH concentration derived from the C_6H_{12} and $C_6H_{11}O$ time profiles presented in Figure S13; the NO, NO_2 and O_3 concentrations are presented in Figure S15.

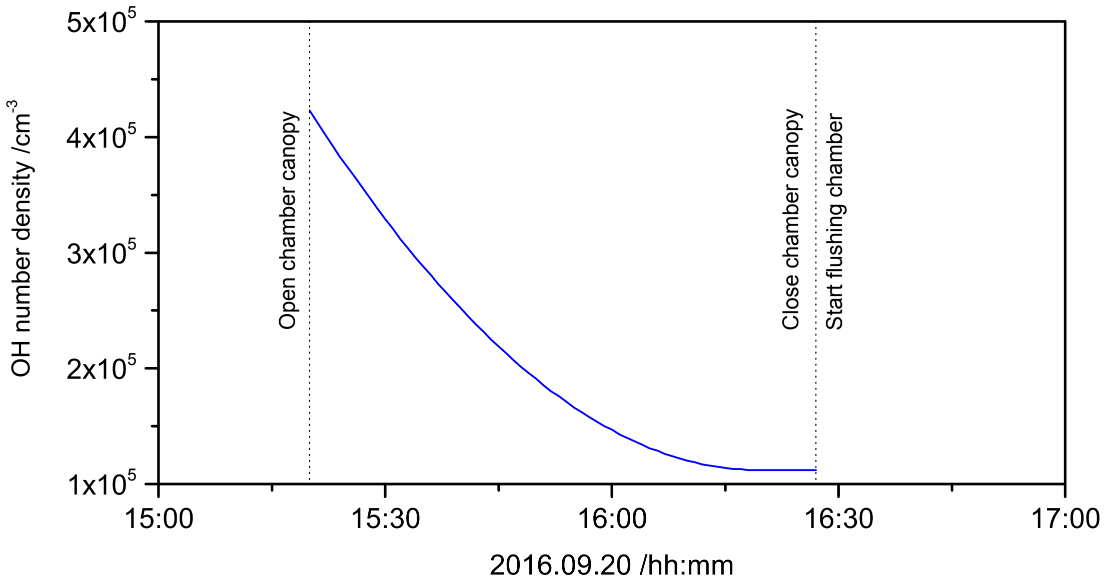

Figure S14.
Calculated OH concentration during the 1-nitrosopiperazine photolysis experiment in the afternoon of 2016.09.20.

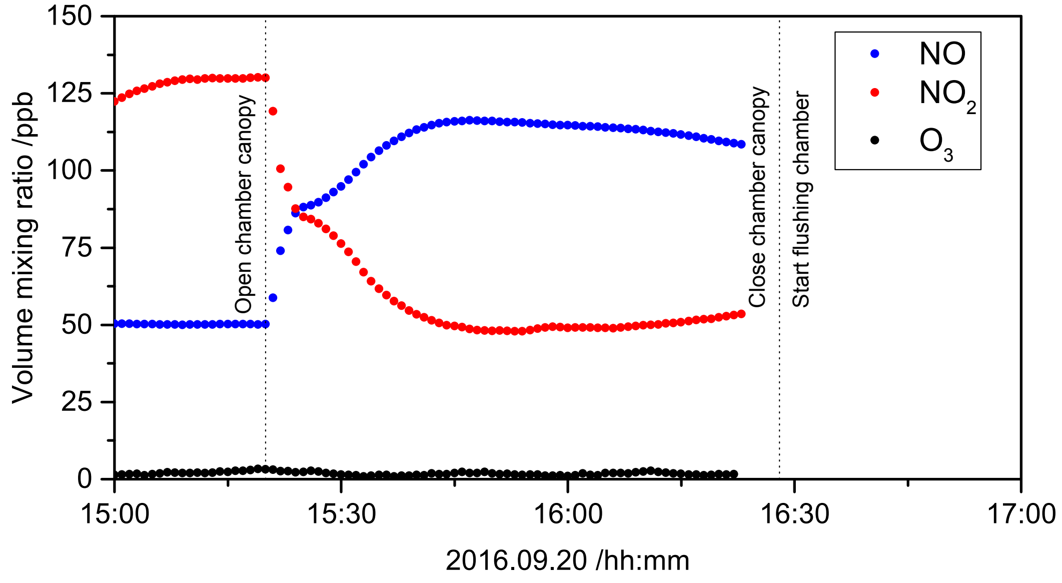

Figure S15.
NO, NO_2 and O_3 mixing ratios during during the 1-nitrosopiperazine photolysis experiment in the afternoon of 2016.09.20.

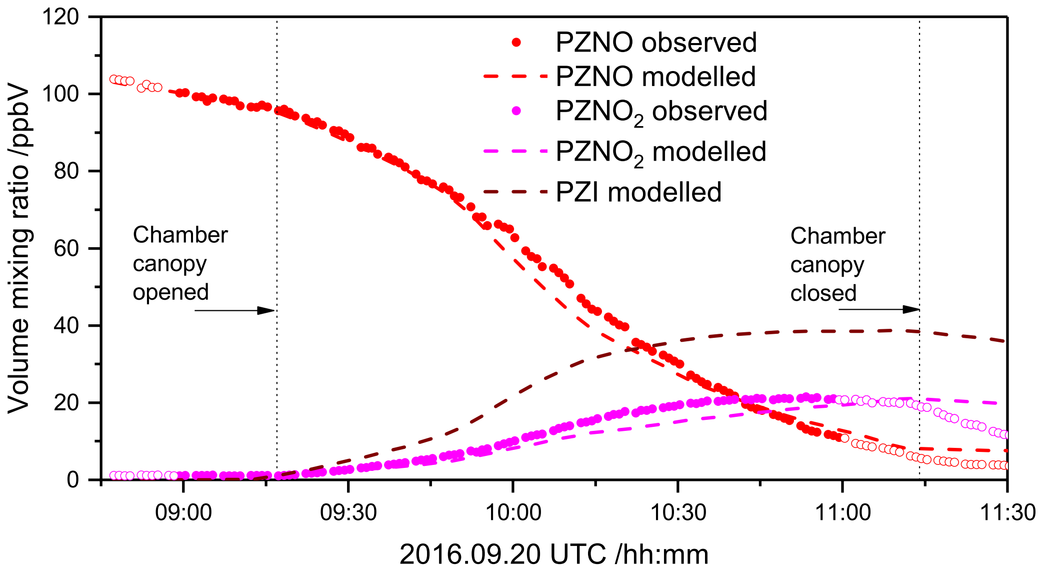

Figure S16.
Observed and modelled 1-nitrosopiperazine photolysis under natural sunlight conditions. Abbreviations: PZNO, 1-nitrosopiperazine; $PZNO_2$, 1-nitropiperazine; PZI, 1,2,3,6-tetrahydropyrazine.

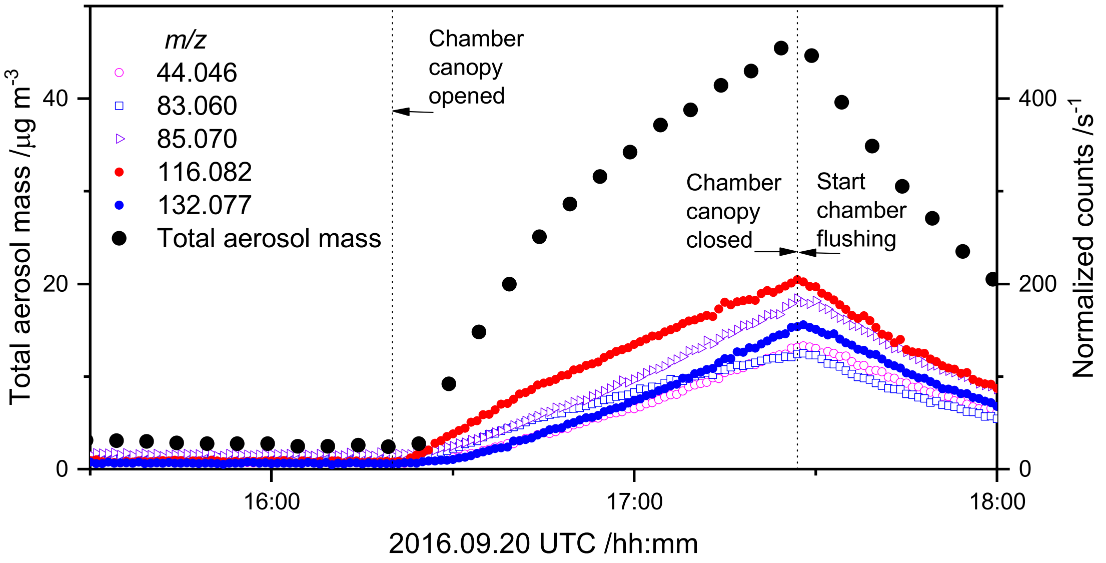

Figure S17.
Total aerosol mass and normalized ion signals related to 1-nitrosopiperazine (PZNO, *m/z* 116.086, 85.070, 44.046), 1-nitropiperazine ($PZNO_2$, *m/z* 132.072, 85.070, 44.046), and 1,2,3,6-tetrahydropyrazine (PZI, m/z 83.060, 85.070, 44.046) observed by CHARON PTR-ToF-MS during the PZNO photolysis experiment in the afternoon of 2016.09.20. Other ion signals observed CHARON PTR-ToF-MS during the experiment include: m/z 45.992, 46.029, 69.045, 71.054, 72.041, 73.071, 72.041, 73.071, 86.080, 87.090, 89.065, 99.059, 101.066, 111.049, 113.067, 115.085 and 160.076.

Table S9.
Mass peaks detected by PTR-ToF-MS during 1-nitrosopiperazine (PZNO) photolysis experiments. [a]

2010.07.19 ADA [a]	2010.07.23 ADA	2016.09.19	2016.09.20 Morning	2016.09.20 Afternoon	Ion sum formula [b]	Interpretation and comments
44.050	44.050	44.053	44.052	44.052	$C_2H_6N^+$	Fragment from ring scission of protonated PZNO, $PZNO_2$ and PZI
46.029	46.066	46.030	46.030	46.030	CH_4NO^+	Fragment, NH_2CHO, HN=CHOH, or product from OH reaction
		55.036			$C_3H_5N^+$	$[PZI]H^+$ fragment from ring scission
74.025	74.024	74.023	74.024	74.024	$C_2H_4NO_2^+$	CHONHCHO, product from OH reaction
81.045	81.045			81.045	$C_4H_5N_2^+$	Pyrazine ?
83.061	83.061	83.080	83.061	83.061	$C_4H_7N_2^+$	1,4-, 2,3- and 2,5-dihydropyrazine, $[PZI]H^+$ fragment
85.075 [c]	85.075	85.082	85.080	85.080	$C_4H_9N_2^+$	$[PZNO]H^+$ fragment; $[PZNO_2]H^+$ fragment; $[PZI]H^+$
86.085	86.084	86.085	86.085	86.085	$C_4H_{10}N_2^+$	$[PZNO]H^+$ fragment; $[PZNO_2]H^+$ fragment
			97.045	97.045	$C_4H_5N_2O^+$	
		101.062				$CHONHCH_2CH_2N=CH_2$, product from OH reaction
116.082	116.082	116.081	116.079	116.079	$C_4H_{10}N_3O^+$	PZNO
132.076	132.077	132.075	132.074	132.074	$C_4H_{10}N_3O_2^+$	$PZNO_2$
		145.072	145.071	145.071	$C_4H_{10}N_4O_2^+$	Dinitroso-piperazine sample impurity

[a] ADA, data from the ADA-project, Ref. 25 in the main text. [b] Only ion signals changing in intensity by more than 1% of the change in 1-nitrosopiperazine ion signal are included. Ions signals related to cyclohexane and cyclohexanone are not included. [c] *m/z* in red font corresponds to ion signals decreasing in intensity during the experiments.

25. Summary Report: Photo-oxidation of Methylamine, Dimethylamine and Trimetahylamine. Climit project no. 201604, Summary Report: Photo-oxidation of Methylamine, Dimethylamine and Trimetahylamine. Climit project no. 201604; NILU OR 2/2011, ISBN 978-82-425-2357-0; NILU: 2011.

Piperazine photo-oxidation studies

The initial experimental conditions of the piperazine experiments are listed in Table S10. The full documentation from monitors is available upon request.

Table S10.

Initial experimental conditions in piperazine photo-oxidation experiments.

Exp. No.	Date	Experiment description	PZ [ppbv]	IPN (rate, total) [µl min^{-1}; µl]	NO [ppbv]	NO_2 [ppbv]	RH [%]	T [K]
1	2010.03.04		150	0	15	50	0.7	286
2	2010.07.20	H_2O_2 as OH precursor	260	0	15	65	2.1	295
3	2010.07.21		200	0	20	75	2.0	295
4	2010.07.22		200	0	15	140	1.9	298
5	2011.04.05	IPN-d6 as OH precurson	220	100 µl	40	35	0.5	287
6	2011.04.06		220	0	100	100	9.0	290
7	2011.06.09 am		290	0	0	15	3.0	285
8	2011.06.09 pm		320	0	5	80	1.1	298
9	20.07.2016	210 ppbv NO_x	195	0	97	110	2.7	300
10	21.07.2016	159 ppbv NO_x	210	0	81	64	0.3	302
11	22.07.2016	slow IPN, 60 ppbv NO	245	0.1 (20 mins), 0.3 (70 mins), 0.2 continue	0	0	0.3	297
12	25.07.2016 am	quick IPN, 60 ppbv NO	220	3; 135	60	0	1.2	298
13	25.07.2016 pm	quick IPN, 90 ppbv NO	220	3; 75	84	0	0.9	301
14	26.07.2016	quick IPN, 90 ppbv NO_2	183	3; 115	0	67	1.7	297
15	26.07.2016	quick IPN, 40 ppbv NO_2	165	3; 88	0	29	1	302

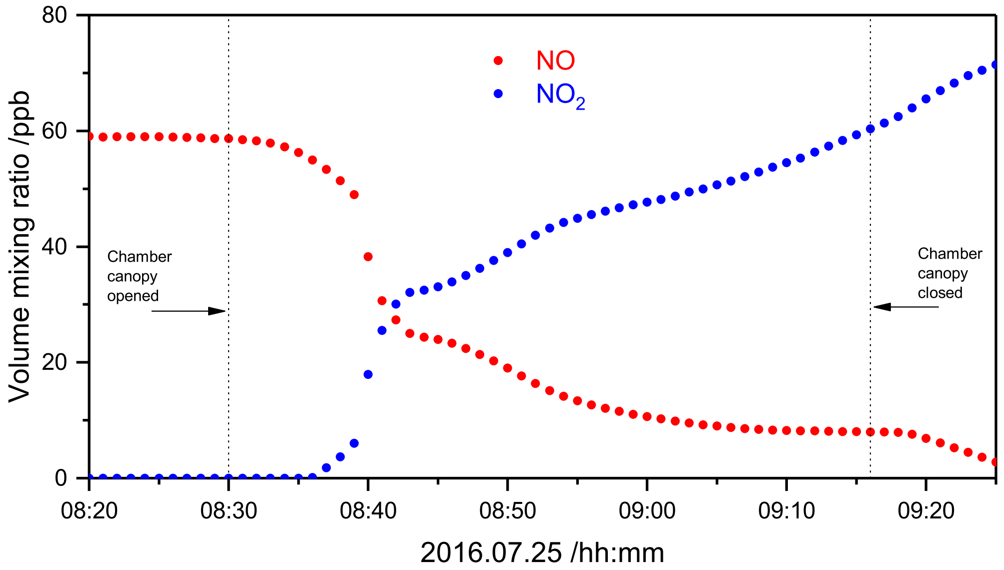

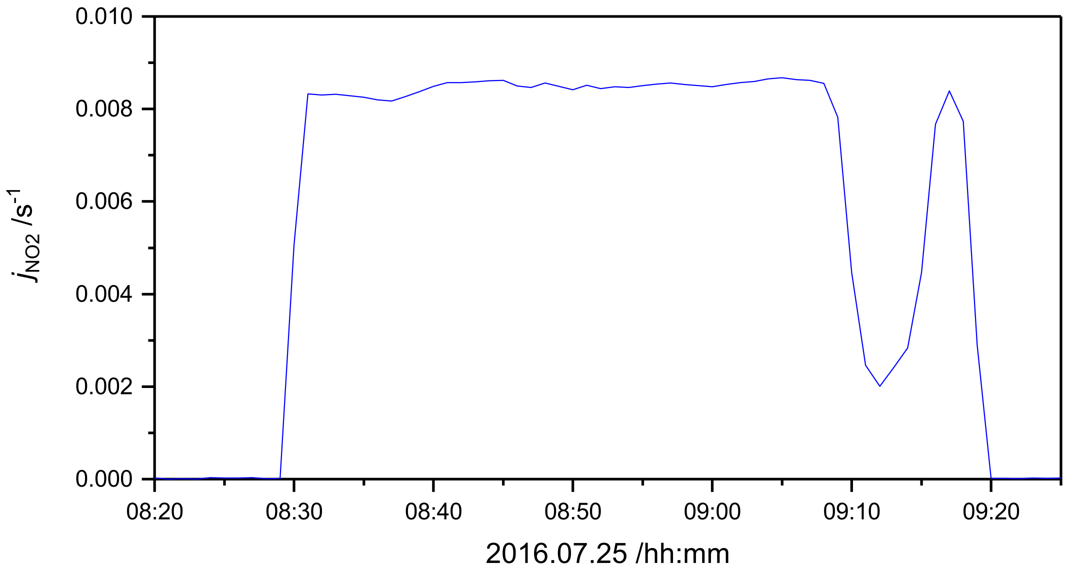

Figure S18.
Chamber conditions during the OH-initiated piperazine photo-oxidation experiment on 2016.07.25. Top: NO and NO_2 volume mixing ratios. Bottom: Photolysis frequency, j_{NO2}.

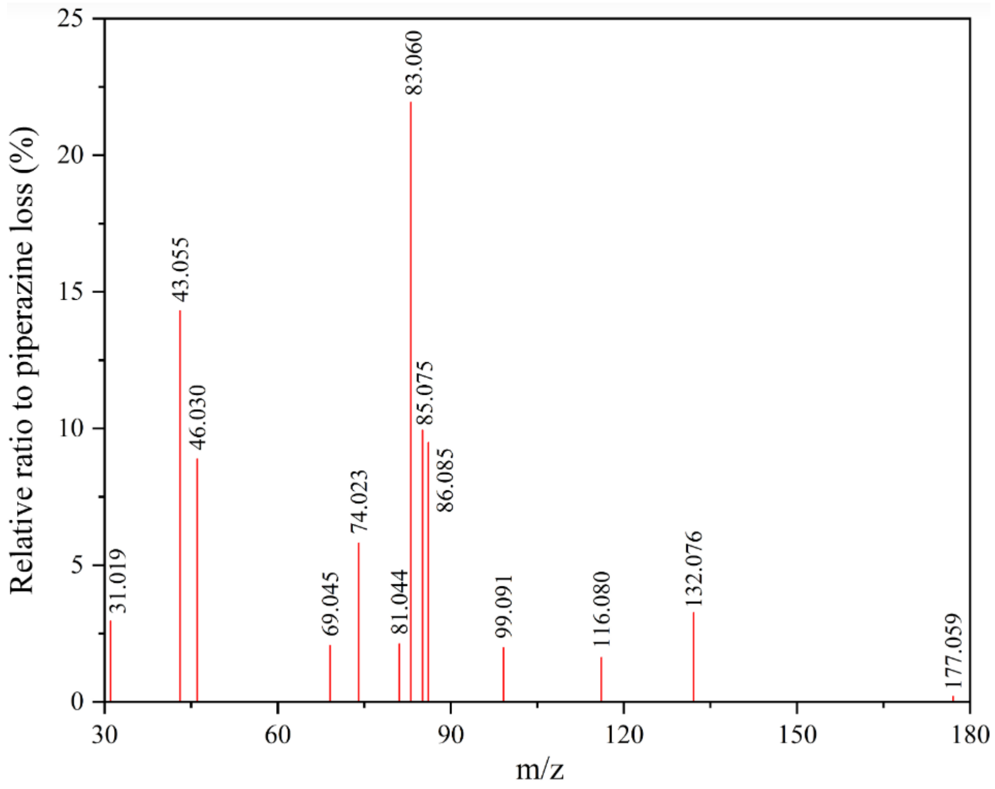

Figure S19.
Observed ion signals during the piperazine experiment on 2016.07.25.

Scheme S2.
Formation of formamide and imidazole from rearrangement reactions of $CHONHCH_2OH$, $CHONHCH_2CH_2NHCHO$ and hydrolysed $CHONHCH_2CH_2N{=}CH_2$ in the aerosol.

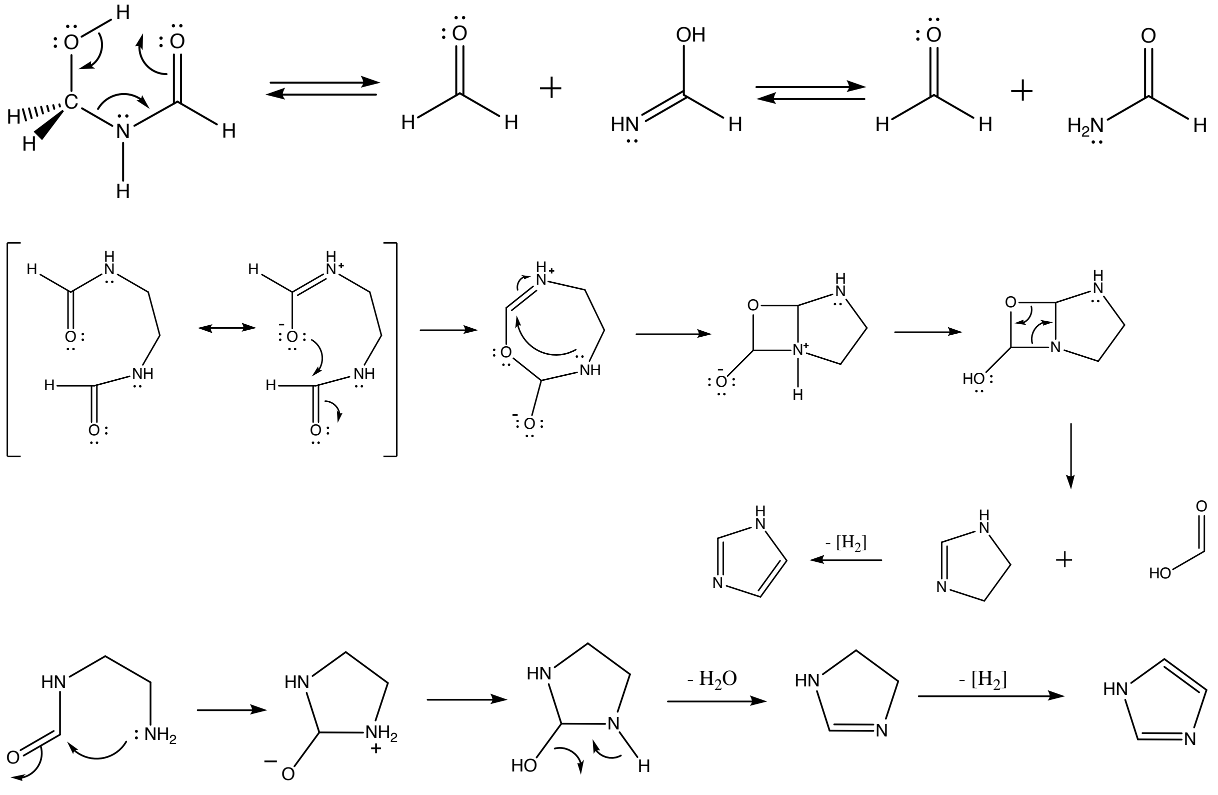

Table S11.

Mass peaks observed during piperazine photo-oxidation experiments. [a]

m/z	ADA Experiment No.[b] 1	2	3	4	5	6	7	8	2016 07.20	2016 07.21	2016 07.22	2016 07.25 am	2016 07.25 pm	2016 07.26 am	2016 07.26 pm	Ion sum formula		Interpretation
43.054											43.054	43.055	43.055	43.055	43.053	$C_3H_7^+$		Fragment
44.050	x	x	x	x						44.051		44.057		44.051	44.051	$C_2H_6N^+$		Fragment from $[PZ]H^+$, $[PZNO]H^+$, $[PZNO_2]H^+$, $[PZI]H^+$
46.029	x	x	x	x	x	x	x	x	46.030	46.030	46.030	46.032	46.030	46.030	46.030	CH_4NO^+	* [c]	Fragment, NH_2CHO, or HN=CHOH
69.0453	x	x	x	x				x	69.046	69.046	69.045	69.044	69.045	69.045	69.045	$C_3H_5N_2^+$		Imidazole and isomers from heteroneous reactions
74.0242	x	x	x	x	x	x	x	x	74.023	74.024	74.023	74.025	74.024	74.023	74.023	$C_2H_4NO_2^+$	*	CHONHCHO
81.0453	x	x	x	x	x			x			81.044	81.045	81.045	81.044	81.044	$C_4H_5N_2^+$	*	Pyrazine
83.0609	x	x	x	x	x	x	x	x	83.060	83.060	83.060	83.061	83.060	83.060	83.060	$C_4H_7N_2^+$	*	Dihydropyrazines, fragment from $[PZI]H^+$
85.0766	x	x	x	x				x	85.076	85.076		85.077	85.076	85.074	85.074	$C_4H_9N_2^+$		Fragment from $[PZ]H^+$, $[PZI]H^+$, $[PZNO]H^+$ and $[PZNO_2]H^+$
86.0844		x	x		x	x	x	x	86.090	86.090	86.088	86.087		86.084	86.084	$C_4H_{10}N_2^+$		Fragment from $[PZNO]H^+$ and $[PZNO_2]H^+$
87.0922	x	x	x	x	x	x	x	x	87.091	87.091	87.091	87.092	87.090	87.091	87.091	$C_4H_{11}N_2^+$		PZ
99.0558		x	x	x	x			x			99.054		99.056	99.054	99.066	$C_4H_7N_2O^+$	*	Dihydropyrazinone isomers, oxidation product of PZI?
99.0922	x	x	x	x	x	x		x		99.090		99.091	99.092			$C_5H_{11}N_2^+$	*	Unidentified condensation product
115.0871		x	x			x		x	115.084	115.085	115.083		115.086			$C_5H_{11}N_2O^+$	*	Piperazine-1-carbaldehyde [e]
116.0824		x	x		x				116.080	116.081		116.081	116.079			$C_4H_{10}N_3O^+$	*	PZ-NO
132.0773		x	x		x	x	x	x	132.076	132.076		132.075	132.075	132.075	132.076	$C_4H_{10}N_3O_2^+$	*	$PZNO_2$
177.0624												177.059				$C_4H_9N_2O_4^+$		$PZ(NO_2)_2$

[a] Only ion signals changing in intensity by more than 2% of the change in piperazine *m/z* 87.091 signal are included. [b] ADA, data from the ADA-project, Ref. 29 in the main text. [c] Molecular formula found by TD-GCMS of Tenax samples, Ref. 8 in the main text.

8. White, S.; Angove, D.; Azzi, M.; Tibbett, A.; Campbell, I.; Patterson, M. An experimental investigation into the atmospheric degradation of piperazine. Atmos. Environ. 2015, 108, 133-139.
29. Summary Report: Photo-oxidation of Methylamine, Dimethylamine and Trimetahylamine. Climit project no. 201604, Summary Report: Photo-oxidation of Methylamine, Dimethylamine and Trimetahylamine. Climit project no. 201604; NILU OR 2/2011, ISBN 978-82-425-2357-0; NILU: 2011.

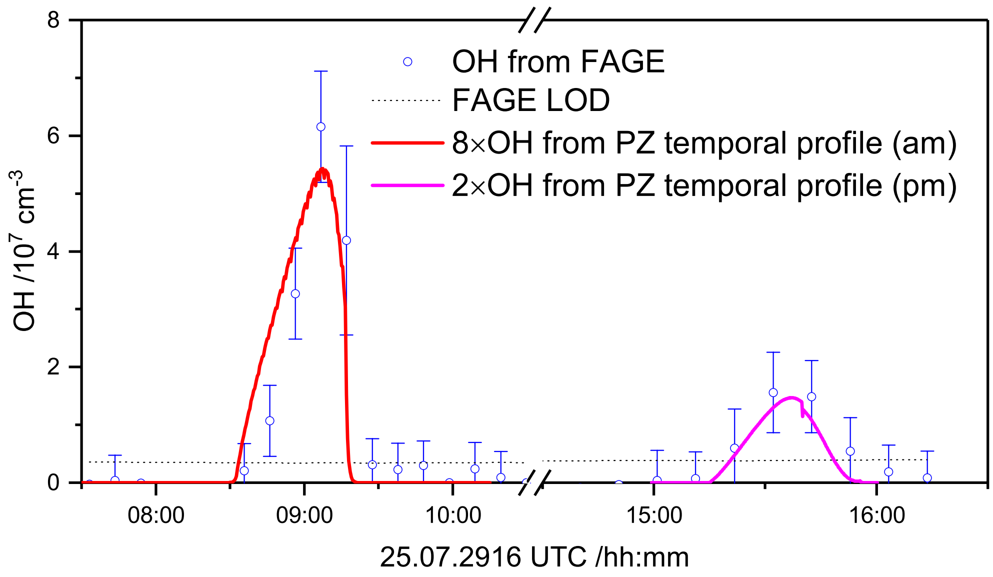

Figure S20.
OH concentrations from FAGE measurements and from analyses of the PZ temporal profiles during photo-oxidation experiments on 2016.07.25.

The OH concentration derived from the tracer decays is significantly lower than for the measured OH. There are several possible explanations for this. The first is that the comparison assumes that the chamber is well mixed. However, because both instruments were sampling from different locations, and both were sampling near the outer edges of the chamber, there is the possibility of poor mixing at the edges resulting in an inhomogeneous distribution of both the OH and the amine species. However, the EUPHORE chamber uses fans to circulate the air in the chamber, which should have ensured that the chamber should be well mixed. The second is that there is an unrecognised interference in the FAGE cell under the operational conditions in Valencia caused by an unknown species in the chamber that is contributing towards the OH signal. These are species that may generate OH in the detection cell either when photolysed by the 308 nm laser light, or following their decomposition within the cell after they are sampled and drawn into the low pressure cell. [38] No testing has been performed for the Leeds aircraft cell for any potential interferences from either gaseous or aerosol phase amine oxidation products, and the aircraft-based instrument is not equipped with an inlet pre-injector (IPI),[39] so it is not possible to rule out or quantify any such interference.

Particle analysis during the piperazine + OH reaction

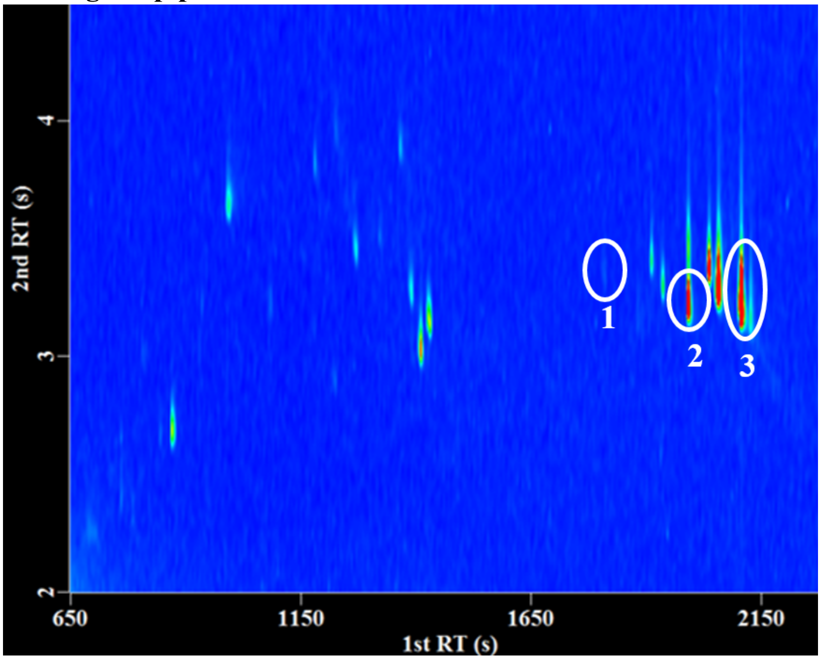

Figure S21.
GC×GC-NCD chromatogram of an aerosol sample collected during a piperazine photo-oxidation experiment. The circled peaks are 1,4-dinitrosopiperazine (1), derivatised *N*-nitrosopiperazine (2) and derivatised *N*-nitropiperazine (3). Several other unidentified organic nitrogen species were detected.

Table S12.
Mass ratios of 1-nitrosopiperazine, 1-nitropiperazine and 1,4-dinitrosopiperazine detected on the filter samples obtained during PZ photo-oxidatrion experiments.

Date	Sampling time (UTC)	Nitramine / nitrosamine	Nitramine / dinitrosamine	Nitrosamine / dinitrosamine
**20.07.2016**	06:26 – 06:56	n.d.	n.d.	n.d.
	09:52 – 10:52*	3	128	40
	11:01 – 12:01*	21	1046	49
	12:45 – 13:45	42	2654	63
**21.07.2016**	06:22 – 06:52	n.d.	n.d.	n.d.
	11:42 – 12:27*	10	616	64
	12:32 – 13:02*	15	1077	72
	13:07 – 13:19*	12	629	51
	13:37 – 14:37	18	963	53
**22.07.2016**	06:35 – 07:05	n.d.	n.d.	n.d.
	09:30 – 10:30*	37	n.d.	n.d.
	10:44 – 11:15*	26	n.d.	n.d.
	11:49 – 12:49	15	n.d.	n.d.
**25.07.2016**	06:31 – 07:01	n.d.	n.d.	n.d.
	08:35 – 09:15*	6	234	40
	09:54 – 10:54	29	439	15
**26.07.2016**	06:29 – 06:59	n.d.	n.d.	n.d.
	08:55 – 09:30*	11	330	31
	10:06 – 10:51	33	365	11

*denotes sampling intervals during which the chamber canopy was open. n.d. refers to one or both of the compounds not detected and therefore ratio not calculated. Reported ratios are based on raw data that has not been corrected for particle wall loss and chamber dilution.

Implications

Amine photo-oxidation, and nitrosamine and nitramine formation was simulated according to the chemistry model presented in Scheme S3; the rate coefficients employed are summarized in Tables S13 and S14. The NOx-conditions in the urban background air in the Oslo region is $\langle NO \rangle_{annual}$ ~6 ppb and $\langle NO_2 \rangle_{annual}$ ~10 ppb.[40] The annual average OH concentration and NO_2 photolysis frequency have been taken as $\langle OH \rangle_{annual} = 10^{-6}$ cm^{-3} and $\langle j_{NO_2} \rangle_{annual} = 1.3 \times 10^{-3}$ s^{-1}.[41] Figure S21 compares the results from simple box-models. Note that the MEA-model by Karl et al.[42] includes the primary nitrosamine that, according to theory,[43-44] is formed, but which has not been observed in experiments, and that the authors state their model to overestimate the amount of nitramine formed by "at leat a factor of 5".[42]

Scheme S3.

Chemistry model for nitrosamine and nitramine formation in atmospheric amine photo-oxidation.

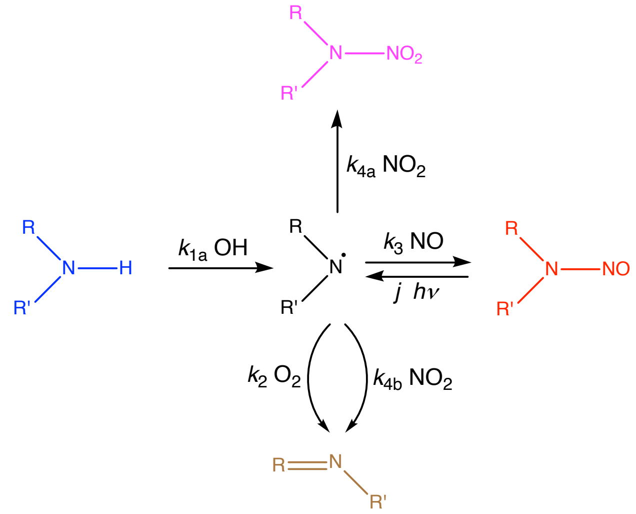

Table S13.

Rate coefficients (/10^{-11} cm^3 molecule^{-1} s^{-1}) for amine reactions with OH and relative rate coefficients relevant to the branching in aminyl radical reactions with O_2, NO and NO_2. See Scheme 2 for definitions.

	k_1	k_{1a}/k_1	k_2/k_{4a} / 10^{-7}	k_{4b}/k_{4a}	k_3/k_{4a}	$j_{rel} = j / j_{NO2}$	Reference
DMA [a]	6.8	0.41	3.90	0.22	0.26	0.34	[15]
MEA [b]	7.6	0.15	2.43	1	1.21	0.33	[42]
PZ [c]	23	0.18	1.57	0	1.70	0.34	This work

[a] DMA (dimethylamine), k_1 from Ref. 45, k_{1a}/k_1 from Ref. 46; [b] MEA (monoethanolamine), k_1 from Ref. 47; [c] PZ (piperazine), k_1 from Ref. 34.

Table S14.

Rate coefficients (/10^{-11} cm^3 molecule^{-1} s^{-1}) for nitrosamine and nitramine reactions with OH.

	DMA		MEA		PZ	
	Nitrosamine	Nitramine	Nitrosamine	Nitramine	Nitrosamine	Nitramine
k_{OH}	0.30	0.35		1.48 [a]	12 [a]	12 [a]
Reference	48	48-49		42	This work	This work

[a] Estimated value

References

(1) Becker, K. H., *The European Photoreactor EUPHORE: Design and Technical Development of the European Photoreactor and First Experimental Results: Final Report of the EC-Project: Contract EV5V-CT92-0059: Funding Period, January 1993-December 1995*. 1996.

(2) Jordan, A.; Haidacher, S.; Hanel, G.; Hartungen, E.; Märk, L.; Seehauser, H.; Schottkowsky, R.; Sulzer, P.; Märk, T. D. A high resolution and high sensitivity proton-transfer-reaction time-of-flight mass spectrometer (PTR-TOF-MS). *International Journal of Mass Spectrometry* **2009,** *286*, 122-128.

(3) Eichler, P.; Muller, M.; D'Anna, B.; Wisthaler, A. A novel inlet system for online chemical analysis of semi-volatile submicron particulate matter. *Atmospheric Measurement Techniques* **2015,** *8*, 1353-1360.

(4) Eichler, P.; Müller, M.; Rohmann, C.; Stengel, B.; Orasche, J. r.; Zimmermann, R.; Wisthaler, A. Lubricating Oil as a Major Constituent of Ship Exhaust Particles. *Environmental Science & Technology Letters* **2017,** *4*, 54-58.

(5) Drewnick, F.; Hings, S. S.; DeCarlo, P.; Jayne, J. T.; Gonin, M.; Fuhrer, K.; Weimer, S.; Jimenez, J. L.; Demerjian, K. L.; Borrmann, S., et al. A New Time-of-Flight Aerosol Mass Spectrometer (TOF-AMS)—Instrument Description and First Field Deployment. *Aerosol Sci. Technol.* **2005,** *39*, 637-658.

(6) Müller, M.; George, C.; D'Anna, B. Enhanced spectral analysis of C-TOF Aerosol Mass Spectrometer data: Iterative residual analysis and cumulative peak fitting. *Int. J. Mass Spectrom.* **2011,** *306*, 1-8.

(7) Farren, N. J.; Ramírez, N.; Lee, J. D.; Finessi, E.; Lewis, A. C.; Hamilton, J. F. Estimated Exposure Risks from Carcinogenic Nitrosamines in Urban Airborne Particulate Matter. *Environ. Sci. Technol.* **2015,** *49*, 9648-9656.

(8) Stone, D.; Whalley, L. K.; Heard, D. E. Tropospheric OH and HO2 radicals: field measurements and model comparisons. *Chem. Soc. Rev.* **2012,** *41*, 6348-6404.

(9) Heard, D. E. Atmospheric Field Measurements of the Hydroxyl Radical using Laser-Induced Fluorescence Spectroscopy. *Ann. Rev. Phys. Chem.* **2006,** *57*, 191-216.

(10) Commane, R.; Floquet, C. F. A.; Ingham, T.; Stone, D.; Evans, M. J.; Heard, D. E. Observations of OH and HO_2 radicals over West Africa. *Atmos. Chem. Phys.* **2010,** *10*, 8783-8801.

(11) Emmons, W. D.; Freeman, J. P. Alkaline nitration. III. The reaction of acetone cyanohydrin nitrate with metal alkoxides. *J. Am. Chem. Soc.* **1955,** *77*, 4673-4.

(12) Liu, L.; Xu, Y.; Shea, C.; Fowler, J. S.; Hooker, J. M.; Tonge, P. J. Radiosynthesis and Bioimaging of the Tuberculosis Chemotherapeutics Isoniazid, Rifampicin and Pyrazinamide in Baboons. *J. Med. Chem.* **2010,** *53*, 2882-2891.

(13) Cox, R. F. B.; Stormont, R. T. Acetone cyanohydrin. *Org. Synth.* **1935,** *XV*, 1-2.

(14) Lazarou, Y. G.; Kambanis, K. G.; Papagiannakopoulos, P. Gas-Phase Reactions of $(CH_3)_2N$ Radicals with NO and NO_2. *J. Phys. Chem.* **1994,** *98*, 2110-2115.

(15) Lindley, C. R. C.; Calvert, J. G.; Shaw, J. H. Rate Studies of the Reactions of the $(CH_3)_2N$ Radical with O_2, NO, and NO_2. *Chem. Phys. Lett.* **1979,** *67*, 57-62.

(16) Atkinson, R.; Baulch, D. L.; Cox, R. A.; Crowley, J. N.; Hampson, R. F.; Hynes, R. G.; Jenkin, M. E.; Rossi, M. J.; Troe, J. Evaluated kinetic and photochemical data for atmospheric chemistry: Volume I - gas phase reactions of O(x), HO(x), NO(x) and SO(x) species. *Atmos. Chem. Phys.* **2004,** *4*, 1461-1738.

(17) Peiró-García, J.; Nebot-Gil, I.; Merchán, M. An Ab Initio Study on the Mechanism of the Atmospheric Reaction $NH_2+O_3 \rightarrow H_2NO+O_2$. *ChemPhysChem* **2003,** *4*, 366-372.

(18) Peiró–García, J.; Ramírez–Ramírez, V. M.; Nebot–Gil, I. A theoretical ab initio study on the $H_2NO + O_3$ reaction. *Journal of Computational Chemistry* **2003,** *24*, 1321-1328.

(19) Atkinson, R.; Baulch, D. L.; Cox, R. A.; Crowley, J. N.; Hampson, R. F.; Hynes, R. G.; Jenkin, M. E.; Rossi, M. J.; Troe, J. Evaluated Kinetic and Photochemical Data for Atmospheric Chemistry: Volume II - Gas Phase Reactions of Organic Species. *Atmos. Chem. Phys.* **2006,** *6*, 3625-4055.

(20) Wallington Timothy, J. Kinetics of the gas phase reaction of OH radicals with pyrrole and thiophene. *Int. J. Chem. Kinet.* **2004,** *18*, 487-496.

(21) Atkinson, R.; Aschmann, S. M.; Winer, A. M.; Carter, W. P. L. Rate constants for the gas phase reactions of OH radicals and O3 with pyrrole at 295 ± 1 K and atmospheric pressure. *Atmos. Environ.* **1984,** *18*, 2105-2107.

(22) Dillon, T. J.; Tucceri, M. E.; Dulitz, K.; Horowitz, A.; Vereecken, L.; Crowley, J. N. Reaction of Hydroxyl Radicals with C_4H_5N (Pyrrole): Temperature and Pressure Dependent Rate Coefficients. *J. Phys. Chem. A* **2012,** *116*, 6051-6058.

(23) Atkinson, R. Kinetics and Mechanisms of the Gas-Phase Reactions of the Hydroxyl Radical with Organic Compounds under Atmospheric Conditions. *Chem. Rev.* **1986,** *86*, 69-201.

(24) Bohn, B.; Zetzsch, C. Kinetics and mechanism of the reaction of OH with the trimethylbenzenes - experimental evidence for the formation of adduct isomers. *Phys. Chem. Chem. Phys.* **2012,** *14*, 13933-13948.

(25) Aschmann, S. M.; Long, W. D.; Atkinson, R. Temperature-Dependent Rate Constants for the Gas-Phase Reactions of OH Radicals with 1,3,5-Trimethylbenzene, Triethyl Phosphate, and a Series of Alkylphosphonates. *J. Phys. Chem. A* **2006,** *110*, 7393-7400.

(26) Kramp, F.; Paulson, S. E. On the Uncertainties in the Rate Coefficients for OH Reactions with Hydrocarbons, and the Rate Coefficients of the 1,3,5-Trimethylbenzene and m-Xylene Reactions with OH Radicals in the Gas Phase. *J. Phys. Chem. A* **1998,** *102*, 2685-2690.

(27) Atkinson, R.; Aschmann Sara, M. Rate constants for the gas - phase reactions of the OH radical with a series of aromatic hydrocarbons at 296 ± 2 K. *Int. J. Chem. Kinet.* **2004,** *21*, 355-365.

(28) Ohta, T.; Ohyama, T. A set of rate constants for the reactions of hydroxyl radicals with aromatic hydrocarbons. *Bull. Chem. Soc. Jpn.* **1985,** *58*, 3029-30.

(29) Perry, R. A.; Atkinson, R.; Pitts, J. N., Jr. Kinetics and mechanism of the gas phase reaction of hydroxyl radicals with aromatic hydrocarbons over the temperature range 296-473 K. *J. Phys. Chem.* **1977,** *81*, 296-304.

(30) Hansen, D. A.; Atkinson, R.; Pitts, J. N., Jr. Rate constants for the reaction of hydroxyl radicals with a series of aromatic hydrocarbons. *J. Phys. Chem.* **1975,** *79*, 1763-6.

(31) Winer, A. M.; Lloyd, A. C.; Darnall, K. R.; Pitts, J. N., Jr. Relative rate constants for the reaction of the hydroxyl radical with selected ketones, chloroethenes, and monoterpene hydrocarbons. *J. Phys. Chem.* **1976,** *80*, 1635-9.

(32) Braure, T.; Bedjanian, Y.; Romanias, M. N.; Morin, J.; Riffault, V.; Tomas, A.; Coddeville, P. Experimental Study of the Reactions of Limonene with OH and OD Radicals: Kinetics and Products. *J. Phys. Chem. A* **2014,** *118*, 9482-9490.

(33) Gill, K. J.; Hites, R. A. Rate Constants for the Gas-Phase Reactions of the Hydroxyl Radical with Isoprene, α- and β-Pinene, and Limonene as a Function of Temperature. *J. Phys. Chem. A* **2002,** *106*, 2538-2544.

(34) Onel, L.; Dryden, M.; Blitz, M. A.; Seakins, P. W. Atmospheric Oxidation of Piperazine by OH has a Low Potential To Form Carcinogenic Compounds. *Environ. Sci. Technol. Lett.* **2014,** *1*, 367-371.

(35) Nielsen, C. J.; D´Anna, B.; Bossi, R.; Bunkan, A. J. C.; Dithmer, L.; Glasius, M.; Hallquist, M.; Hansen, A. M. K.; Lutz, A.; Salo, K., et al. *Atmospheric Degradation of Amines (ADA)*; ISBN 978-82-992954-7-5, http://urn.nb.no/URN:NBN:no-30510; University of Oslo: Oslo, 2012.

(36) Atkinson, R. Kinetics of the gas-phase reactions of OH radicals with alkanes and cycloalkanes. *Atmospheric Chemistry and Physics* **2003,** *3*, 2233-2307.

(37) Dagaut, P.; Wallington, T. J.; Liu, R. Z.; Kurylo, M. J. A kinetics investigation of the gas-phase reactions of OH radicals with cyclic-ketones and -diones - mechenistic insights. *Journal of Physical Chemistry* **1988,** *92*, 4375-4377.

(38) Mao, J.; Ren, X.; Zhang, L.; Van Duin, D. M.; Cohen, R. C.; Park, J. H.; Goldstein, A. H.; Paulot, F.; Beaver, M. R.; Crounse, J. D., et al. Insights into hydroxyl measurements and atmospheric oxidation in a California forest. *Atmos. Chem. Phys.* **2012,** *12*, 8009-8020.

(39) Woodward-Massey, R.; Slater, E. J.; Alen, J.; Ingham, T.; Cryer, D. R.; Stimpson, L. M.; Ye, C.; Seakins, P. W.; Whalley, L. K.; Heard, D. E. Implementation of a chemical background method for atmospheric OH measurements by laser-induced fluorescence:

characterisation and observations from the UK and China. *Atmos. Meas. Tech.* **2020,** *13*, 3119-3146.

(40) NILU Luftkvalitet.info. http://www.luftkvalitet.info/home.aspx (accessed Sept. 27, 2020).

(41) Wolke, R.; Schrödner, R. *Atmospheric Chemistry – Dark Chemistry. Nighttime Chemistry in the Mongstad Area: Literature Study and Model Simulations*; Report no. 2211030-DC01; Tel-Tek: 2011.

(42) Karl, M.; Dye, C.; Schmidbauer, N.; Wisthaler, A.; Mikoviny, T.; D'Anna, B.; Müller, M.; Borrás, E.; Clemente, E.; Muñoz, A., et al. Study of OH-initiated degradation of 2-aminoethanol. *Atmos. Chem. Phys.* **2012,** *12*, 1881-1901.

(43) Tang, Y.; Hanrath, M.; Nielsen, C. J. Do primary nitrosamines form and exist in the gas phase? A computational study of CH3NHNO and (CH3)2NNO. *Phys. Chem. Chem. Phys.* **2012,** *14*, 16365-16370.

(44) da Silva, G. Formation of Nitrosamines and Alkyldiazohydroxides in the Gas Phase: The CH_3NH + NO Reaction Revisited. *Environ. Sci. Technol.* **2013,** *47*, 7766-7772.

(45) McGillen, M. R.; Carter, W. P. L.; Mellouki, A.; Orlando, J. J.; Picquet-Varrault, B.; Wallington, T. J. Database for the Kinetics of the Gas-Phase Atmospheric Reactions of Organic Compounds. *Earth Syst. Sci. Data* **2020,** *12*, 1203-1216.

(46) Onel, L.; Blitz, M.; Dryden, M.; Thonger, L.; Seakins, P. Branching Ratios in Reactions of OH Radicals with Methylamine, Dimethylamine, and Ethylamine. *Environ. Sci. Technol.* **2014,** *48*, 9935-9942.

(47) Onel, L.; Blitz, M. A.; Seakins, P. W. Direct Determination of the Rate Coefficient for the Reaction of OH Radicals with Monoethanol Amine (MEA) from 296 to 510 K. *The Journal of Physical Chemistry Letters* **2012,** *3*, 853-856.

(48) Tuazon, E. C.; Carter, W. P. L.; Atkinson, R.; Winer, A. M.; Pitts, J. N. Atmospheric Reactions of N-Nitrosodimethylamine and Dimethylnitramine. *Environ. Sci. Technol.* **1984,** *18*, 49-54.

(49) Zabarnick, S. S.; Fleming, J. W.; Baronavski, A. P.; Lin, M. C. Reaction Kinetics of Hydroxyl with Nitromethane, Dimethylnitrosamine, and 1,3,5-Trioxane; Photolytic Production of Hydroxyl from Nitromethane at 266 nm. *NBS Special Publication (United States)* **1986,** *716*, 731-56.
